# SCEL regulates switches between pro-survival and apoptosis of the TNF-α/TNFR1/NF-κB/c-FLIP axis to control lung colonization of triple negative breast cancer

**DOI:** 10.1186/s12929-023-00986-4

**Published:** 2023-11-30

**Authors:** Shih-Hsuan Chan, Wen-Hung Kuo, Lu-Hai Wang

**Affiliations:** 1 School of Chinese Medicine, College of Chinese Medicine, China Medical University, Taichung, Taiwan; 2 Chinese Medicine Research Center, China Medical University, No. 91, Hsueh-Shih Road, Taichung, 40402 Taiwan; 3 Cancer Biology and Precision Therapeutics Center, China Medical University, Taichung, 40402 Taiwan; 4 Department of Surgery, National Taiwan University Hospital, Taipei, 100 Taiwan; 5 Graduate Institute of Integrated Medicine, China Medical University, Taichung, Taiwan

**Keywords:** mTNBC, iTRAQ, SCEL, TNF-α, TNFR1, NF-κB, c-FLIP, Caspase 3, Adalimumab

## Abstract

**Background:**

Patients with metastatic triple-negative breast cancer (mTNBC) have a higher probability of developing visceral metastasis within 5 years after the initial diagnosis. Therefore, a deeper understanding of the progression and spread of mTNBC is urgently needed.

**Methods:**

The isobaric tag for relative and absolute quantitation (iTRAQ)-based LC–MS/MS proteomic approach was applied to identify novel membrane-associated proteins in the lung-tropic metastatic cells. Public domain datasets were used to assess the clinical relevance of the candidate proteins. Cell-based and mouse models were used for biochemical and functional characterization of the protein molecule Sciellin (SCEL) identified by iTRAQ to elucidate its role and underlying mechanism in promoting lung colonization of TNBC cells.

**Results:**

The iTRAQ-based LC–MS/MS proteomic approach identified a membrane-associated protein SCEL that was overexpressed in the lung-tropic metastatic cells, and its high expression was significantly correlated with the late-stage TNBC and the shorter survival of the patients. Downregulation of SCEL expression significantly impaired the 3D colony-forming ability but not the migration and invasion ability of the lung colonization (LC) cells. Knockdown of SCEL reduced TNF-α-induced activation of the NF-κB/c-FLIP pro-survival and Akt/Erk1/2 growth signaling pathways in the LC cells. Specifically, knockdown of SCEL expression switched TNF-α-mediated cell survival to the caspase 3-dependent apoptosis. Conversely, ectopic expression of SCEL promoted TNF-α-induced activation of NF-κB/c-FLIP pro-survival and Akt/Erk1/2 pro-growth signaling pathway. The result of co-immunoprecipitation (Co-IP) and GST pull-down assay showed that SCEL could interact with TNFR1 to promote its protein stability. The xenograft mouse model experiments revealed that knockdown of SCEL resulted in increase of caspase-3 activity, and decrease of ki67 and TNFR1 expression as well as increase of tumor-associated macrophages in the metastatic lung lesions. Clinically, SCEL expression was found to be positively correlated with TNFR1 in TNBC tissues. Lastly, we showed that blocking TNF-α-mediated cell survival signaling by adalimumab effectively suppressed the lung colonization of the SCEL-positive, but not the SCEL-downregulated LC cells in the tail-vein injection model.

**Conclusions:**

Our findings indicate that SCEL plays an essential role in the metastatic lung colonization of TNBC by promoting the TNF-α/TNFR1/NF-κB/c-FLIP survival and Akt/Erk1/2 proliferation signaling. Thus, SCEL may serve as a biomarker for adalimumab treatment of TNBC patients.

**Graphical abstract:**

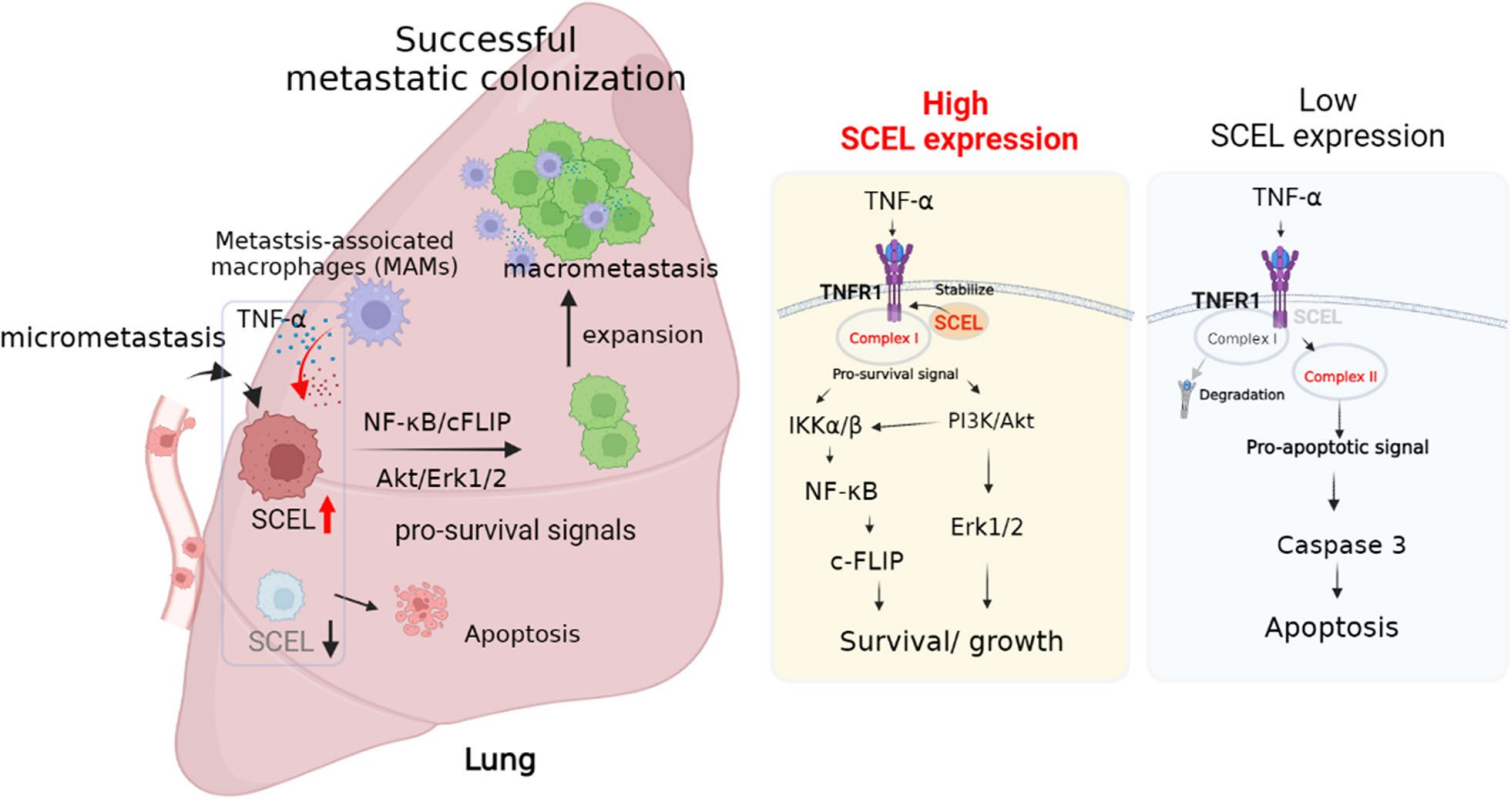

**Supplementary Information:**

The online version contains supplementary material available at 10.1186/s12929-023-00986-4.

## Background

In 2021, breast cancer (BC) has officially surpassed lung cancer to become the most common cancer worldwide [1]. Triple-negative breast cancer (TNBC), an aggressive subtype of BC, is the most difficult-to treat BC by far and poses a major threat to the patients’ survival [2]. With no hormone receptor and human epidermal growth factor receptor 2 (ERBB2/HER2) expression, the treatment of TNBC patients has been limited due to the lack of effective targeted therapies. As a result, patients with advanced TNBC have a higher frequency of developing visceral metastasis within 3 years after failure to conventional chemotherapy compared to those with other BC subtypes [2, 3]. According to the meta-analysis of 17 population-based cancer registries containing 196,064 invasive BC in the SEER (Surveillance, Epidemiology, and End Results) program, four-year overall survival of patients with stage IV metastatic TNBC (mTNBC) was as low as 11.2% [2, 4–6]. Recently, the poly (adenosine diphosphate–ribose) polymerase (PARP) inhibitor has been shown to be effective in TNBC patients carrying BRCA1/2 mutations. A phase I/II study KEYNOTE-162 showed that the PARP inhibitor niraparib plus pembrolizumab, an immune check point therapeutic antibody, provides clinical benefit in patients with advanced or metastatic TNBC that the BRCA1/2 mutation carriers showed higher objective response rate compared to the non-carriers (47% vs 11%) [7]. However, only 10–20% of patients with TNBC carry BRCA1/2 mutations who could benefit from PARPi treatment [8, 9].

Immune checkpoint blockade therapy has shown promising therapeutic efficacy in multiple types of cancers [10]. A phase III study KEYNOTE-522 showed that pembrolizumab plus neoadjuvant chemotherapy improved TNBC patients’ pathological complete response (pCR) by 13.6% compared to placebo plus neoadjuvant therapy [11]. However, a phase Ib study reported that patients with advanced TNBC responded poorly to the single agent pembrolizumab therapy (overall response rate = 18.5%), which may be explained by the low percentage of PD-L1 (programmed death ligand-1) expression (< 20%) in the TNBC population [12–14]. Despite much effort being devoted, a more effective targeted therapy for TNBC patients with metastatic disease is still urgently needed.

Metastatic colonization in the vital organs is the major cause of death in patients with advanced or metastatic TNBC. Therefore, a better understanding of the factors affecting the disseminated TNBC cells’ survival, dormancy, and progression at the metastatic site could shed light on the development of more effective therapeutic approaches to improve patients’ survival [15]. Previous studies have shown that, when arriving at a distant organ, the disseminated tumor cells (DTCs) that possess EMT (epithelial–mesenchymal transition) properties would undergo the reverse cellular transformation called “mesenchymal-epithelial transition (MET)” to facilitate their settling down, anchoring, and survival at the foreign soil, and become the seeds of metastasis [16, 17]. The activation of pro-survival signals, such as PI3K (phosphoinositide 3-kinase)-Akt/PKB (protein kinase B), MAPK (mitogen-activated protein kinase), NF-κB (nuclear factor kappa-light-chain-enhancer of activated B cells), and mTOR (mammalian target of rapamycin), in DTCs have been known to be activated by different types of niches, such as growth factor, extracellular matrix (ECM) and immunosuppressive immune cells Tregs (regulatory T cells), myeloid-derived suppressor cells (MDSCs), neutrophils, and macrophages [16]. However, mechanisms underlying how the metastatic cells enhance the pro-survival signals when interacting with the various niches to overcome anoikis and apoptosis, and ultimately thrive in the distant organs is poorly understood.

Previously, we reported that the lung colonization (LC) cells, established from the long-term lung metastases of tumor-bearing mouse, had great lung colonization capability when re-injected into the tail vein of the mouse [18]. In this study, we employed the isobaric tags for relative and absolute quantitation (iTRAQ)-based proteomic approach to systemically compare the membrane proteome profile between parental MDA-MB-231-primary tumor (PT) and LC cells and identified a membrane-associated protein Sciellin (SCEL) that was significantly correlated with the late-stage and poor prognosis of TNBC. We showed that SCEL expression was essential for TNBC cells to effectively colonize the mouse lung in the mouse model. Mechanistically, we demonstrated that SCEL could promote metastatic lung colonization by enhancing the TNF-α-driven NF-κB/c-FLIP (Cellular FLICE (FADD-like IL-1β-converting enzyme)-inhibitory protein) survival signal and Akt/Erk1/2 growth pathway while suppressing the apoptotic signal. Finally, we demonstrated that anti-TNF-α therapy using adalimumab could effectively reduce lung metastasis of SCEL-positive lung-tropic TNBC cells in the tail vein injection mouse model. Taken together, our study sheds new light on how disseminated TNBC cells colonize the lung by taking advantage of TNF-α-mediated NF-κB/c-FLIP anti-apoptotic signaling and Akt/Erk1/2 promoted proliferation. Moreover, our result suggests that SCEL could serve as a novel treatment biomarker for anti-TNF-α therapy of TNBC patients.

## Methods

### Cell lines and culture conditions

Hs578T, MDA-MB-231-PT cells, IV2 cells [23], and LC cells [19] were maintained in DMEM culture medium containing 10% fetal bovine serum, 2 mM l-glutamine, and 1% penicillin/streptomycin and cultured in a humidified incubator at 37 ℃ supplied with 5% CO_2._ All cell lines have tested negative for mycoplasma contamination and the 231-derived sublines have been authenticated by STR profiling (Additional file 2: Figs. S6, S7, S8). The 231-derived sublines have been publicly deposited at the Bioresource Collection and Research Center (BCRC) at the Food Industry Research and Development Institute in Hsinchu, Taiwan (Additional file 2: Fig. S9).

### Protein fractionation

MDA-MB-231-PT, IV2, and LC cells were grown in the 10-cm culture dishes at a 90% confluence and subjected to the cellular protein fractionation using the cell fractionation kit (Cell Signaling Technology, Danvers, MA, USA) according to the manufacturer’s instruction.

### Transwell migration/invasion assay

For migration assay, 15,000 cells responded in 0.1% bovine serum albumin and were seeded onto the upper chamber of the 24-well format Transwell inserts with the 8.0 μm porous membrane at the bottom. The complete medium with 10% FBS was added to the lower chamber as the source of chemoattractant. The number of cells that migrated through the porous membrane was then counted. For invasion assay, the experiment was similarly performed except the Transwell insert was further pre-coated with a layer of Matrigel (ThermoFisher, CA, USA). The number of cells that have invaded through the Matrigel was then counted.

### Soft agar colony formation assay

1.2% agar was melted and kept in 42 ℃ water bath. To make the base agar, equal volumes of pre-warmed 2 × medium containing 20% FBS were mixed with pre-warmed 1.2% agar and a 1.5 mL mixture was added to the 6-well plate. To make the top agar containing cancer cells, 0.6% agar was melted and sat in 42 ℃ water bath prior to the experiment. 5000 cells were resuspended in 0.75 mL 2 × medium containing 20% FBS and mixed with an equal volume of the pre-warmed 0.6% agar to form 0.3% top agar. After the top agar was solidified, 1 mL complete medium was added on top of the top agar and the cells were cultured at 37 ℃ in the humidified incubator for 14 days followed by staining with iodonitrotetrazolium chloride (Sigma Aldrich, CA, USA).

### NF-κB luciferase reporter assay

Cells were co-transfected with the NF-κB luciferase reporter plasmid and the Renilla luciferase plasmid for 24 h followed by TNF-α simulation. The NF-κB luciferase reporter plasmid contains a firefly luciferase gene driven by four copies of NF-κB response elements, and the Renilla luciferase plasmid was used to normalize the transfection efficiency. The transfected cells were cultured in the presence of 25 ng/mL TNF-α for 24 h followed by luciferase reporter assay. Briefly, cells were lysed in the protein lysis buffer and luciferin substrate was added to the protein lysate. The luciferase activity was measured and recorded using BioTek Synergy H1 (Agilent, CA, USA).

### Western blotting analysis

Cells were lysed with lysis buffer containing 25 mM Tris pH7.6, 150 mM NaCl, 1% sodium deoxycholate, 1% Triton-X100, and 0.1%SDS. The protein samples were quantified using a BCA protein assay kit (Thermo Fisher, CA, USA). Protein samples were run on and separated by SDS-PAGE and proteins were transferred onto the PVDF membrane followed by blocking with 5% non-fat milk for one hour at the bench top. The PVDF membranes were incubated with different specific primary antibodies overnight at 4℃ followed by hybridizing with the corresponding secondary antibodies. ECL substrate was applied onto the membrane and the chemiluminescence signals were detected and quantified using the ChemiDoc imaging system (Biorad, CA, USA). The detailed information of antibodies was listed in Additional file 1: Table S1). The original western blotting images are provided in Additional file 3.

### Immunofluorescence staining and confocal imaging

Cells were grown on fibronectin-coated coverslips in the 24-well plates and were incubated overnight. Cells were fixed with 4% paraformaldehyde for 5 min followed by permeabilization with 0.5% Triton X-100 for 5 min. Next, cells were blocked in 5% goat serum for 1 h. Cells were then stained with the specific antibodies at 4 ℃ overnight followed by staining with fluorescence-tagged secondary antibodies. Cells were then washed with 1 × PBS and mounted in Prolong Antifade reagent (Invitrogen, CA, USA). The fluorescence images of cells were visualized and photographed using the Leica SP5 II scanning confocal microscope with 60 × objective HCX PLAPO (NA = 1.25, oil immersion; Leica).

### Quantification of phosphorylated p65 nuclear translocation

The immunofluorescence staining was performed on TNF-α-treated cells using an anti-phospho-p65 antibody. The quantification of nuclear phospho-p65 positive cells was carried out by analyzing the count of Alexa 488-positive nuclei per 100 × magnified field under a fluorescence microscope using Image J software.

### Expression and purification of GST-tagged SCEL recombinant protein

The full length of human SCEL cDNA was cloned into pGEX-4T-2 expression vector using SmaI and NotI restriction enzyme sites to obtain the GST-tagged SCEL expression construct pGEX-4T-2-SCEL. *E. coli* BL21 (DE3) strain (ThermoFisher Scientific Inc., CA, USA) was transformed with pGEX-4T-2-SCEL and was grown in LB medium containing 50 μg/mL ampicillin to reach an optical density (OD600) of 0.5 followed by induction with 0.1 mM isopropyl-β-d-thiogalactopyronoside (IPTG) for 4 h at 20 ℃. Samples were harvested by centrifugation at 10,000 rpm for 10 min. Subsequently, the culture supernatant was mixed with binding buffer (140 mM NaCl, 2.7 mM KCl, 10 mM Na_2_HPO_4_, 1.8 mM KH_2_PO_4_, pH 7.4), and was injected into a GSTrap™ FF column (Cytiva, CA, USA) with 1 mL of GSTrap resin (Cytiva, CA, USA) at a flow rate of 0.5 mL/min. The packed column was washed with a 20-fold volume of binding buffer, and the bound protein was eluted with elution buffer (50 mM Tris–HCl, 10 mM reduced glutathione, pH 8.0) at 1 mL/min followed by filtration with microcon 100 kDa (Merck, Darmstadt, Germany). The concentration of the eluted protein was determined using BCA protein assay kit (Pierce, IL, USA). The purity of the GST-SCEL recombinant protein was analyzed by 10% SDS-PAGE.

### GST pull-down assay

1 × 10^7^ control cells and His-tagged TNFR1-expressing cells were grown in the 10-cm culture dish and harvested in 500 μL lysis buffer (25 mM Tris–HCl, 150 mM NaCl, 10% glycerol, 1% NP40), respectively. 5 μg of the purified GST-fused SCEL protein was incubated with Glutathione Sepharose 4B for 2 h at 4 ℃ followed by centrifugation at 500×*g* for 5 min. GST-SCEL beads were washed three times with 10 bed volume of ice cold 1 × PBS. 50 μL of GST-SCEL beads was added in 500 μL control lysate or His-tagged TNFR1-expressing cell lysate, respectively, and incubated at 4 ℃ overnight. The beads were centrifuged at 500×*g* for 5 min followed by wash with lysis buffer three times. Lastly, 2 × SDS sample buffer was added to the beads, and subjected to western blotting analysis to detect the interaction between GST-SCEL and Hig-tagged TNFR1.

### Protein degradation assay

5 × 10^5^ cells were seeded in the 6-well plate 24 h prior to the cycloheximide (CHX) treatment. Cells were then incubated in the presence of 20 μg/mL CHX and harvested in the lysis buffer at the indicated time points for western blotting analysis.

### Co-immunoprecipitation

LC cells were transfected with HA-SCEL expression vector pCMV-HA-SCEL for 24 h and grown to confluency in the 10-cm culture prior to the protein extraction. The cells were lysed with co-IP buffer (25 mM Tris–HCl pH 7.4, 150 mM NaCl, 1% NP-40, 2 mM EDTA) followed by protein quantification. 1 mg of total protein was incubated with 2 μg control IgG antibody (#3900, Cell signaling, CA, USA), anti-TNFR1 antibody (#3736, Cell signaling, CA, USA), and anti-HA antibody (#3724, Sigma-Aldrich, CA, USA), respectively, at 4 ℃ on the rotator overnight. 100 μL protein A magnetic sepharose (cytiva, CA, USA) slurry was then added to the protein-antibody mixture followed by a 1-h incubation at 4 ℃ on the rotator. The samples were spun at 3,000 rpm for 5 min and the protein A magnetic sepharose was washed with co-IP buffer twice to remove the non-specific binding of proteins on the beads. Lastly, 2 × sample buffer (100 mM Tris–HCl pH 6.8, 0.1% bromophenol blue, 200 mM dithiothreitol, 20% glycerol, 20% SDS) was added to each sample to elute the proteins captured by the antibodies followed by western blotting analysis.

### Anti-TNF-α therapy in an experimental lung colonization mouse model

250,000 luciferase-tagged LC cells and stable SCEL knockdown LC cells were i.v. injected into the SCID mice 24 h prior to the anti-TNF-α treatment, respectively. Mice were treated with 200 μg (Humira^®^, AbbVie Inc., SW) or control IgG via tail vein injection every week for a total of 4 injections over the course of one month. On week five, 200 μL 2 mg/mL luciferin substrate was administered into mice via tail vein followed by sacrifice. The mouse lungs were then dissected and analyzed for the presence of metastatic cells by the IVIS^®^ Lumina LT series III imaging system (PerkinElmer Inc., CA, USA). The bioluminescence signal of the mouse lung was recorded and quantified using IVIS software (PerkinElmer Inc., CA, USA).

### iTRAQ-based liquid chromatography coupled with tandem mass spectrometry (LC–MS/MS)

The protein extracts from different subcellular compartments including the membrane, cytoplasmic, and nucleus fractions were digested with sequencing grade trypsin (Promega, Madison, WI, USA) by using the filter-aided sample preparation method [24]. The digested proteins were then acidified and desalted using Sep-Pak C18 cartridges to remove impurities. (Waters Associates, Milford Mass, USA). The purified peptides were labeled with different channels of isobaric tags using iTRAQ-4-plex reagents (Applied Biosystems, Foster City, CA, USA) according to the manufacturer’s instructions. The labeled peptides with different channels of isobaric tags were pooled and then desalted by SepPak C18 cartridges. The labeled peptides were then pooled, desalted, and dried under vacuum before being re-suspended in 0.5% trifluoroacetic acid for further fractionation using a high pH fractionation system (Pierce High pH Reversed-Phase Peptide Fractionation Kit, Thermo Fisher Scientific, USA) as manufacturer’s recommendation. This resulted in ten fractions of bound peptides, which were eluted in a 1% ammonia solution with varying amounts of acetonitrile. These eluted fractions were dried under vacuum and re-suspended in 0.1% formic acid solution for the liquid chromatography coupled with tandem mass spectrometry (LC–MS/MS) analysis**.** Tandem mass spectrometry-based protein identification was performed on Q Exactive™ HF mass spectrometer (Thermo Fisher, San Jose, USA) coupled with a Thermo Scientific™ UltiMate™ 3000 RSLCnano HPLC System. The detailed parameters were described in the Additional file 1.

### Analysis of MS-generated proteomic data by Proteome Discoverer software and Mascot search engine

The MS raw files were uploaded into Proteome Discoverer (version 2.1, Thermo Fisher Scientific, MA, USA) and were used to match and identify proteins using the MASCOT search engine (version 2.5, Matrix Science, MA, USA). The detailed parameters were described in the Additional file 1. The iTRAQ Result files including the full list of iTRAQ-generated LC membrane list (Additional file 1: Table S3) and iTRAQ-generated IV2 membrane list (Additional file 1: Table S4) are provided.

### Public domain datasets and clinical samples

The clinical association of SCEL was investigated in the TCGA breast cancer (BCRA) dataset using UCSC Xena online platform (www.ucscxena.com). In addition, a total of 48 GSE datasets were analyzed using KM plotter online platform (www.kmplot.com). The following GSE datasets were analyzed: GSE11121, GSE12093, GSE12276, GSE1456, GSE16391, GSE16446, GSE16716, GSE17705, GSE17907, GSE18728, GSE19615, GSE20194, GSE20271, GSE2034, GSE20685, GSE20711, GSE21653, GSE22093, GSE25066, GSE2603, GSE26971, GSE29044, GSE2790, GSE31448, GSE31519, GSE32046, GSE3494, GSE36771, GSE37946, GSE41998, GSE42568, GSE43358, GSE43365, GSE45255, GSE4611, GSE46184, GSE48390, GSE50948, GSE5327, GSE58812, GSE61304, GSE65194, GSE6532, GSE690321, GSE7390, GSE76275, GSE78958, and GSE9195. Additionally, TNBC samples were extracted from GSE datasets based on PAM50 for survival analysis. The cut-off value of SCEL was determined using the function of auto select best cutoff [20]. For the analysis of SCEL protein expression in clinical TNBC tumor samples, TNBC tissue microarrays were purchased from US Biomax, Inc. (CA, USA) (Additional file 1: Table S5).

### Quantitative real-time polymerase chain reaction (qRT-PCR)

The comprehensive method has been previously documented elsewhere [19]. The specific primer sequences are provided in Additional file 1: Table S6.

### Lentivirus shRNA knockdown

The detailed procedure has been described elsewhere [19]. The SCEL shRNA sequences are listed in Additional file 1: Table S6.

### Statistical analysis

The student t-test was utilized to analyze differences between two datasets. The Log-rank test was applied to compare the Kaplan–Meier survival curves of two patient groups, and the Cox proportional hazards model was used to estimate the hazard ratio between the groups. For comparisons between more than two groups, the one-way ANOVA was used to assess differences in means. Pearson’s Chi-squared test was employed to evaluate deviations between observed and expected values within specific groups. Spearman's rank correlation coefficient (also known as Spearman's rho) was used to assess the correlation of protein expressions in clinical samples. In all tests, statistical significance was defined as a p value of less than 0.05.

## Results

### iTRAQ LC–MS/MS identified a unique membrane-associated protein profile in the LC cells derived from the long-term slow growing metastatic lung nodules of TNBC

**Fig. 1:**
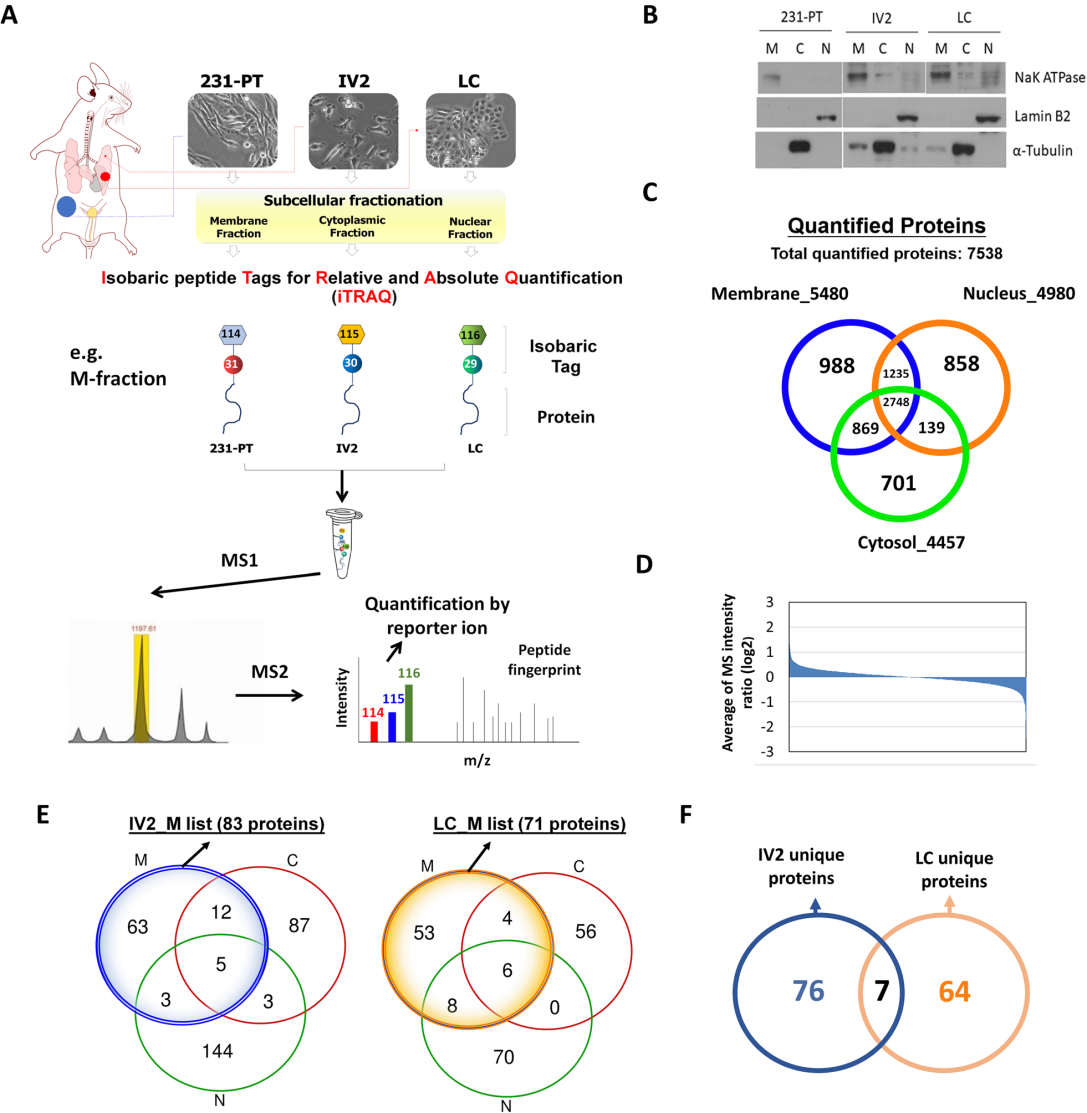
iTRAQ-based LC MS/MS approach identifies a unique membrane-related protein profiles in the metastatic TNBC cells derived from the metastatic lung nodules.** A** A schematic of identification of metastasis-related membrane proteins using iTRAQ-coupled LC MS/MS. **B** Subcellular protein fractionation of 231-PT, IV2 and LC cells. **C** MS-generated proteomic data were quantified and identified by proteome discoverer software and Mascot search engine, respectively. A total of 7538 proteins from three cellular compartments of 231-PT, IV2, LC cells was successfully identified and quantified. **D** Average of MS intensity ratio (log2) of IV2 versus 231-PT and LC versus 231-PT. **E** Venn diagram analysis of the proteins with 1.5-fold increase from three compartments. The metastasis-related proteins that were upregulated at least 1.5-fold in the membrane fraction of IV2 cells (shown as the blue circle) and LC cells (shown as the orange circle) as compared to that of the 231-PT cells were identified. **F** Venn diagram analysis of IV2-specific and LC-specific membrane-associated protein

In order to identify proteins that are essential for TNBC metastatic colonization, we applied a quantitative proteomic approach iTRAQ LC–MS/MS technique to systematically compare the protein expression profiles among MDA-MB-231-PT and MDA-MB-231-derived IV2 and LC cells as illustrated in Fig. 1A. As described previously, 231-PT, IV2 and LC cells were isolated from the 231-derived primary tumors, early lung metastases, and long-term slow growing lung metastases, respectively, of the orthotopic breast cancer mouse model [18]. The cellular proteins of 231-PT, IV2 and LC cells were fractionated into three compartments, membrane, cytosol and nucleus followed by iTRAQ-based LC–MS/MS proteomic analysis of peptides from the three compartments (Fig. 1B). A total of 7538 proteins were successfully quantified from three compartments and Venn diagram analysis showed that, among the identified proteins, 988 were membrane-associated proteins, 701 were cytosol-associated proteins, and 858 were nucleus-associated proteins (Fig. 1C). The averaging MS intensity ratio (log2) of the identified proteins were shown in Fig. 1D. We identified 71 and 83 membrane-associated proteins that were upregulated at least 1.5-fold in the LC cells IV2 cells, respectively, as compared to the 231-PT cells (Fig. 1E; Additional file 1: Tables S1, S2); By applying a supervised cluster analysis to the LC/IV2-enriched proteome in TCGA breast cancer dataset using the UCSC Xena platform, we identified a group of candidates that were exclusively upregulated in TNBC (Additional file 1: Fig. S1). Moreover, Venn diagram analysis revealed that 64 membrane-associated genes were uniquely expressed in LC cells, while 76 membrane-associated genes were exclusively expressed in IV2 cells, and 7 common genes were identified in both IV2 and LC cells (Fig. 1F; Additional file 1: Tables S1, S2).

Subsequently, we selected 64 genes that were uniquely expressed in LC cells, which could be relevant to the lung colonization potential of TNBC cells, for further clinical association studies. 

### High expression of Sciellin (SCEL) is significantly associated with TNBC and the poor survival of BC patients

**Fig. 2:**
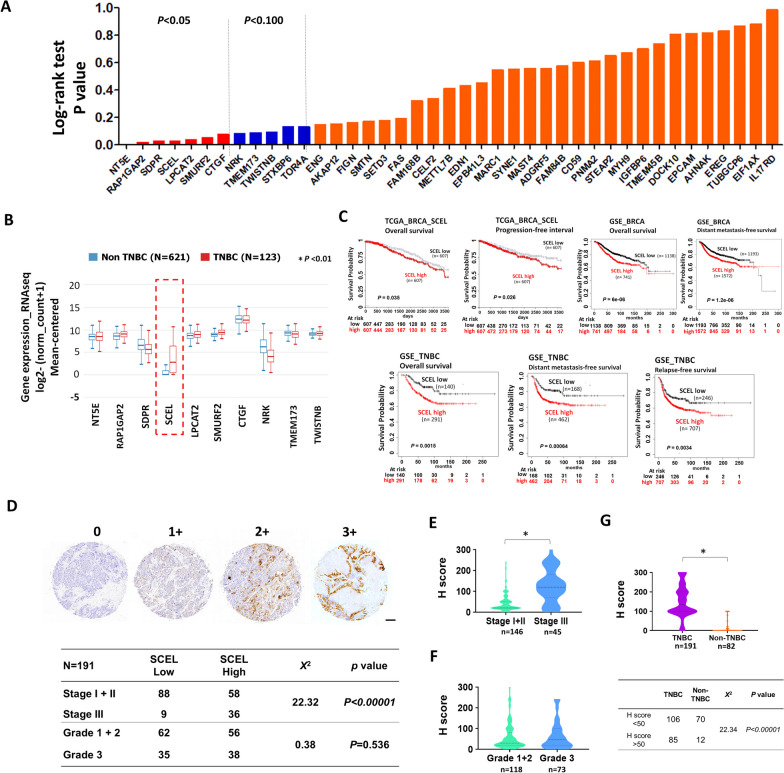
SCEL expression is significantly upregulated in TNBC tumors and correlates with the poor outcome of patients with TNBC. **A** Analysis of the survival correlation of the candidate proteins in TCGA breast cancer dataset using UCSC Xena platform. **B** Analysis of mRNA expression levels of top seven candidates, NT5E, RAP1GAP2, SDPR, SCEL, LPCAT2, SMURF2, and CTGF, in TNBC tumors versus non-TNBC tumors using UCSC Xena. *P < 0.01. **C** The Kaplan–Meier survival analysis of BC patients and TNBC patients based on SCEL expression. The Kaplan–Meier survival curves of overall survival (OS) and progression-free survival (PFS) of breast cancer patients stratified by SCEL expression were generated by UCSC Xena using TCGA breast cancer dataset. The Kaplan–Meier survival curves of overall survival (OS) and distant metastasis-free survival (DMFS) of breast cancer patients stratified by SCEL expression were generated by KM plotter using 48 GSE datasets. The Kaplan–Meier survival curves of overall survival (OS), distant metastasis-free survival (DMFS), and relapse-free survival (RFS) of TNBC patients stratified by SCEL expression were similarly generated by KM plotter using 48 GSE datasets. **D** Immunohistochemistry (IHC) staining of SCEL protein expression in TNBC tissue microarrays (n = 191). Pearson Chi’s square test of the observed values and the expected values of the early/late-stage tumors with low and high SCEL expression (*Χ*^2^ = 22.32, *P* < 0.00001). Pearson Chi’s square test was similarly performed in the lower and higher-grade tumors with low and high SCEL expression (*Χ*^2^ = 0.38, *P* < 0.536). **E** H score of SCEL IHC staining of the early-stage and late-stage TNBC tumors. *P < 0.001. **F** H score of SCEL IHC staining of the lower and higher grade TNBC tumors. **G** H scores of SCEL IHC staining of TNBC (n = 191) and non-TNBC tumors (n = 82). *P < 0.001

Next, we analyzed the clinical association of the LC cell-specific membrane-associated proteins. First, the correlation between the candidate proteins and the overall survival of BC patients was analyzed by UCSC Xena. The P-values of log-rank test of the Kaplan–Meier survival analysis were retrieved from TCGA database using UCSC Xena. As shown in Fig. 2A, high expression of NT5E, RAP1GAP2, SDPR, SCEL, LPCAT2, SMURF2, and CTGF were significantly associated with the poor survival of BC patients among the candidate proteins (Fig. 2A). Among these 7 candidates, the expression of SCEL was highly associated with the TNBC subtype (Fig. 2B). In addition, the Kaplan–Meier survival curve analysis showed that high mRNA expression of SCEL was significantly correlated with poor overall survival (OS) (*P* = 0.035) and shorter progression-free survival (PFS) (*P* = 0.026) of BC patients in TCGA BRCA dataset (Fig. 2C). Similarly, high expression of SCEL was also associated with poor OS (*P* < 0.001, hazard ratio (HR): 1.54, confidence interval (CI):1.27–1.86) and shorter distant metastasis-free survival (DMFS) (*P* < 0.001, HR:1.48, CI:1.26–1.76) of BC in GSE datasets (Fig. 2C). Further analysis of the correlation between SCEL expression and the outcomes of TNBC patients using GSE datasets revealed that high expression of SCEL was also significantly associated with poor OS (*P* = 0.0015, HR:2.13, CI 1.32–3.44), shorter DMFS (*P* < 0.001, HR:1.93, CI 1.31–2.82), and shorter relapse-free survival (RFS) of patients with TNBC (*P* < 0.0034, HR:1.48, CI 1.14–1.94) (Fig. 2C). Next, we examined the protein expression of SCEL in TNBC tumor specimens. Immunohistochemistry staining (IHC) analysis of TNBC tissue microarray showed that high expression of SCEL was significantly associated with the late-stage TNBC (Fig. 2D–F). The comparison of SCEL protein expression in TNBC and non-TNBC tumor tissues revealed a significant upregulation of SCEL expression in the TNBC tissues as compared to the non-TNBC tissues (Fig. 2G). In addition, the expression of SCEL was not found to be correlated with higher-grade or advanced stage of non-TNBC patients (Additional file 1: Fig. S2).

Altogether, we identified a novel metastasis-related protein SCEL with clinical implication and significance from the long-term slow growing metastatic lung nodules and it could play an important role during TNBC metastatic lung colonization.

### SCEL is a membrane-bound protein that is specifically expressed in the LC sublines derived from the long-term metastatic lung nodules

**Fig. 3:**
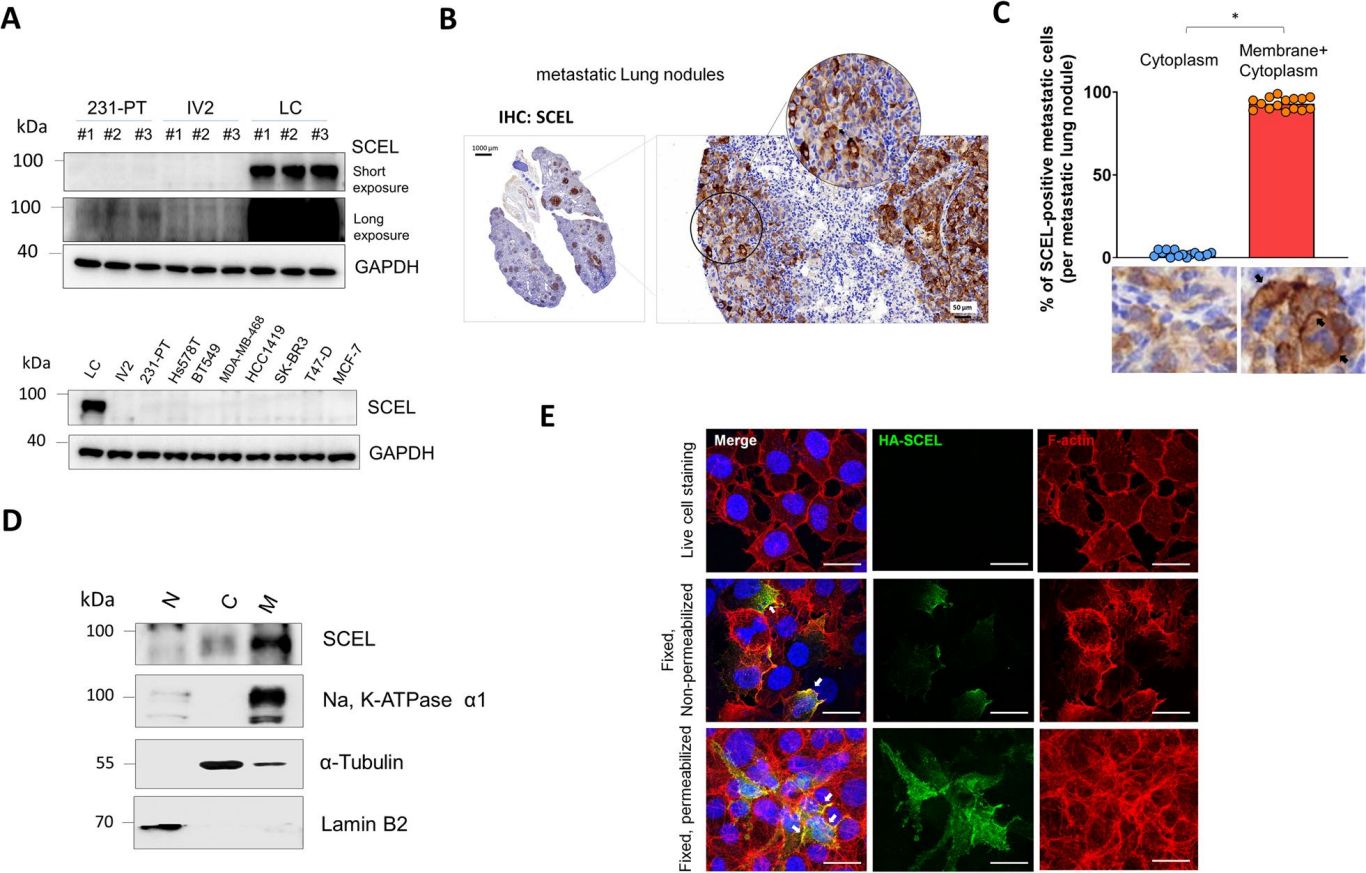
SCEL is a membrane associated protein that are exclusively expressed in the LC sublines derived from the metastatic lung nodules. **A** The protein expression of SCEL was compared in the multiple clones of MDA-MB-231-PT cells, and its sublines IV2, and LC cells isolated from the TNBC xenograft mouse model. Lower panel. The protein expression of SCEL was compared in a series of BC cell lines including luminal, HER2, and TNBC subtypes.** B** Immunohistochemistry staining of SCEL expression in LC cells-derived lung metastatic nodules in the tail vein injection mouse model. **C** Analysis of SCEL localization in LC cells-derived lung nodules using Image J software. Three nodules per mouse and a total of five mice were included in the analysis**.** Membrane staining of SCEL indicated by black arrows. **D** Subcellular localization analysis of SCEL in LC cells by protein fractionation. **E** The confocal immunofluorescence images of the HA-SCEL-transfected Hs578T cells. Membrane localization of SCEL indicated by white arrows. Scale bar, 25 μm. All cell-based experiments were performed in triplicates and repeated at least three times. The representative results are shown. *P < 0.01

Next, we examined the expression of SCEL in the LC sublines, 231-PT cells, and IV2 sublines (derived from short term lung metastatic nodules) [21]. Western blotting analysis demonstrated robust SCEL expression in LC sublines, whereas it was undetectable in both 231-PT and IV2 sub-lines under the short exposure condition (5 s). However, upon extended exposure (60 s), a minor amount of protein expression was observed in the 231-PT sublines (Fig. 3A). Moreover, investigation of SCEL expression in a series of breast cancer cell lines including luminal subtype (MCF-7 and T-47D), HER2 subtype (SK-BR-3 and HCC1419), and TNBC subtype (MDA-MB-468, BT-549, Hs578T), revealed that SCEL is specifically expressed in the long-term metastatic lung nodules-derived LC subline (Fig. 3A). In addition, we further analyzed the localization of SCEL protein expression in the mouse metastatic lung nodules. We found that SCEL expression was predominantly observed in both the cell membrane and cytoplasm of the majority of SCEL-positive metastatic cells (> 95%), with notably strong membrane staining (Fig. 3B, C); membrane staining indicated by the black arrows). Protein fractionation analysis further showed that SCEL was primarily enriched in the membrane fraction and less abundant in the cytoplasmic fraction (Fig. 3D). To examine the membrane localization of SCEL, an HA-SCEL fusion protein expression construct was introduced into the Hs578T cells for 24 h followed by immunofluorescent (IF) staining, and confocal imaging. As shown in Fig. 3E, three conditions were used to investigate the membrane localization of SCEL: live cell staining, cell fixation without permeabilization, and cell fixation with permeabilization. The results showed that no green fluorescence signals were detected from the HA-SCEL-transfected Hs578T cells under live cell staining condition (top, Fig. 3E). Under the fixation and non-permeabilization condition, we observed a green fluorescence staining at the peripheral membrane and within the cytoplasm of the transfected cells (middle, Fig. 3E). Under the permeabilization condition, we observed a more pronounced membrane and intracellular staining of SCEL in the transfected cells (bottom, Fig. 3E). These results suggested that SCEL protein was mainly localized to the inner part of the cell membrane and the cytoplasm. Taken together, our results confirmed that high expression of SCEL was specifically expressed in the LC cells derived from the long-term slow growing metastatic lung nodules. Additionally, the localization of the SCEL protein to the inner part of the cell membrane and cytosolic region may suggest the potential SCEL function associated with membrane receptors of LC cells.

### Downregulation of SCEL expression significantly suppresses anchorage-independent growth and lung colonization potential of lung-tropic LC cells, as well as decreased metastasis-caused death resulting from LC cell-derived metastatic tumors

**Fig. 4:**
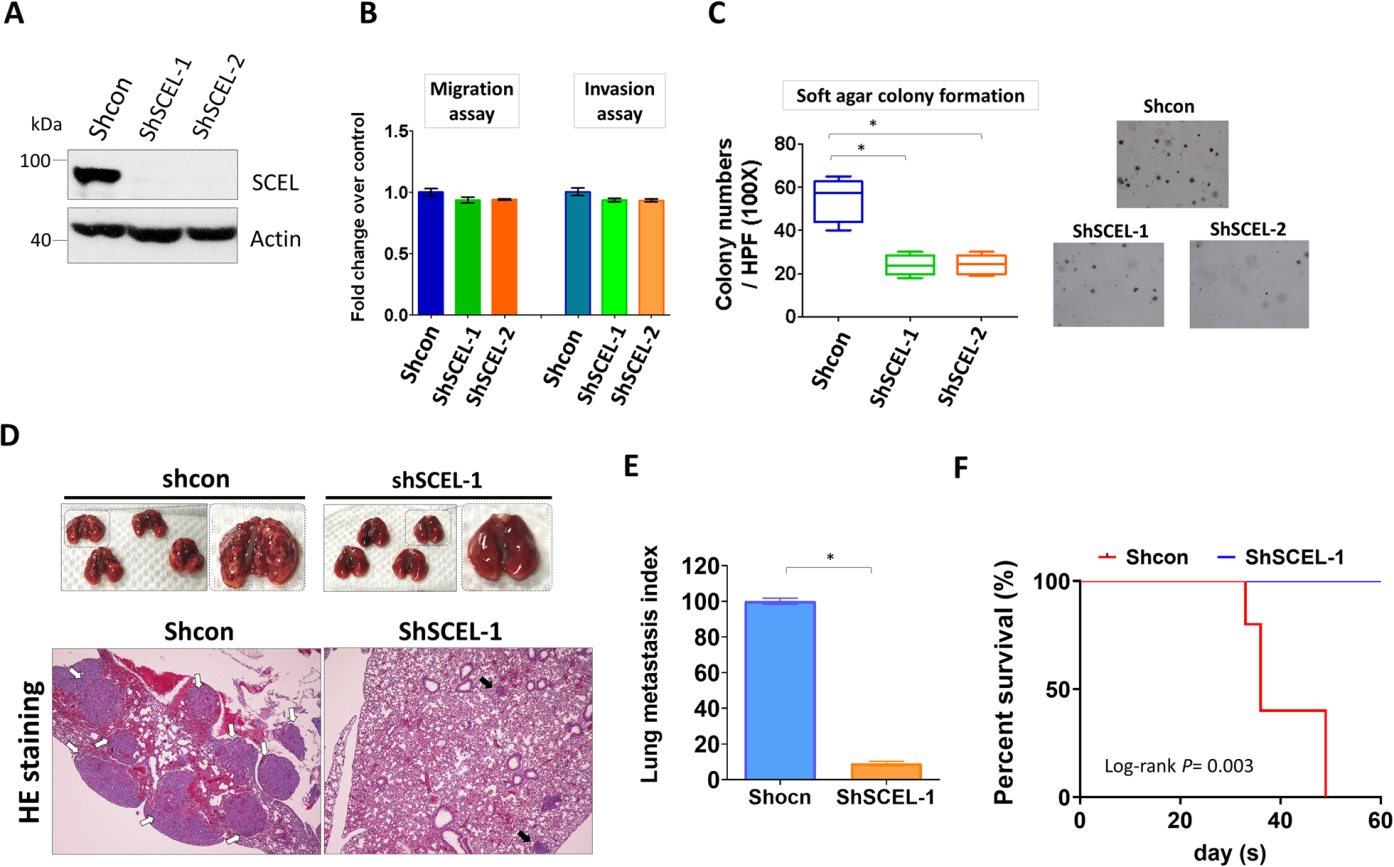
Knockdown of SCEL expression significantly reduces lung colonization potential of lung-tropic LC cells and prevents metastasis-caused death in a mouse model. **A** Western blot analysis of SCEL stable knockdown LC cells.** B** Transwell migration and invasion analysis of the control LC cells and two SCEL stable knockdown cells. **C** Analysis of anchorage-independent growth of SCEL-downregulated LC cell and the control LC cells using 3D-colony-forming assay. *P < 0.05.** D** Upper images showed the mouse lungs dissected from SCID mice intravenously injected with the control LC cells (n = 4) or SCEL-downregulated LC cells (n = 4). Bottom images showed HE staining of the mouse lung sections from the control group and the SCEL knockdown group. **E** Quantitative analysis of lung metastasis of mice intravenously injected with the control LC cells or SCEL-downregulated LC cells. *P < 0.001. **F** The Kaplan-Meir survival analysis of mice intravenously injected with the control LC cells or SCEL-downregulated LC cells. For the cell-based experiments, each experiment was performed in triplicates and was repeated at least 3 times

In order to address the possible role of SCEL in lung metastatic colonization of TNBC cells, we first investigated the effect of SCEL knockdown on the in vitro metastasis-related traits of cells including motility, invasiveness and 3D colony-forming ability. Western blotting confirmed the shRNA knockdown of SCEL in two stable SCEL knockdown LC lines (Fig. 4A). The Transwell migration and invasion assay showed that downregulation of SCEL expression did not significantly affect cell motility and invasiveness as compared to the control cells (Fig. 4B). On the other hand, the SCEL-downregulated cells showed an impaired ability for anchorage-independent growth when compared with the control cells (Fig. 4C). Based on these observations, we next examined whether SCEL downregulation could negatively affect the lung colonization potential of LC cells in a mouse model. The experimental lung metastasis mouse model was then performed by injecting the control or the SCEL-downregulated LC cells into the mouse tail vein and examined for lung metastasis one month later. As shown in the upper panel of Fig. 4D, the lungs resected from the mice injected with the control LC cells displayed many visible metastatic nodules, while the lungs from those injected with the SCEL-downregulated LC cells displayed relatively normal appearance. HE staining of the mouse lung sections revealed that LC cells effectively formed several sizable nodules in the mouse lung parenchyma (indicated by white arrows). In contrast, the SCEL-downregulated LC cells only formed a few minuscule metastatic lesions in the lungs (indicated by black arrows, Fig. 4D). Analysis of the area of lung parenchyma that was taken up by metastatic tumors showed that the SCEL-downregulated cells formed 90% fewer metastatic lesions than the control cells (Fig. 4E). Moreover, all mice injected with the SCEL-downregulated LC cells were able to survive through the experiment, while those injected with the control LC cells all died when reaching the endpoint (Fig. 4F). Taken together, our data indicated that SCEL played an essential role in lung metastatic colonization of TNBC cells but did not affect migration and invasion ability assayed in vitro. These observations suggest the effect of SCEL on lung metastasis may be subsequent to the arrival of cancer cells at lungs.

### Knockdown of SCEL expression significantly impairs TNF-α-induced NF-κB activation/nuclear translocation leading to the inhibition of downstream anti-apoptotic and proliferation genes

**Fig. 5:**
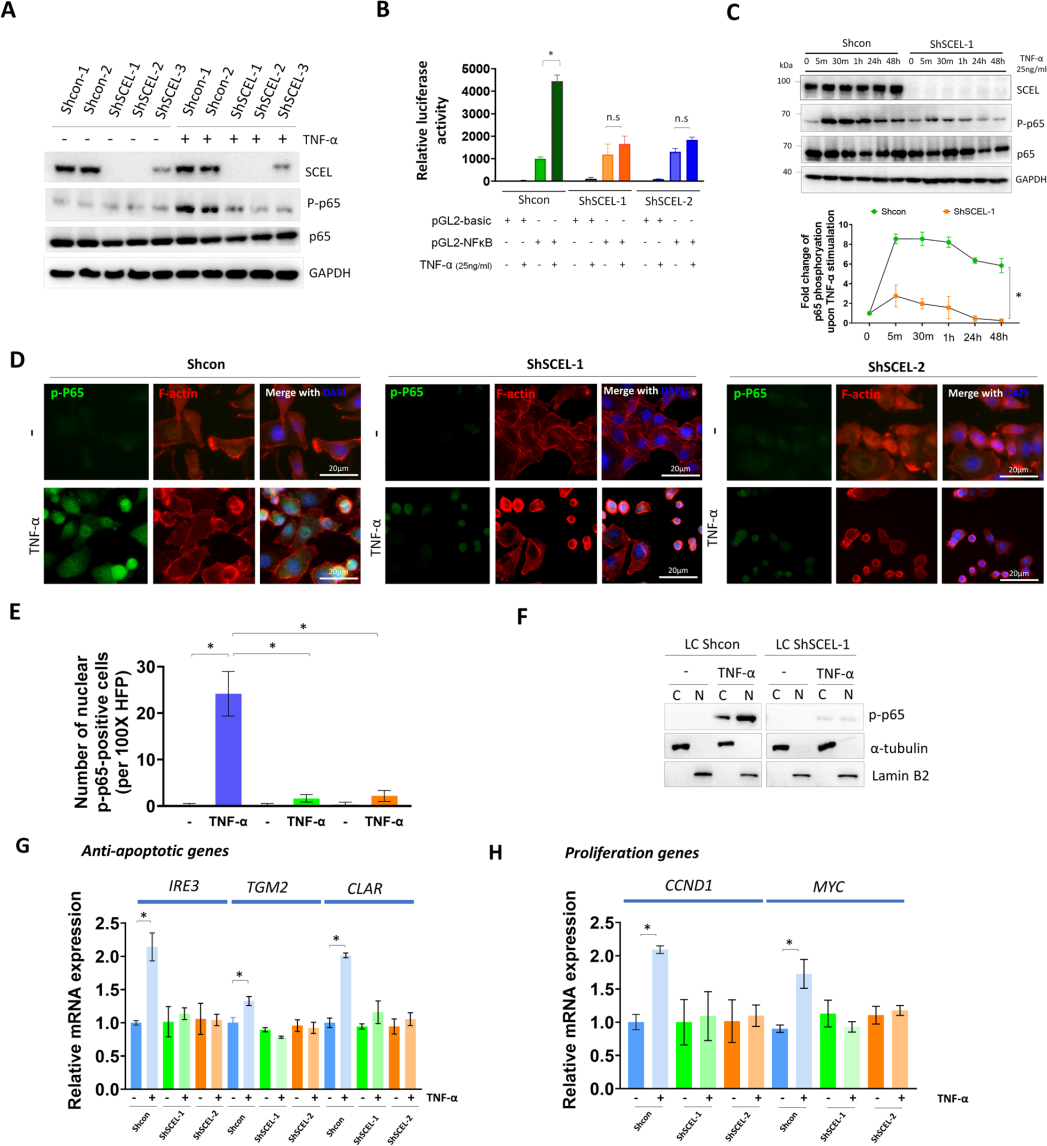
Knockdown of SCEL expression significantly decreases TNF-α-induced NF-κB activation and the expression of NF-κB-driven anti-apoptotic genes. **A** Western blotting analysis of p65 activation upon TNF-α stimulation in multiple SCEL stable knockdown LC clones and the control LC cells.** B** Analysis of NF-κB reporter assay using the SCEL stable knockdown LC cells and the control LC cells. *P < 0.001. **C** Analysis of TNF-α-dependent p65 activation kinetics in SCEL-downregulated LC cell and the control LC cells. *P < 0.01.** D** The representative IF images of TNF-α-induced p65 phosphorylation and nuclear translocation in the SCEL-downregulated LC cells and the control LC cells. Scale bar, 20 μm. **E** Quantitative result of phosphorylated p65 nuclear translocation. *P < 0.01**. F** Analysis of nuclear translocation of phosphorylated p65 in the control cells and the SCEL-downregulated cells upon TNF-α stimulation using subcellular fractionation. **G** qRT-PCR analysis of anti-apoptotic gene expression in response to TNF-α-stimulation in the control LC and SCEL-downregulated LC cells. **H** qRT-PCR analysis of proliferation-related gene expression in response to TNF-α-stimulation in the control LC and SCEL-downregulated LC cells. *P < 0.01. Each experiment was performed in triplicates and was repeated at least 3 times

Upon reaching distant organ such as the lungs, disseminated cancer cells immediately face the survival challenges exerted by the new environment including lack of cell–cell adherence and nutrient providing niche. Here, we focused on growth factors and cytokines known to be present in the lung parenchyma where fibroblasts and macrophages are the main source of growth factors such as IGF-1, EGF and HGF, as well as inflammatory cytokines such as TNF-α and IL-6. In particular, TNF-α secreted from proinflammatory macrophages can act as a double-edged sword in the context of metastasis [22, 23]. Therefore, we compared the status of the signaling pathways induced by those factors in SCEL-downregulated LC cells and control LC cells, and examined if SCEL depletion could cause the cells to respond differently to those environmental cues. As shown in Additional file 1: Fig. S3, depletion of SCEL did not affect IGF-1, EGF and HGF-mediated growth promoting signaling in the LC cells. Notably, we observed a dramatic reduction of p65 phosphorylation 30 min after TNF-α stimulation in the SCEL-downregulated LC cells as compared with the control, while depletion of SCEL did not affect IL-6-induced Stat3 oncogenic signaling (Additional file 1: Fig. S3). We further confirmed this finding with multiple stable SCEL knockdown cells to show that depletion of SCEL indeed attenuated TNF-α-induced p65 phosphorylation (Fig. 5A). Given the known dual role of TNF-α in regulating pro-survival and pro-apoptotic signals in cancer cells [24, 25], we further explored the effect of SCEL on the molecular signaling regulated by TNF-α in lung-tropic LC cells. To do so, we transfected the LC cells with the NF-κB luciferase reporter plasmids followed by TNF-α stimulation. The NF-κB reporter assay showed similar results that TNF-α treatment significantly increased the luciferase activity in the NF-κB reporter plasmid-transfected LC cells as compared to the SCEL-downregulated LC cells (Fig. 5B). Kinetic analysis of p65 phosphorylation revealed that the control LC cells showed strong p65 activation upon TNF-α stimulation at the early time point of 5 min, and the p65 activation persisted through 48 h (Fig. 5C). However, SCEL-downregulated LC cells showed impaired p65 activation upon TNF-α stimulation at the early time point, and the activation dwindled down prematurely one hour later (Fig. 5C). Given that p65 phosphorylation triggers its nuclear translocation and transcription activation function, we next examined if SCEL depletion suppressed the nuclear translocation of p65 upon TNF-α stimulation. The results of immunofluorescence staining showed that abundant nuclear translocation of the phosphorylated p65 was observed in the control LC cells treated with TNF-α. On the contrary, the fluorescent intensity of the phosphorylated p65 was significantly reduced in SCEL-downregulated LC cells upon TNF-α stimulation, and so was the nuclear translocation of the phosphorylated p65 (Fig. 5D, E). Additionally, subcellular fractionation experiment confirmed that depletion of SCEL significantly inhibited the nuclear translocation of phosphorylated p65 upon TNF stimulation (Fig. 5F). TNF-α-induced NF-κB nuclear translocation has been known to activate the downstream anti-apoptotic genes/proliferation genes to promote cancer cell survival and growth in various human cancers [26]. Therefore, we examined if depletion of SCEL could impair the expression of the TNF-α/NF-κB-driven anti-apoptotic and cell proliferation genes in response to TNF-α. Quantitative real-time PCR (qRT-PCR) analysis showed that the expression of the NF-κB-driven anti-apoptotic genes including *IER3* (1EX-1L), *TGM2* (TGM2), and *CFLAR* (c-FLIP) as well as the NF-κB-driven proliferation genes such as *CCND1* (Cyclin D1) and *MYC* (Myc) were found to be increased in TNF-α-treated LC cells. However, these genes did not show significant elevation in SCEL-downregulated LC cells treated with TNF-α (Fig. 5G, H), Taken together, our data showed that knockdown of SCEL could impair TNF-α/NF-κB-driven anti-apoptotic and proliferation signals in the lung-tropic TNBC cells.

### SCEL is required for regulating the switches between TNF-α-mediated cell survival and apoptosis in lung-tropic TNBC cells

**Fig. 6:**
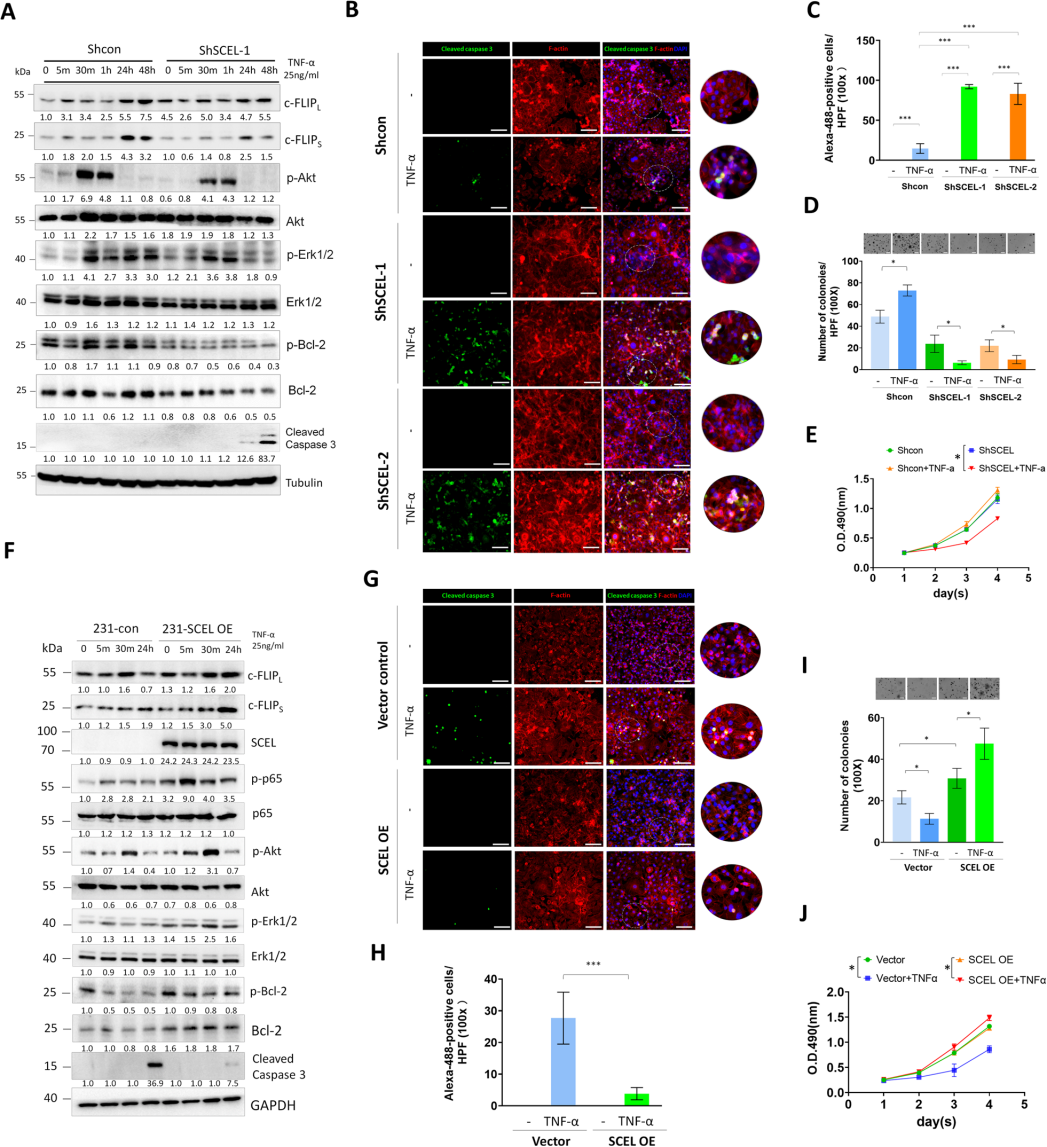
Loss of SCEL switches TNF-α-mediated cell survival to apoptosis in the lung-tropic metastatic TNBC cells. **A** Western blotting analysis of TNF-α-induced NF-κB-mediated pro-survival/apoptotic and proliferation signals in SCEL stable knockdown LC and the control LC cells.** B** The representative immunofluorescence (IF) images of cleaved caspase 3 in TNF-α-treated control LC and SCEL stable knockdown LC cells. Scale bar, 20 μm. **C** Quantitative result of the IF staining of TNF-α-induced cleaved caspase 3 in TNF-α-treated control LC and SCEL stable knockdown LC cells. *P < 0.01. **D** Evaluation of the capacity for anchorage-independent growth in control LC and SCEL-downregulated LC cells following TNF-α stimulation. *P < 0.05. **E** MTS cell proliferation analysis of the control LC and SCEL-downregulated LC cells in the presence or absence of TNF-α. *P < 0.05. **F** Western blotting analysis of TNF-α-induced NF-κB-mediated pro-survival/apoptotic and proliferation signals in SCEL-overexpressing 231 and the control 231 cells. **G** The representative immunofluorescence (IF) images of cleaved caspase 3 in TNF-α-treated SCEL-overexpressing 231 and the control 231 cells. Scale bar, 20 μm. **H** Quantitative result of the IF staining of TNF-α-induced cleaved caspase 3 in TNF-α-treated SCEL-overexpressing 231 and the control 231 cells. *P < 0.01. **I** Evaluation of the capacity for anchorage-independent growth in SCEL-overexpressing 231 cells and the control 231 following TNF-α stimulation. *P < 0.05. **J** MTS cell proliferation analysis of SCEL-overexpressing 231 and the control 231 cells in the presence or absence of TNF-α. *P < 0.05. Each experiment was performed at least three times

TNF-α binding to its receptor TNFR1 has pleiotropic effects that could trigger two distinct cellular signals: the pro-survival pathway, which is governed by the complex I (TNFR1/TRAF2) that activates NF-κB-driven anti-apoptotic pathway, and the pro-apoptotic pathway, which is regulated by the complex II (FADD/Caspase) that induces caspase signaling cascade [24, 26, 27]. Given that our data showed that SCEL depletion significantly impaired 3D colony-forming ability, suppressed in vivo lung colonization capability and blocked TNF-α**-**induced NF-κB pro-survival signals of LC cells, we inquired about whether SCEL depletion could switch TNF-α-induced cell survival to apoptosis in the TNBC cells. Thus, we examined the effect of SCEL depletion on the expression of the major effector of TNF-α/NF-κB-activated anti-apoptotic pathway, which is the anti-apoptotic protein, c-FLIP. Two isoforms, the long form, c-FLIP_L_, and the short form, c-FLIP_S_, are predominantly expressed in human, and both can function to inhibit caspase 8-mediated apoptosis cascade. The results of western blotting showed that the expression of c-FLIP_L_/c-FLIP_S_ was significantly elevated in LC cells after 24-h of TNF-α treatment as compared to SCEL knockdown LC cells (Fig. 6A), which confirmed the qRT-PCR result (Fig. 5F). Moreover, we found that TNF-α treatment significantly induced Akt phosphorylation at the 30-min time point, and downstream Erk1/2 activation was persisted up to 48 h in the control LC cells. Conversely, SCEL-downregulated cells exhibited impaired Akt phosphorylation upon TNF-α treatment as compared to the control LC cells (Fig. 6A). However, downstream Erk1/2 activation was unaffected during the early time points (30 min and 1 h), but its activation was reduced 24 h after TNF-α stimulation (Fig. 6A). Furthermore, we found that SCEL-downregulated cells exhibited diminished Bc1-2 phosphorylation and a decrease in the total Bcl-2 protein at the steady state level compared to the control cells (Fig. 6A). SCEL-downregulated cells showed a reduction in Bcl-2 at both phosphorylation and total protein levels during the 48-h TNF-α treatment, and displayed a significant increase in cleaved caspase 3 as compared to the control cells (Fig. 6A). In addition, the immunofluorescence (IF) staining confirmed a significant increase in cleaved caspase 3 in TNF-α-treated SCEL-downregulated LC cells as compared to TNF-α-treated control cells (Fig. 6B, C). Next, we examined if depletion of SCEL could affect cell growth in the soft agar culture and monolayer culture in response to TNF-α stimulus. The 3D colony-forming assay showed that TNF-α treatment promoted the colony-forming ability of the control LC cells. Conversely, TNF-α impaired the 3D colony-forming ability of SCEL-downregulated LC cells (Fig. 6D). The MTS cell proliferation assay showed that LC cells displayed an increased cell proliferation in response to TNF-α treatment (Fig. 6E), while TNF-α treatment significantly impaired the cell proliferation of the SCEL-downregulated LC cells (Fig. 6E).

Subsequently, we investigated whether overexpression of SCEL could enhance the TNF-α-induced anti-apoptotic and proliferation signals in the parental 231 cells. First, 231 cells were transduced with an SCEL-expressing lentiviral vector, followed by puromycin selection to establish stable SCEL-expressing 231 cells. We then examined the effect of SCEL overexpression on TNF-α-induced anti-apoptotic and proliferation signals. Overexpression expression of SCEL could significantly promote TNF-α-induced p65 and Akt/Erk1/2 phosphorylation and also maintained the level of Bcl-2 phosphorylation at the early timepoints of TNF-α treatment (Fig. 6F). Furthermore, overexpression of SCEL could significantly increase the protein levels of endogenous Bcl-2 (Fig. 6F). The SCEL-expressing 231 cells exhibited elevated cFLIP (c-FLIP_L_/c-FLIP_S_) protein expression after 24 h of TNF-α treatment as compared to the control 231 cells (Fig. 6F). Additionally, overexpression of SCEL effectively suppressed TNF-α-induced caspase 3 activation (Fig. 6G). The soft agar assay showed that the ectopic expression of SCEL significantly promoted the ability of anchorage-independent growth (AIG) of 231 cells (Fig. 6I). Notably SCEL-expressing cells exhibited the highest AIG capacity in response to TNF-α treatment. Conversely, the control cells displayed impaired AIG in the presence of TNF-α. In addition, TNF-α treatment elicited a modest promotion of cell proliferation in SCEL-expressing cells while inhibiting the growth of cells in a monolayer setting.

Collectively, our findings suggest that SCEL is required for regulating the switches between TNF-α-induced pro-survival and apoptosis, and it may play a crucial role in mediating the TNF-α-induced NF-κB/c-FLIP survival axis in TNBC cells.

### SCEL interacts with TNFR1 to promote its protein stability

**Fig. 7:**
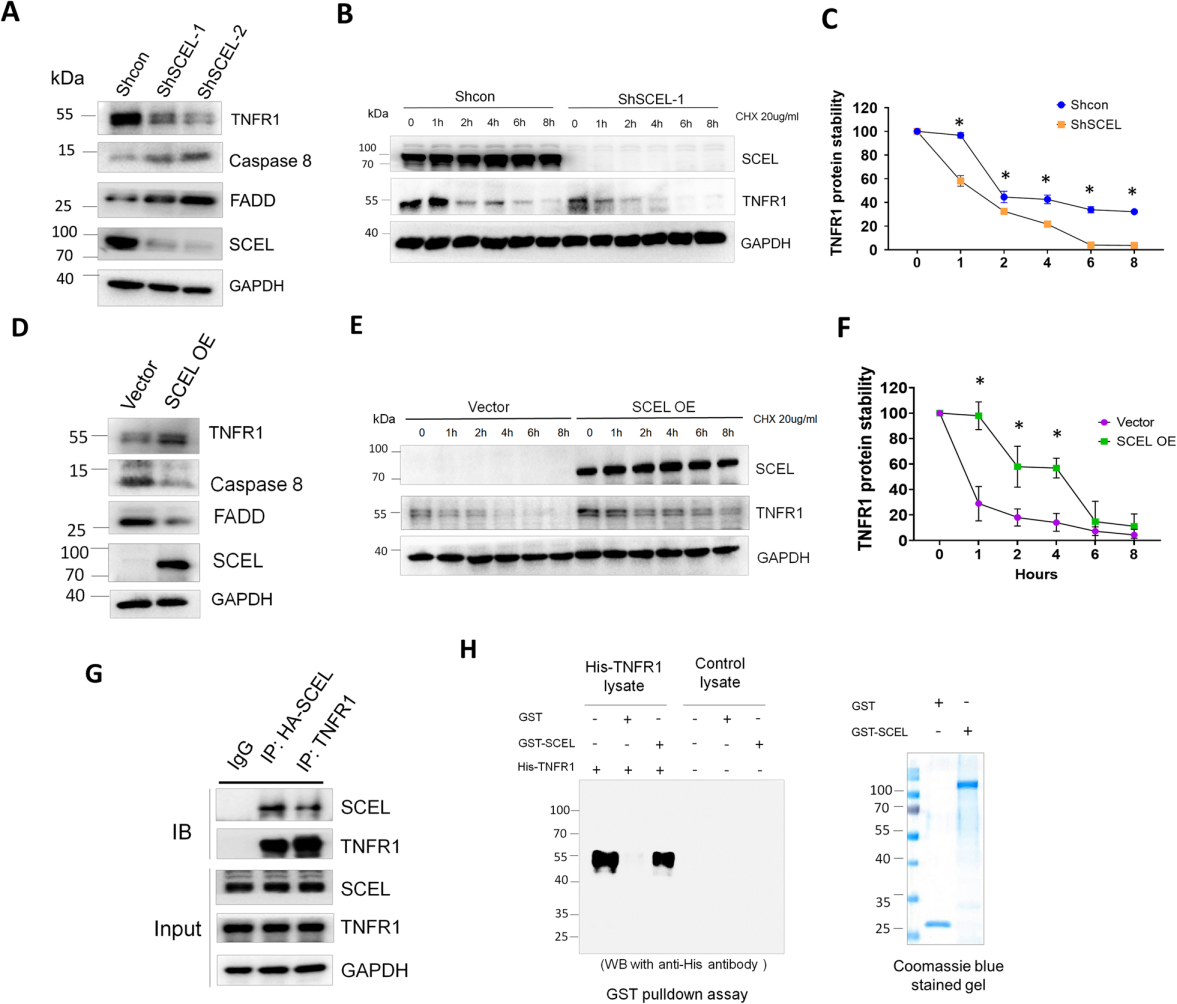
SCEL interacts with TNFR1 and plays a crucial role in maintaining its protein stability. **A** Western blotting analysis of protein expression of TNFR1 complex II in the SCEL stable knockdown LC cells and the control LC cells.** B** Protein degradation analysis of TNFR1 in the control LC cells and the SCEL-downregulated cells following 20 μg/mL cycloheximide (CHX) treatment. **C** Quantification of protein degradation kinetics of TNFR1 in the control LC and the SCEL-downregulated cells following 20 μg/mL cycloheximide (CHX) treatment. *P < 0.05. **D** Western blotting analysis of protein expression of TNFR1 complex II in SCEL-overexpressing 231 and the control 231 cells.** E** Protein degradation analysis of TNFR1 in in SCEL-overexpressing 231 and the control 231 cells in response to 20 μg/ml cycloheximide (CHX) treatment (top). Quantification of protein degradation of TNFR1 (bottom). *P < 0.01. **F** Analysis of protein interaction between SCEL and TNFR1 using reciprocal co-immunoprecipitation (co-IP) **G** Analysis of protein interaction between SCEL and TNFR1 using reciprocal co-immunoprecipitation (co-IP). **H** Examination of interaction between SCEL and TNFR1 through GST pull-down assay. Each experiment was repeated at least 3 times

Previous studies have shown that the membrane localization of TNFR1 is important for sustaining complex I/NF-κB pro-survival machinery, and TNFR1 protein instability triggers complex II-mediated apoptotic signals [24]. Thus, we examined whether depletion of SCEL in lung-tropic LC cells had an effect on the stability of TNFR1 protein and complex II protein such as FADD and caspase 8. First, we assessed if TNFR1 protein expression was altered in SCEL-downregulated LC cells. The result of western blotting revealed a reduction in TNFR1 protein levels, concomitant with elevated levels of complex II protein, FADD and caspase 8, in the SCEL-downregulated cells as compared with the control LC cells (Fig. 7A). Next, we investigated if SCEL depletion influenced TNFR1 protein stability. The control LC cells and SCEL-downregulated LC cells were treated with medium containing 20 μg/mL cycloheximide to block protein synthesis. Cells were harvested at the indicated time points and subjected to western blotting analysis. The results showed that TNFR1 protein stability significantly decreased in the SCEL-downregulated as compared to the control LC cells (Fig. 7B). TNFR1 protein degradation kinetics analysis revealed that TNFR1 in SCEL-downregulated cells degraded much faster than that in the control cells (Fig. 7C). In SCEL-downregulated cells, about 40% and nearly 100% of TNFR1 was degraded, respectively, after 1-h and 6-h of CHX treatment. On the other hand, in the control cells, less than 5% of TNFR1 was degraded after 1-h of CHX treatment, and 40% of TNFR1 was still present at the 8-h time point. Next, we examined whether overexpression of SCEL had effect on the stability of TNFR1 protein and the levels of TNFR1 complex II in the parental 231 cells, which have no SCEL expression. The result of western blotting showed that overexpression of SCEL increased the stability of TNFR1 and reduced the levels of TNFR1 complex II, FADD and caspase 8 (Fig. 7D). Meanwhile, SCEL-overexpressing 231 cells showed increased TNFR1 protein stability following CHX treatment as compared to the control 231 cells (Fig. 7E). Analysis of protein degradation kinetics of TNFR1 demonstrated that overexpression of SCEL significantly reduced the degradation rate of TNFR1 following CHX treatment (Fig. 7F).

These results led us to hypothesize the possibility that the presence of SCEL could stabilize TNFR1 through protein interaction. To test this idea, we then performed co-immunoprecipitation to test interaction between SCEL and TNFR1. To do so, we transfected HA-tagged SCEL into LC cells and 48 h later harvested the cells for protein extraction and analysis of co-immunoprecipitation (Co-IP) using control IgG, anti-HA antibody or anti-TNFR1, respectively. The Co-IP result showed that TNFR1 could be pulled down with HA-SCEL using anti-HA antibody, suggesting the potential interaction between TNFR1 and SCEL. The TNFR1-SCEL interaction was confirmed by the reciprocal co-IP using anti-TNFR1 antibody (Fig. 7G). Furthermore, GST pull-down assay was carried out to investigate the protein interaction between SCEL and TNFR1. To do so, the recombinant GST-SCEL fusion protein was prepared using *E.-coli* BL-21 strain (please refer to “Method” section) followed by purification. Next, His-tagged TNR1 expression construct was introduced into 293 T cells, and cell lysate was harvested for the in vitro protein binding by incubation with GST-SCEL fusion protein. Subsequently, protein complex was pulled down using Glutathione Sepharose beads. Western blotting analysis of GST pull-down assay indicated that His-tagged TNFR1 could be successfully detected in the GST-SCEL pull-down sample as compared to the control GST pull-down sample (Fig. 7H). Coomassie blue staining indicated equivalent loading amounts of the control GST and GST-SCEL fusion proteins (Fig. 7H).

Together, our results suggest that SCEL may interact with TNFR1 and the loss of SCEL could lead to instability of the TNFR1 protein, which could potentially explain why LC cells that lack SCEL are prone to undergo apoptosis in the presence of TNF-α.

### Depletion of SCEL significantly inhibits cell proliferation and induces apoptosis in the lung metastatic loci

**Fig. 8:**
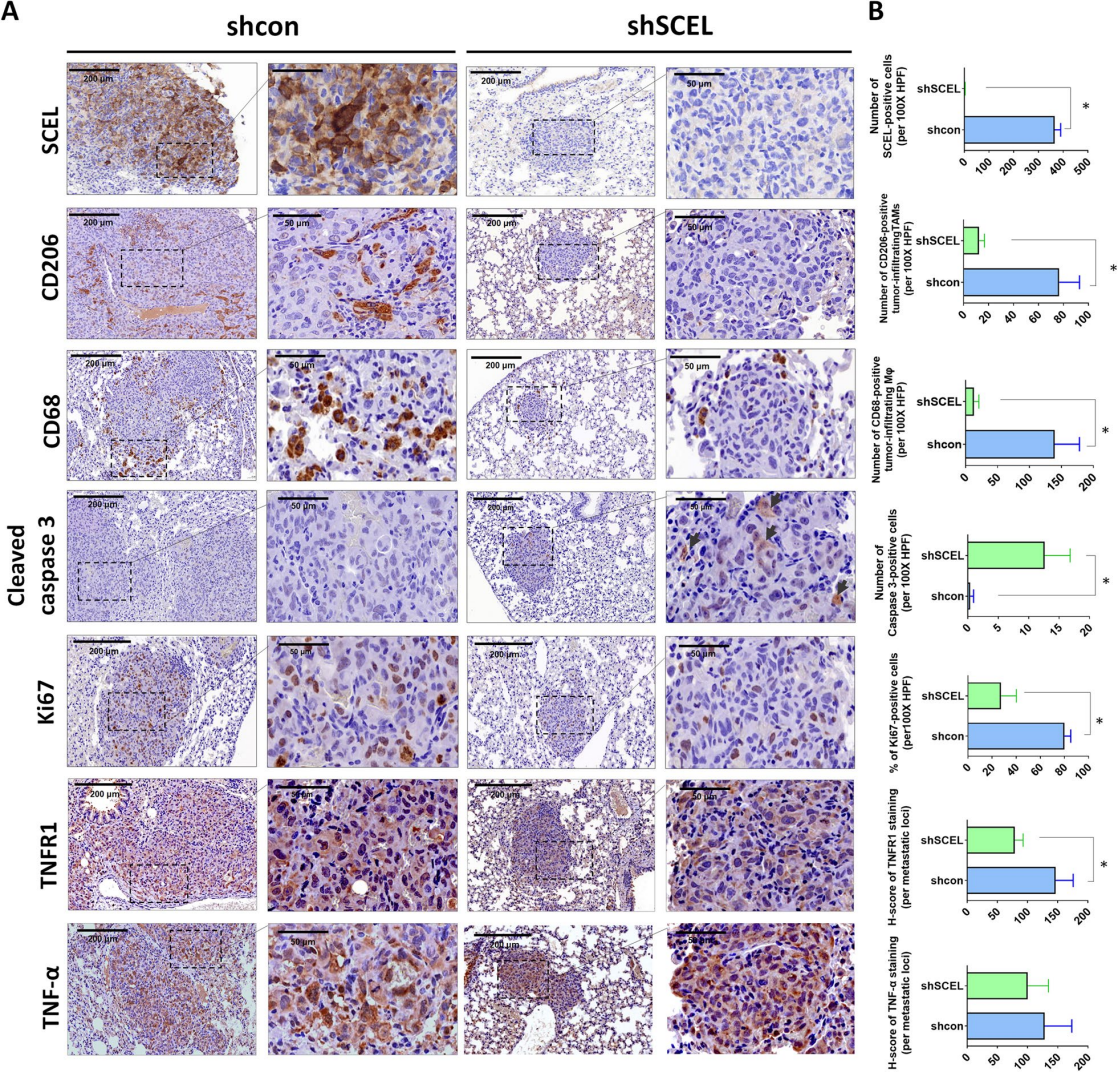
Depletion of SCEL suppresses cell proliferation and induces apoptosis in the lung metastatic loci. **A** Representative images of immunohistochemistry staining (IHC) for SCEL, CD206, CD68, cleaved caspase 3, Ki67 expression, TNFR1, and TNF-α of the metastatic lung nodules derived mice injected with the control LC and SCEL-downregulated LC cells, respectively. **B** Quantification of IHC staining of SCEL, CD206, CD68, cleaved caspase 3 and Ki67, TNFR1, and TNF-α expression in the metastatic lung nodules using Image J. *P < 0.05

Next, we examined whether the effect of SCEL depletion on the TNF-α mediated survival could be also observed in the metastatic loci of the mouse lung. To do so, we first performed IHC staining to observe the presence of tumor-associated macrophages (TAMs) including CD68^+^ M1 and CD206^+^ M2 macrophages in the lung metastatic loci. CD68^+^ M1 macrophages, known for its tumor-inhibiting ability, are the major TNF-α-producing immune cells in the tumor microenvironment [28]. The result of IHC staining showed that an increased number of CD68^+^ M1 macrophages were observed in the lung metastatic loci derived from the control LC cells as compared to that of in the lung metastatic loci derived from SCEL-downregulated LC cells (Fig. 8A, B). In addition, we also observe an increased number of CD206^+^ M2 macrophages, the tumor-promoting macrophages, in the lung metastatic loci of the control LC cells as compared to that of in SCEL-downregulated LC cells (Fig. 8A, B). These results indicated that depletion of SCEL in LC cells significantly reduced tumor-associated macrophage (TAMs) at the lung metastatic loci. Notably, elevated expression of cleaved caspase 3 and decreased expression of Ki67 were observed in the SCEL-downregulated LC cells-derived lung nodules as compared to those derived from the control LC cells (Fig. 8A, B). Furthermore, we demonstrated that shSCEL cells-derived lung nodules showed a decrease in TNFR1 expression as compared to the control cells-derived lung nodules whereas the levels of TNF-α were similar in both shSCEL and control nodules (Fig. 8A, B). Our in vivo evidence suggested SCEL expression is essential for the progression of metastatic lung colonization of TNBC through inhibiting apoptosis and promoting proliferation.

### The protein expression of SCEL significantly correlates with TNFR1 protein levels in TNBC specimens

Next, the clinical correlation between the protein expression levels of SCEL, TNF-α, and TNFR1 in TNBC tissues was investigated. IHC analysis demonstrated a significant correlation between the expression of SCEL and TNRF1. Pearson’s correlation coefficient analysis showed that elevated SCEL expression was significantly associated with increased TNFR1 levels, whereas there was no correlation between SCEL expression and TNF-α (Additional file 1: Fig. S4B). or between TNFR1 expression and TNF-α expression (Additional file 1: Fig. S4C). Together, IHC analysis of clinical TNBC tissues supports our cell-based and mechanistic study that SCEL expression is essential for maintaining the protein levels of TNFR1.

### TNF-α blockade therapy effectively suppresses the lung colonization potential of LC cells

**Fig. 9:**
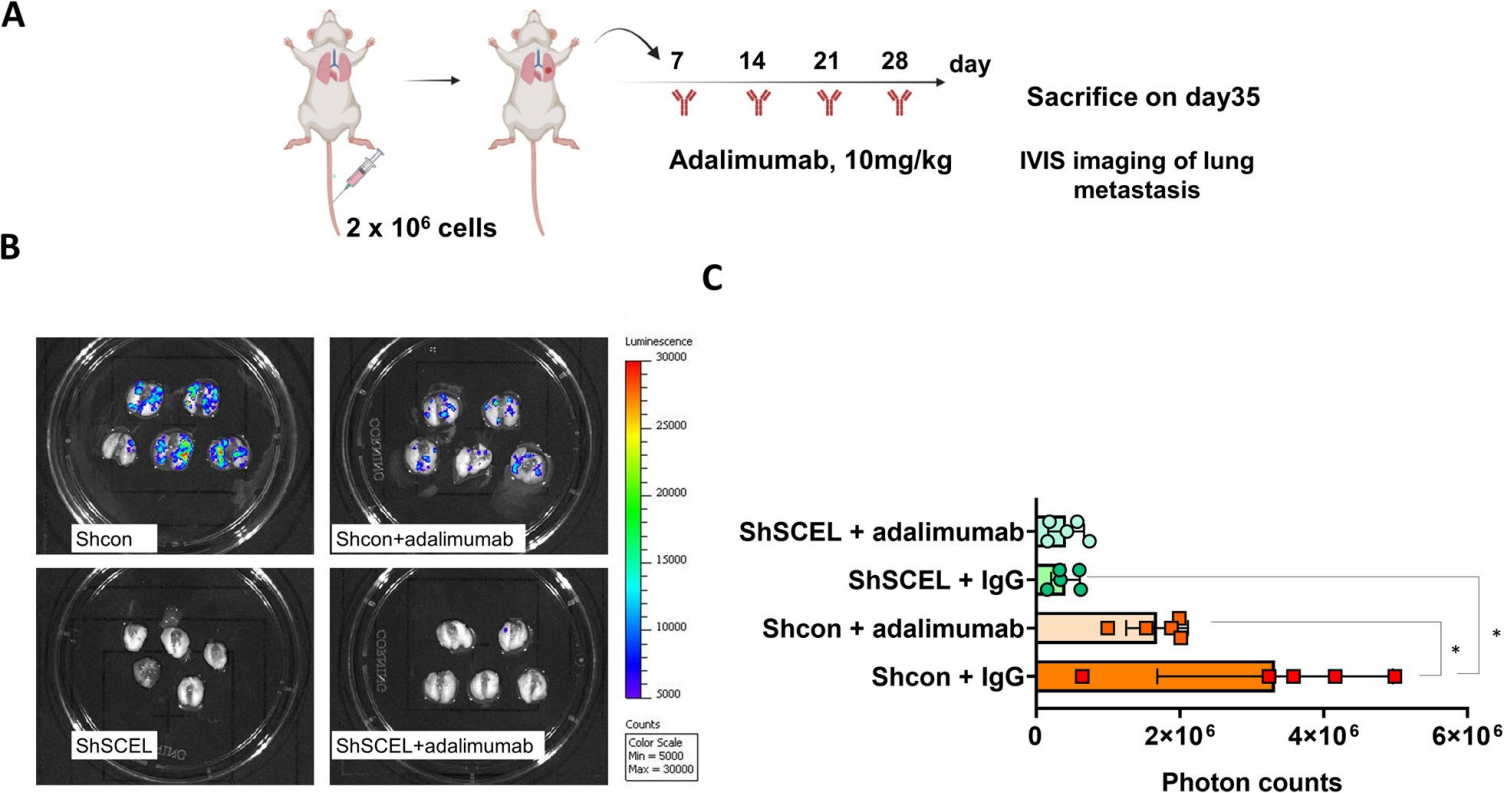
Administration of anti-TNF-α monoclonal antibody adalimumab effectively inhibits lung colonization potential of the lung-tropic LC cells. **A** Strategy of anti-TNF-α therapy in the experimental lung colonization mouse model. Tumor cells-injected mice were treated with weekly injection of adalimumab at a dosage of 10 mg/kg for a total of four injections. **B** Ex vivo analysis of chemiluminescence signals the mouse lungs using IVIS imaging Lumina LT series III imaging system. **C** Quantitative results of photon signals of the mouse lungs. *P < 0.05

Based on our above findings that SCEL could promote TNF-α-mediated NF-κB/c-FLIP survival signaling axis and depletion of SCEL effectively inhibited the formation of lung colonization of TNBC cells, we then proceed to explore the potential use of TNF-α blockade therapy to inhibit lung metastasis of TNBC. To do so, SCID mice were i.v. injected with the luciferase-tagged LC cells followed by weekly administration of 10 mg/kg of adalimumab (Humira^®^) or the control IgG for one month (Fig. 9A). Meanwhile, the SCEL-downregulated LC cells labeled with luciferase were similarly injected into mice via tail veins followed by adalimumab treatment (Fig. 9A). On week five, mice were sacrificed and the lungs were resected and treated with 2.5 mg/mL luciferin. The metastasis status was visualized and measured based on the luminescence signals using the IVIS imaging system. The IVIS results showed that adalimumab treatment significantly suppressed lung metastasis of mice injected with the control LC cells while it had no effect on those mice injected with the SCEL-downregulated cells (Fig. 9B, C). In addition, the IVIS experiment confirmed that SCEL depletion significantly inhibited the lung metastasis capability of the lung-tropic LC cells (Fig. 9B, C). Collectively, our data indicated that anti-TNF-α therapy could be a novel therapeutic approach for patients with metastatic TNBC and SCEL expression could be used as a predictive biomarker to stratify patients that could respond to the anti-TNF-α therapy.

## Discussion

In this study, we employed the iTRAQ-based proteomic approach to systemically analyze the membrane-associated protein profile in the lung-tropic LC cells and successfully identified a novel inner cell membrane-bound protein, SCEL, which could enhance TNF-α-stimulated NF-κB/c-FLIP survival axis and Akt/Erk1/2 growth signal, and at the same time, suppress caspase-3 activity through stabilizing TNFR1 protein. The mouse tail vein injection experiment confirmed that depletion of SCEL significantly impaired lung colonization capability of the LC cells. Depletion of SCEL expression significantly reduced lung metastatic lesions and prolonged the survival of mice. In addition, clinical link between SCEL and TNBC patients was established. High expression of SCEL was associated with advanced-stage TNBC and poor survival outcomes in TNBC, and was positively correlated with TNFR1 expression. Lastly, we demonstrated that blocking TNF-α-mediated NF-κB/Akt/c-FLIP survival axis by administration of adalimumab, an anti-TNF-α mAb drug, could effectively impair the lung colonization of the LC cells in the tail vein injection model.

TNBC is associated with a higher risk of recurrence and metastasis following treatment with curative intent compared to other subtypes of breast cancer. The distribution of metastasis varies by breast cancer subtypes. Patients with TNBC are at a greater risk of developing metastases in the lungs and brain compared to the other subtypes [29, 30]. Metastatic colonization, which is the initial growth of micrometastases, is the beginning of a fatal progression and is difficult to detect and treat at that stage [31]. Previous studies have shown that disseminated tumor cells (DTCs)-derived cellular factors could dictate organotropism of metastasis [31]. Oskarsson et al. showed that disseminated breast cancer cells could express tenascin C (TNC) to promote the survival and outgrowth of lung micrometastases by enhancing the expression of musashi homolog 1 (MSI1) and leucine-rich repeat-containing G protein-coupled receptor 5 (LGR5) to activate NOTCH (Neurogenic locus notch homolog protein 1) signaling and WNT pathway, respectively [32]. In addition, the co-evolution of DTCs and tissue-resident cells play a major part in fostering metastatic niche formation and survival of DTCs. The expression of periostin (POSTN) by resident pulmonary fibroblasts could alter the extracellular matrix to favor the initial lung colonization of infiltrating tumor cells through activating Wnt signaling in cancer stem cell [33, 34]. Additionally, Kitamura et al. showed that the survival and outgrowth of DTCs in the lung could be supported by CCL3 (CC-chemokine ligand 3) secreted from metastasis-associated macrophages (MAMs), which were recruited by DTCs-derived CCL2 (CC-chemokine ligand 2). The authors showed that MAMs, mainly originated from circulating inflammatory monocytes (IMs), could interact with metastatic cells and activation of the CCL3–CCR1 axis promotes adhesion of macrophages to VCAM-1 (Vascular cell adhesion protein 1) expressing cancer cells through integrin α4 [22]. Further, inhibition of CCL3-CCR1 (CC-chemokine receptor-1) axis using anti-CCL3 antibody or deletion of CCL3/CCR1 could effectively reduce breast cancer lung metastasis [22].

In addition, MAMs derived from circulating inflammatory monocytes and myeloid-derived suppressor cells (MDSCs) are the major inflammatory cytokines-producing immune cells at lung metastatic lesions. Although, MAMs-secreted TNF-α and IL-6 have been shown to support the progression of lung metastasis of TNBC cells, the role of TNF-α in regulating the metastatic growth of DTCs has been paradoxical that TNF-α can have both pro-survival and pro-apoptotic effects [23, 25]. Our data showed that metastatic LC cells managed to survive and proliferate in the lung parenchyma by upregulating SCEL expression to enhance TNF-α/TNFR1-mediated NF-κB /c-FLIP survival pathway and Akt/Erk1/2 growth signaling. Depletion of SCEL expression switched TNF-α/TNFR1-mediated cell survival to apoptosis in the lung-tropic LC cells (Fig. 6A–E). Conversely, ectopic expression of SCEL promoted TNF-α/TNFR1-mediated cell survival (Fig. 6F–J). In addition, our data showed that SCEL knockdown did not affect cell viability in monolayer culture. However, it did significantly affect the ability for anchorage-independent growth (Figs. 4C, 5G, 6E). Our reasoning was that the stable SCEL knockdown cells may have adapted to and compensated for the impact of TNFR1 instability over time in monolayer culture. Additionally, three-dimensional culture imposed a stressful growth condition that could induce apoptosis [35]. As a result, cells need to respond to this stress for survival and growth, and loss of SCEL significantly impaired their ability of cells to overcome such stress. Thus, SCEL may be a crucial stress-responsive protein that helps metastatic breast cancer cells overcome the unfavorable growth condition during early stage of lung colonization.

Ozes, et al. previously showed that activity of Akt is essential for TNF-α-induced IKK/NF-κB activation [36]. Our study confirmed this phenomenon and further suggested that SCEL may also play a role in Akt-mediated IKK/NF-κB activation (Fig. 6). TNFR1 protein stability has been shown to be essential for sustaining the complex I TNFR1/TRAF2-mediated pro-survival signaling upon TNF-α engagement [24, 27]. In this study, we provided evidence to show that SCEL could promote the protein stability of TNFR1 in TNBC cells. Depletion of SCEL significantly reduced the protein stability of TNFR1 (Fig. 7A–C). Conversely, ectopic expression of SCEL promoted the stability of TNFR1 protein (Fig. 7D–F). Our co-IP and GST pull-down assays revealed that SCEL had potential to interact with TNFR1, suggesting a direct molecular interaction between these two proteins. Our data indicate that the interaction between SCEL and TNFR1 is functionally relevant, as it may play an essential role for promoting TNFR1 stability upon TNF-α stimulation (Fig. 7G).

Furthermore, we provided in vivo evidence to show that depletion of SCEL abolished pro-metastatic phenotype of lung-tropic LC cells, as evidenced by an increased expression of cleaved caspase 3 and a reduced expression of Ki-67 in the lung metastatic loci of mice injected with SCEL-downregulated LC cells as compared to those injected with the control LC cells (Fig. 8A, B). Consistent with our mechanistic study, we observed a reduced level of TNFR1 in the lung metastatic loci derived from SCEL-downregulated cells (Fig. 8A, B). In addition, increased infiltration of CD206^+^ M2 macrophages at the metastatic site has been shown to favor the metastatic outgrowth of DTCs. On the other hand, proinflammatory CD68^+^ M1 macrophages are known to have antitumor effects, producing pro-inflammatory cytokines such as TNF-α to induce cancer cell apoptosis [28, 37]. Analysis of the tumor microenvironment of lung metastatic loci in mice injected with control LC cells revealed a substantial increase in MAMs, including CD68^+^ M1 and CD206^+^ M2 macrophages as compared to those injected with SCEL-downregulated LC cells. (Fig. 8A, B). These results suggested that SCEL-expressing LC cells may resist the attack from CD68^+^ M1 macrophages by taking advantage of the TNF-α/TNFR1/NF-κB/c-FLIP survival pathway and Akt-Erk1/2 proliferation axis to successfully form a macrometastasis. Based on our current data, we reasoned that when reaching to the lung parenchyma, the disseminated TNBC cells managed to increase their survival probability by upregulating SCEL expression to enhance TNF-α/TNFR1-mediated cell survival and proliferation upon arriving at distant metastatic sites such as the lung. Clinically, we demonstrated that the levels of SCEL protein were highly associated with TNFR1 expression in TNBC specimens (Additional file 1: Fig. S4). We found that high expression of SCEL was significantly associated with advanced-stage TNBC but not with higher grade TNBC (Fig. 2E, F), suggesting that SCEL expression may not contribute to the phenotype of poorly differentiated TNBC cells.

In order to translate our findings into the preclinical mouse model, we performed a TNF-α blockade therapy in an experimental lung colonization mouse model using the FDA-approved humanized TNF-α mAb, adalimumab, which can cross react with mouse TNF-α [38]. Our data indicated that administration of adalimumab significantly reduced metastatic lung colonization of SCEL-expressing LC cells while had no effect on the SCEL-downregulated LC cells (Fig. 9B, C). Previously, clinical trials of a TNF-α Inhibitor, Etanercept (Enbrel), in patients with metastatic breast cancer, showed that NF-κB activation and increased expression of TNF-α were associated with increments in docetaxel dose. Antitumor activity was noticed exclusively in the patient arm receiving etanercept [39, 40]. Kato et al. indicated that the use of the chimeric anti-TNF-α mAb, infliximab inhibited pulmonary metastasis of osteosarcoma in the mouse model by suppressing the expression of CXC chemokine receptor 4 (CXCR4) and Rho small GTPase protein [41]. Our study provides the first evidence of the potential use of adalimumab in treating SCEL-positive TNBC patients with pulmonary metastasis and SCEL could serve as a therapeutic biomarker for identifying the suitable patients for such therapy. Furthermore, the study could be expanded to investigate the role of SCEL in other subtypes of breast cancer and in other types of cancer with a propensity for lung metastasis. Finally, the study could be extended to assess the potential for the combination of adalimumab with other targeted therapies for the treatment of TNBC.

## Conclusions

Our study provides novel insights into how metastatic TNBC cells could harness the TNF-α/TNFR1-mediated NF-κB/c-FLIP cell survival and proliferation pathway to effectively promote its lung colonization by upregulating SCEL expression to sustain TNFR1 stability. Blocking the TNF-α-mediated survival pathway by adalimumab may be an effective therapy for SCEL-positive TNBC patients with pulmonary metastatic disease. More extensive preclinical and clinical studies are needed to validate this approach and assess potential adverse effects. In the future, our study could be expanded to investigate the role of SCEL in other subtypes of breast cancer and in other types of cancer with a propensity for lung metastasis. Additionally, the potential combination of adalimumab with chemotherapy or other targeted therapies could be evaluated for improving the treatment of mTNBC.

### Supplementary Information

**Additional file 1:** **Fig. S1**. A supervised cluster analysis of LC/IV2 membrane (M) list in TCGA BRCA dataset using UCSC Xena platform. **Fig. S2.  **SCEL protein expression in non-TNBC tissue samples. **Fig. S3. **The effect of SCEL depletion on LC cells in response to the treatment of growth factors and inflammatory cytokine. **Fig. S4. **SCEL protein expression significantly associated with TNFR1 protein expression levels in TNBC specimens. **Table S1. **The iTRAQ-generated LC membrane list. **Table S2. **The iTRAQ-generated IV2_membrane list. **Table S3.** Full list of the Itraq-generated LC membrane list. **Table S4. **Full list of the iTRAQ-generated IV2 membrane list.**Table S5. **Reagents and antibodies.**Table S6. **ShRNAs and oligo primers.

**Additional file 2**: **Fig. S6. **Cell line authentication: Short tandem repeat (STR) analysis of 231-PT. **Fig. S7. **Cell line authentication: Short tandem repeat (STR) analysis of 231-LC. **Fig. S8. **Cell line authentication: Short tandem repeat (STR) analysis of 231-IV2. **Fig. S9**. Cell line deposition at Bioresource Collection and Research Center (BCRC) at Food Industry Research and Development Institute, Hsinchu, Taiwan.

**Additional file 3: Fig. S1.** Uncropped Western blots.

## Data Availability

All data are included in this published article and Additional files.

## Abbreviations

iTRAQ: The isobaric tag for relative and absolute quantification
LC–MS/MS: Liquid chromatography coupled with tandem mass spectrometry
SCEL: Sciellin
TNF-α: Tumor necrosis factor-α
IGF-1: Insulin growth factor-1
EGF: Epidermal growth factor
CHX: Cycloheximide
NF-κB: Nuclear factor kappa-light-chain-enhancer of activated B cells
test: Interlukin-6
TCGA: The Cancer Genome Atlas
c-FLIP: Cellular FLICE (FADD-like IL-1β-converting enzyme)-inhibitory protein
TNFR1: Tumor necrosis factor receptor-1
