# Supplementary material for: SCEL regulates switches between pro-survival and apoptosis of the TNF-α/TNFR1/NF-κB/c-FLIP axis to control lung colonization of triple negative breast cancer
Source: J Biomed Sci. 2023 Nov 30;30:93. doi: 10.1186/s12929-023-00986-4 (PMC10688137; doi:10.1186/s12929-023-00986-4)
Supplement: Supplementary file 1 — Additional file 1: Fig. S1. A supervised cluster analysis of LC/IV2 membrane (M) list in TCGA BRCA dataset using UCSC Xena platform. Fig. S2. SCEL protein expression in non-TNBC tissue samples. Fig. S3. The effect of SCEL depletion on LC cells in response to the treatment of growth factors and inflammatory cytokine. Fig. S4. SCEL protein expression significantly associated with TNFR1 protein expression levels in TNBC specimens. Table S1. The iTRAQ-generated LC membrane list. Table S2. The iTRAQ-generated IV2_membrane list. Table S3. Full list of the Itraq-generated LC membrane list. Table S4. Full list of the iTRAQ-generated IV2 membrane list.Table S5. Reagents and antibodies.Table S6. ShRNAs and oligo primers. [file 12929_2023_986_MOESM1_ESM.docx]

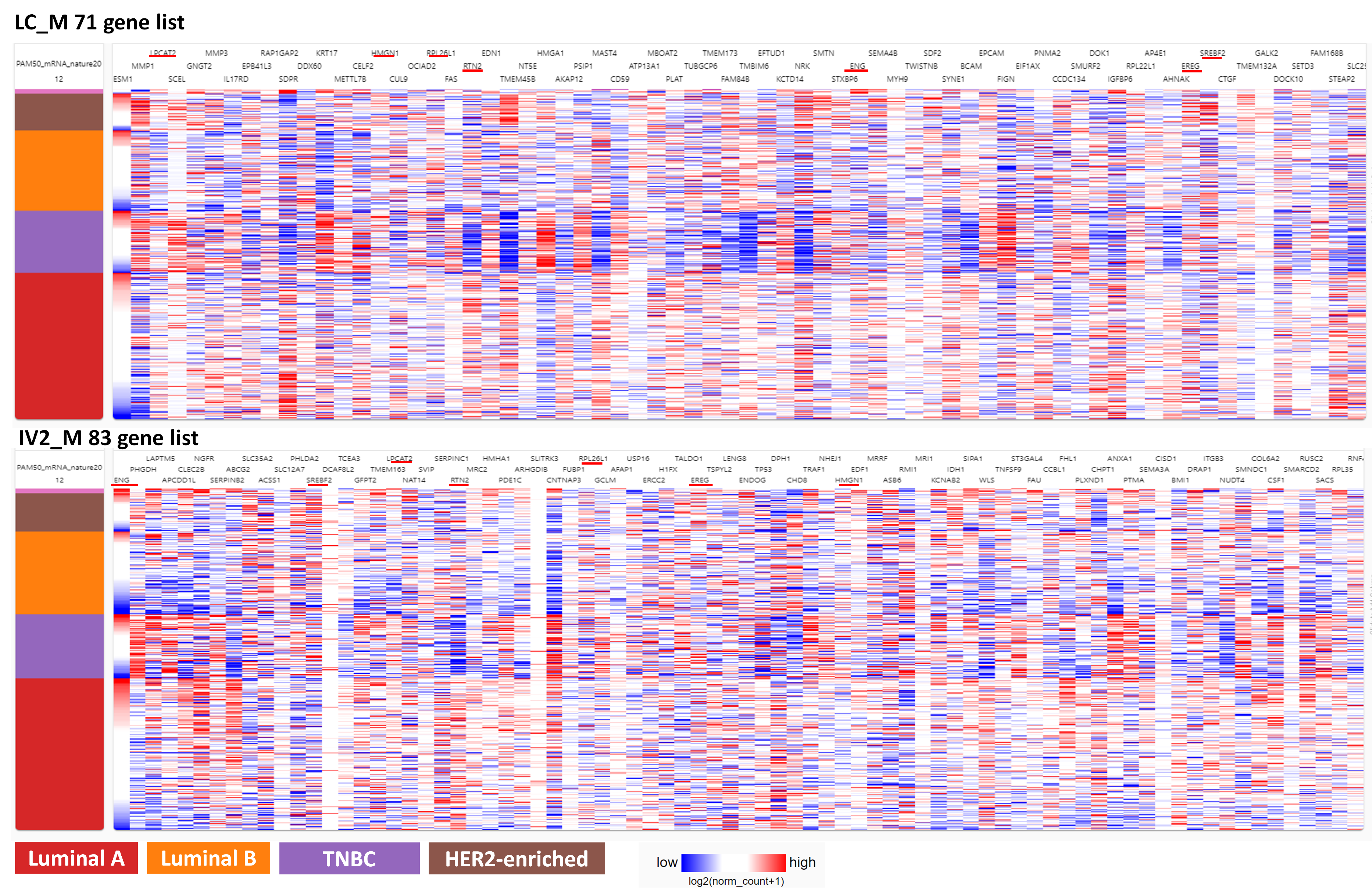


**Additional Fig. S1. A supervised cluster analysis of LC/IV2 membrane (M) list in TCGA BRCA dataset using UCSC Xena platform.** A supervised cluster analysis of the LC (71) and IV2 (83) M list. Gene symbol of 64 LC-specific genes and 76 IV2-specific genes were shown. Genes underlined were the common 7 genes present in both lists.


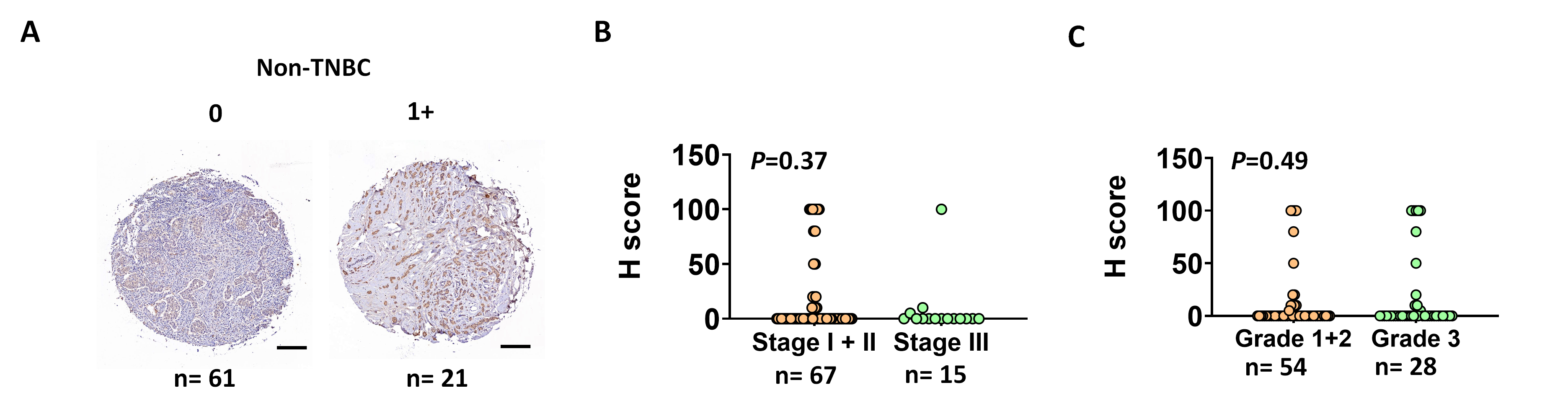


**Additional Fig. S2. SCEL protein expression in non-TNBC tissue samples.** **A.** Representative images of immunohistochemistry (IHC) staining of SCEL protein expression in non-TNBC tissue microarrays (n=82). Scale bar, 200 μm. **B**, IHC H score of SCEL in the early-stage and late-stage TNBC tumors. **C**. IHC H score of SCEL in the lower and higher grade TNBC tumors.


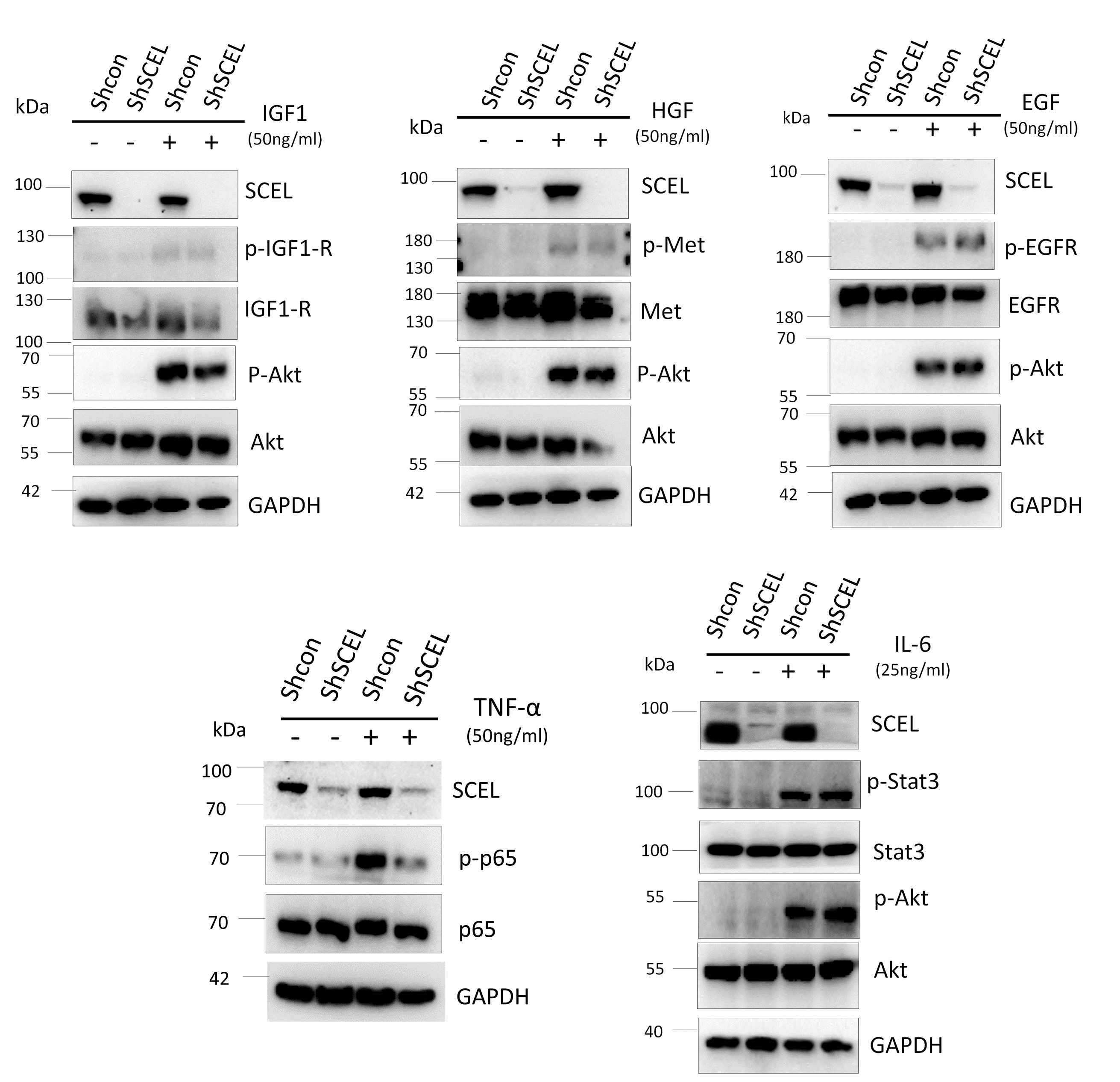


**Additional Fig. S3. The effect of SCEL depletion on LC cells in response to the treatment of growth factors and inflammatory cytokine**. The control LC cells and SCEL-depleted LC cells were treated with IGF-1, HGF, EGF, IL-6, and TNF-α at the indicated dosage, respectively. Each experiment was performed in triplicates and was repeated at least 3 times.


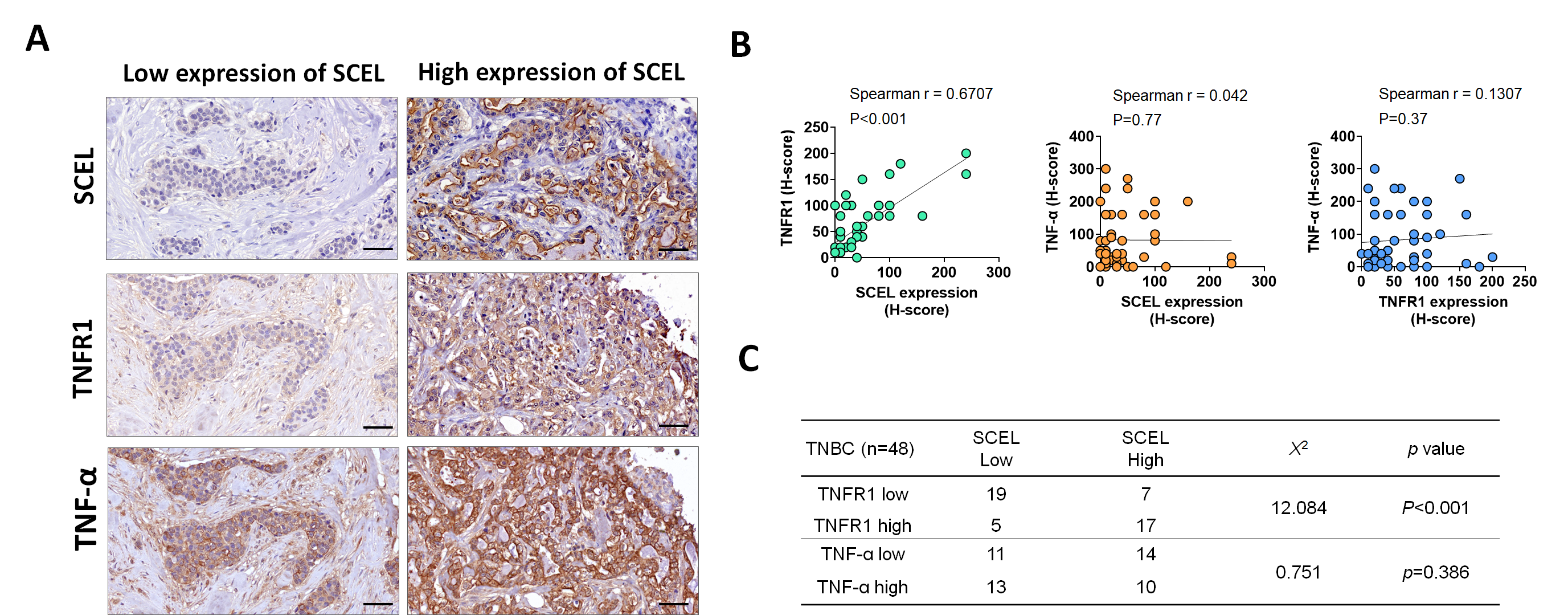


**Additional Fig. S4. SCEL protein expression significantly associated with TNFR1 protein expression levels in TNBC specimens. A**. Representative images of immunohistochemistry staining (IHC) for SCEL, TNFR1, and TNF-α in TNBC tissue microarrays. **B**. Correlation analysis between SCEL, TNFR1, and TNF-α using Pearson’s correlation coefficient (rho). **C**. Examination of clinical association between SCEL and TNFR1 expression using Chi-square analysis.

**Additional Table S1. The iTRAQ-generated LC membrane list.**

| **Accession** | **Gene symbol** | **Coverage** | **# Peptides** | **# PSMs** | **# Unique Peptides** | **MW [kDa]** | **calc. pI** | **NorLog2  LC/231** |
| --- | --- | --- | --- | --- | --- | --- | --- | --- |
| Q9NQ30 | ESM1 | 15.2173913 | 2 | 2 | 2 | 20.1 | 7.34 | 2.16 |
| P03956 | MMP1 | 52.45202559 | 21 | 64 | 21 | 54 | 6.96 | 1.87 |
| Q7L5N7 | LPCAT2* | 18.56617647 | 8 | 11 | 8 | 60.2 | 6.55 | 1.86 |
| O95171 | SCEL | 29.50581395 | 18 | 19 | 18 | 77.5 | 9.38 | 1.71 |
| O14610 | GNGT2 | 42.02898551 | 5 | 9 | 4 | 7.7 | 6.68 | 1.67 |
| P08254 | MMP3 | 18.44863732 | 9 | 11 | 9 | 53.9 | 6.16 | 1.62 |
| Q8NFM7 | I17RD | 2.029769959 | 2 | 3 | 2 | 82.4 | 7.18 | 1.53 |
| Q9Y2J2 | E41L3 | 35.6025759 | 30 | 53 | 22 | 120.6 | 5.19 | 1.43 |
| Q684P5 | RPGP2 | 3.97260274 | 3 | 4 | 3 | 80 | 6.67 | 1.32 |
| O95810 | SDPR | 54.11764706 | 24 | 71 | 24 | 47.1 | 5.21 | 1.31 |
| Q8IY21 | DDX60 | 1.635514019 | 3 | 3 | 2 | 197.7 | 7.59 | 1.25 |
| Q04695 | K1C17 | 19.21296296 | 10 | 14 | 2 | 48.1 | 5.02 | 1.23 |
| Q6UX53 | MET7B | 14.3442623 | 3 | 4 | 3 | 27.8 | 8.38 | 1.21 |
| O95319 | CELF2 | 20.27559055 | 8 | 14 | 7 | 54.3 | 8.76 | 1.19 |
| Q8IWT3 | CUL9 | 1.708382996 | 4 | 4 | 2 | 281 | 5.45 | 1.19 |
| P05114 | HMGN1* | 21 | 2 | 4 | 2 | 10.7 | 9.6 | 1.19 |
| Q56VL3 | OCAD2 | 53.8961039 | 9 | 32 | 9 | 16.9 | 9.03 | 1.18 |
| Q9UNX3 | RPL26L1* | 45.51724138 | 10 | 24 | 3 | 17.2 | 10.55 | 1.17 |
| P25445 | TNR6 | 25.37313433 | 7 | 11 | 7 | 37.7 | 7.94 | 1.14 |
| O75298 | RTN2* | 9.174311927 | 5 | 5 | 5 | 59.2 | 5.31 | 1.13 |
| P05305 | EDN1 | 7.075471698 | 2 | 2 | 2 | 24.4 | 9.41 | 1.07 |
| Q96B21 | TM45B | 6.181818182 | 2 | 2 | 2 | 31.8 | 6.95 | 1.07 |
| P21589 | 5NTD | 51.2195122 | 23 | 108 | 23 | 63.3 | 7.03 | 1.04 |
| P17096 | HMGA1 | 48.59813084 | 7 | 44 | 7 | 11.7 | 10.32 | 1.03 |
| Q02952 | AKA12 | 9.315375982 | 9 | 12 | 9 | 191.4 | 4.41 | 1.01 |
| O75475 | PSIP1 | 7.169811321 | 3 | 7 | 2 | 60.1 | 9.13 | 1.01 |
| O15021 | MAST4 | 1.599390708 | 3 | 3 | 2 | 284.2 | 8.62 | 1.01 |
| P13987 | CD59 | 25 | 4 | 22 | 4 | 14.2 | 6.48 | 0.98 |
| Q9HD20 | AT131 | 30.14950166 | 31 | 51 | 31 | 132.9 | 8.13 | 0.98 |
| Q6ZWT7 | MBOA2 | 27.11538462 | 12 | 21 | 12 | 59.5 | 8.03 | 0.97 |
| P00750 | TPA | 46.97508897 | 20 | 52 | 20 | 62.9 | 7.8 | 0.95 |
| Q9NXH8 | TOR4A | 26.47754137 | 10 | 13 | 10 | 46.9 | 9.94 | 0.94 |
| Q96RT7 | GCP6 | 4.123144585 | 5 | 7 | 5 | 200.4 | 6.32 | 0.94 |
| Q86WV6 | STING | 27.70448549 | 7 | 9 | 7 | 42.2 | 7.05 | 0.93 |
| Q96KN1 | FA84B | 49.03225806 | 11 | 16 | 11 | 34.5 | 5.54 | 0.89 |
| P55061 | BI1 | 8.860759494 | 2 | 4 | 2 | 26.5 | 8.54 | 0.89 |
| Q7Z2Z2 | ETUD1 | 16.51785714 | 13 | 22 | 13 | 125.4 | 5.91 | 0.89 |
| Q9BQ13 | KCD14 | 11.76470588 | 3 | 5 | 3 | 29.6 | 8.59 | 0.87 |
| Q7Z2Y5 | NRK | 9.165613148 | 11 | 11 | 11 | 178.4 | 6.33 | 0.87 |
| P53814 | SMTN | 31.84296619 | 23 | 31 | 23 | 99 | 9.07 | 0.86 |
| Q8NFX7 | STXB6 | 23.33333333 | 5 | 6 | 5 | 23.5 | 9.04 | 0.85 |
| P17813 | ENG* | 6.838905775 | 3 | 3 | 3 | 70.5 | 6.61 | 0.85 |
| Q9NPR2 | SEM4B | 19.95192308 | 13 | 14 | 13 | 92.1 | 6.95 | 0.84 |
| P35579 | MYH9 | 62.90816327 | 132 | 524 | 113 | 226.4 | 5.6 | 0.84 |
| Q3B726 | RPA43 | 7.396449704 | 2 | 2 | 2 | 37.4 | 6.98 | 0.84 |
| Q99470 | SDF2 | 36.492891 | 5 | 13 | 5 | 23 | 7.33 | 0.83 |
| Q8NF91 | SYNE1 | 8.548368762 | 59 | 70 | 59 | 1010.5 | 5.53 | 0.82 |
| P50895 | BCAM | 25.31847134 | 10 | 11 | 10 | 67.4 | 5.81 | 0.80 |
| P16422 | EPCAM | 9.872611465 | 3 | 4 | 3 | 34.9 | 7.46 | 0.80 |
| Q5HY92 | FIGN | 4.743083004 | 3 | 3 | 3 | 82.1 | 6.71 | 0.79 |
| P47813 | IF1AX | 47.22222222 | 5 | 8 | 5 | 16.5 | 5.24 | 0.79 |
| Q9UL42 | PNMA2 | 9.615384615 | 4 | 5 | 4 | 41.5 | 4.86 | 0.78 |
| Q9H6E4 | CC134 | 24.89082969 | 5 | 8 | 5 | 26.5 | 8.85 | 0.78 |
| P24592 | IBP6 | 14.58333333 | 2 | 4 | 2 | 25.3 | 7.81 | 0.77 |
| Q9HAU4 | SMUF2 | 20.58823529 | 16 | 21 | 16 | 86.1 | 7.96 | 0.77 |
| Q99704 | DOK1 | 10.81081081 | 3 | 3 | 3 | 52.4 | 6.47 | 0.77 |
| Q6P5R6 | RL22L | 15.57377049 | 2 | 3 | 2 | 14.6 | 9.38 | 0.76 |
| Q9UPM8 | AP4E1 | 3.254177661 | 3 | 3 | 3 | 127.2 | 5.99 | 0.76 |
| Q09666 | AHNK | 71.51103565 | 250 | 747 | 250 | 628.7 | 6.15 | 0.76 |
| O14944 | EREG* | 10.0591716 | 2 | 3 | 2 | 19 | 7.53 | 0.76 |
| Q12772 | SREBF2* | 3.856266433 | 4 | 4 | 4 | 123.6 | 8.4 | 0.76 |
| Q24JP5 | T132A | 2.737047898 | 2 | 2 | 2 | 110 | 5.62 | 0.75 |
| P29279 | CTGF | 43.26647564 | 14 | 25 | 14 | 38.1 | 8 | 0.75 |
| Q01415 | GALK2 | 5.021834061 | 2 | 2 | 2 | 50.3 | 6.61 | 0.75 |
| Q96BY6 | DOC10 | 17.15462031 | 33 | 41 | 32 | 249.4 | 7.14 | 0.74 |
| A1KXE4 | F168B | 23.58974359 | 2 | 3 | 2 | 20.3 | 9.23 | 0.73 |
| Q86TU7 | SETD3 | 16.16161616 | 9 | 9 | 9 | 67.2 | 5.96 | 0.73 |
| Q5VT66 | MARC1 | 11.8694362 | 4 | 4 | 3 | 37.5 | 8.88 | 0.72 |
| Q8NFT2 | STEA2 | 5.306122449 | 3 | 3 | 3 | 56 | 9.2 | 0.71 |
| Q8IZF2 | AGRF5 | 19.83655275 | 23 | 51 | 23 | 149.4 | 6.65 | 0.70 |
| Q9BQT8 | ODC | 13.71237458 | 4 | 4 | 4 | 33.3 | 9.51 | 0.70 |

*Common genes in IV2 and LC list.

**Additional Table S2. The iTRAQ-generated IV2_membrane list**

| **Accession** | **Gene symbol** | **Coverage** | **# Peptides** | **# PSMs** | **# Unique Peptides** | **MW [kDa]** | **calc. pI** | **NorLog2  IV2/231** |
| --- | --- | --- | --- | --- | --- | --- | --- | --- |
| P17813 | ENG* | 6.838905775 | 3 | 3 | 3 | 70.5 | 6.61 | 1.61 |
| O43175 | SERA | 15.75984991 | 7 | 8 | 7 | 56.6 | 6.71 | 1.54 |
| Q13571 | LAPM5 | 14.1221374 | 3 | 7 | 3 | 29.9 | 8.68 | 1.53 |
| Q8NCL9 | APCDL | 4.590818363 | 2 | 2 | 2 | 55.6 | 8.85 | 1.45 |
| Q92478 | CLC2B | 14.76510067 | 2 | 2 | 2 | 17.3 | 8.75 | 1.34 |
| P08138 | TNR16 | 14.51990632 | 4 | 7 | 4 | 45.2 | 4.7 | 1.33 |
| P05120 | PAI2 | 7.228915663 | 2 | 3 | 2 | 46.6 | 5.63 | 1.3 |
| Q9UNQ0 | ABCG2 | 2.595419847 | 2 | 2 | 2 | 72.3 | 8.69 | 1.22 |
| P78381 | S35A2 | 18.68686869 | 3 | 9 | 3 | 41.3 | 9.96 | 1.12 |
| Q9NUB1 | ACS2L | 21.48040639 | 12 | 18 | 12 | 74.8 | 7.11 | 1.11 |
| Q9Y666 | S12A7 | 11.81902124 | 10 | 11 | 9 | 119 | 6.71 | 1.1 |
| Q53GA4 | PHLA2 | 20.39473684 | 4 | 8 | 4 | 17.1 | 9.17 | 1.09 |
| Q12772 | SREBF2* | 3.856266433 | 4 | 4 | 4 | 123.6 | 8.4 | 1.07 |
| P0C7V8 | DC8L2 | 3.803486529 | 2 | 2 | 2 | 71.1 | 4.73 | 1.06 |
| O75764 | TCEA3 | 8.908045977 | 2 | 2 | 2 | 38.9 | 9.19 | 1.03 |
| O94808 | GFPT2 | 25.95307918 | 16 | 19 | 13 | 76.9 | 7.37 | 1.03 |
| Q8TC26 | TM163 | 9.342560554 | 2 | 2 | 2 | 31.4 | 7.53 | 1.02 |
| Q7L5N7 | LPCAT2* | 18.56617647 | 8 | 11 | 8 | 60.2 | 6.55 | 1.01 |
| Q8WUY8 | NAT14 | 14.5631068 | 2 | 3 | 2 | 21.6 | 10.74 | 1.01 |
| Q8NHG7 | SVIP | 41.55844156 | 3 | 4 | 3 | 8.4 | 8.91 | 0.93 |
| P01008 | ANT3 | 4.310344828 | 2 | 2 | 2 | 52.6 | 6.71 | 0.93 |
| O75298 | RTN2* | 9.174311927 | 5 | 5 | 5 | 59.2 | 5.31 | 0.89 |
| Q9NS25 | SPNXB | 57.2815534 | 5 | 13 | 3 | 11.8 | 6.15 | 0.89 |
| Q9UBG0 | MRC2 | 11.89993239 | 14 | 22 | 14 | 166.6 | 5.83 | 0.88 |
| Q92619 | HMHA1 | 2.200704225 | 2 | 2 | 2 | 124.5 | 6.1 | 0.88 |
| Q14123 | PDE1C | 4.936530324 | 3 | 4 | 3 | 80.7 | 8.82 | 0.87 |
| P52566 | GDIR2 | 65.67164179 | 10 | 21 | 10 | 23 | 5.21 | 0.87 |
| Q5H943 | KKLC1 | 30.97345133 | 4 | 7 | 4 | 12.8 | 10.2 | 0.86 |
| O94933 | SLIK3 | 5.527123849 | 3 | 3 | 3 | 108.9 | 7.37 | 0.86 |
| Q9BZ76 | CNTP3 | 4.968944099 | 5 | 5 | 5 | 140.6 | 7.78 | 0.85 |
| Q96AE4 | FUBP1 | 54.03726708 | 29 | 54 | 23 | 67.5 | 7.61 | 0.85 |
| Q9UNX3 | RPL26L1* | 45.51724138 | 10 | 24 | 3 | 17.2 | 10.55 | 0.84 |
| P48507 | GSH0 | 29.9270073 | 6 | 7 | 6 | 30.7 | 6.02 | 0.84 |
| Q8N556 | AFAP1 | 13.83561644 | 8 | 11 | 8 | 80.7 | 8.68 | 0.82 |
| Q9BYV8 | CEP41 | 12.60053619 | 3 | 3 | 3 | 41.3 | 8.32 | 0.82 |
| Q9Y5T5 | UBP16 | 8.262454435 | 4 | 4 | 4 | 93.5 | 6.93 | 0.82 |
| P18074 | ERCC2 | 10.26315789 | 6 | 7 | 6 | 86.9 | 7.15 | 0.81 |
| Q92522 | H1X | 14.08450704 | 3 | 3 | 3 | 22.5 | 10.76 | 0.81 |
| P37837 | TALDO | 46.58753709 | 17 | 41 | 17 | 37.5 | 6.81 | 0.81 |
| O14944 | EREG* | 10.0591716 | 2 | 3 | 2 | 19 | 7.53 | 0.8 |
| Q9H2G4 | TSYL2 | 2.597402597 | 2 | 2 | 2 | 79.4 | 4.58 | 0.8 |
| Q96PV6 | LENG8 | 9.499358151 | 6 | 8 | 6 | 86.1 | 9.35 | 0.8 |
| Q14249 | NUCG | 23.90572391 | 6 | 9 | 6 | 32.6 | 9.5 | 0.8 |
| P04637 | P53 | 35.11450382 | 11 | 23 | 11 | 43.6 | 6.79 | 0.8 |
| Q9BZG8 | DPH1 | 9.029345372 | 3 | 3 | 3 | 48.8 | 8.18 | 0.8 |
| Q9HCK8 | CHD8 | 1.975978303 | 5 | 6 | 4 | 290.3 | 6.47 | 0.79 |
| Q13077 | TRAF1 | 9.134615385 | 3 | 3 | 3 | 46.1 | 6.11 | 0.79 |
| Q9H9Q4 | NHEJ1 | 8.361204013 | 2 | 2 | 2 | 33.3 | 5.97 | 0.79 |
| P05114 | HMGN1* | 21 | 2 | 4 | 2 | 10.7 | 9.6 | 0.78 |
| O60869 | EDF1 | 42.56756757 | 7 | 12 | 7 | 16.4 | 9.95 | 0.78 |
| Q96E11 | RRFM | 29.00763359 | 7 | 12 | 7 | 29.3 | 9.79 | 0.78 |
| Q9NWX5 | ASB6 | 6.888361045 | 2 | 2 | 2 | 47.1 | 6.01 | 0.78 |
| Q9H9A7 | RMI1 | 5.6 | 2 | 3 | 2 | 70.1 | 4.96 | 0.78 |
| Q9BV20 | MTNA | 10.8401084 | 3 | 3 | 3 | 39.1 | 6.3 | 0.78 |
| Q13303 | KCAB2 | 19.89100817 | 5 | 6 | 5 | 41 | 9 | 0.78 |
| O75874 | IDHC | 40.82125604 | 14 | 26 | 13 | 46.6 | 7.01 | 0.77 |
| Q96FS4 | SIPA1 | 4.798464491 | 4 | 5 | 4 | 112.1 | 6.6 | 0.76 |
| Q5T9L3 | WLS | 15.52680222 | 8 | 10 | 8 | 62.2 | 7.36 | 0.76 |
| P41273 | TNFL9 | 9.05511811 | 2 | 2 | 2 | 26.6 | 7.02 | 0.76 |
| Q11206 | SIA4C | 9.60960961 | 3 | 4 | 3 | 38 | 9.41 | 0.75 |
| P62861 | RS30 | 18.6440678 | 2 | 6 | 2 | 6.6 | 12.15 | 0.75 |
| Q16773 | KAT1 | 12.55924171 | 5 | 6 | 4 | 47.8 | 6.47 | 0.75 |
| Q13642 | FHL1 | 11.76470588 | 4 | 7 | 4 | 36.2 | 8.97 | 0.75 |
| Q9Y4D7 | PLXD1 | 1.662337662 | 3 | 4 | 2 | 211.9 | 7.15 | 0.75 |
| Q8WUD6 | CHPT1 | 8.620689655 | 3 | 3 | 2 | 45.1 | 6.92 | 0.74 |
| P04083 | ANXA1 | 67.05202312 | 21 | 70 | 21 | 38.7 | 7.02 | 0.74 |
| P06454 | PTMA | 36.03603604 | 7 | 81 | 7 | 12.2 | 3.78 | 0.73 |
| Q14563 | SEM3A | 4.928664073 | 4 | 4 | 4 | 88.8 | 7.42 | 0.72 |
| Q9NZ45 | CISD1 | 52.77777778 | 5 | 12 | 5 | 12.2 | 9.09 | 0.72 |
| P35226 | BMI1 | 5.828220859 | 2 | 2 | 2 | 36.9 | 8.63 | 0.71 |
| Q14687 | GSE1 | 2.875924404 | 3 | 3 | 3 | 136.1 | 7.74 | 0.71 |
| Q14919 | NC2A | 24.87804878 | 4 | 8 | 4 | 22.3 | 5.17 | 0.71 |
| P05106 | ITB3 | 5.837563452 | 4 | 5 | 4 | 87 | 5.24 | 0.71 |
| Q9NZJ9 | NUDT4 | 21.11111111 | 4 | 4 | 3 | 20.3 | 6.35 | 0.71 |
| O75940 | SPF30 | 21.8487395 | 5 | 8 | 5 | 26.7 | 7.24 | 0.71 |
| P12110 | CO6A2 | 2.747791953 | 3 | 3 | 3 | 108.5 | 6.21 | 0.71 |
| P09603 | CSF1 | 32.31046931 | 14 | 24 | 14 | 60.1 | 5.29 | 0.71 |
| Q92925 | SMRD2 | 5.838041431 | 2 | 2 | 2 | 58.9 | 9.64 | 0.7 |
| Q8N2Y8 | RUSC2 | 4.023746702 | 3 | 3 | 3 | 161.1 | 6.62 | 0.7 |
| Q9NZJ4 | SACS | 1.528718061 | 6 | 7 | 6 | 520.8 | 7.05 | 0.7 |
| P42766 | RL35 | 21.95121951 | 4 | 15 | 4 | 14.5 | 11.05 | 0.7 |
| P78317 | RNF4 | 25.78947368 | 3 | 3 | 3 | 21.3 | 7.03 | 0.7 |
| Q8N0V3 | RBFA | 9.329446064 | 3 | 4 | 3 | 38.3 | 7.85 | 0.7 |

* Common genes in IV2 and LC list.

**Additional Table S3. Full list of the Itraq-generated LC membrane list**

| **Gene symbol** | **# Unique Peptides** | **MW [kDa]** | **calc. Pi** | **Abundance Ratio (log2): LC/231** | **NorLog2 LC/231** |
| --- | --- | --- | --- | --- | --- |
| ESM1 | 2 | 20.1 | 7.34 | 1.95 | 2.16 |
| MMP1 | 21 | 54 | 6.96 | 1.66 | 1.87 |
| LPCAT2* | 8 | 60.2 | 6.55 | 1.65 | 1.86 |
| SCEL | 18 | 77.5 | 9.38 | 1.5 | 1.71 |
| GNGT2 | 4 | 7.7 | 6.68 | 1.46 | 1.67 |
| MMP3 | 9 | 53.9 | 6.16 | 1.41 | 1.62 |
| I17RD | 2 | 82.4 | 7.18 | 1.32 | 1.53 |
| E41L3 | 22 | 120.6 | 5.19 | 1.22 | 1.43 |
| RPGP2 | 3 | 80 | 6.67 | 1.11 | 1.32 |
| SDPR | 24 | 47.1 | 5.21 | 1.1 | 1.31 |
| DDX60 | 2 | 197.7 | 7.59 | 1.04 | 1.25 |
| K1C17 | 2 | 48.1 | 5.02 | 1.02 | 1.23 |
| MET7B | 3 | 27.8 | 8.38 | 1 | 1.21 |
| CELF2 | 7 | 54.3 | 8.76 | 0.98 | 1.19 |
| CUL9 | 2 | 281 | 5.45 | 0.98 | 1.19 |
| HMGN1* | 2 | 10.7 | 9.6 | 0.98 | 1.19 |
| OCAD2 | 9 | 16.9 | 9.03 | 0.97 | 1.18 |
| RPL26L1* | 3 | 17.2 | 10.55 | 0.96 | 1.17 |
| TNR6 | 7 | 37.7 | 7.94 | 0.93 | 1.14 |
| RTN2* | 5 | 59.2 | 5.31 | 0.92 | 1.13 |
| EDN1 | 2 | 24.4 | 9.41 | 0.86 | 1.07 |
| TM45B | 2 | 31.8 | 6.95 | 0.86 | 1.07 |
| 5NTD | 23 | 63.3 | 7.03 | 0.83 | 1.04 |
| HMGA1 | 7 | 11.7 | 10.32 | 0.82 | 1.03 |
| AKA12 | 9 | 191.4 | 4.41 | 0.8 | 1.01 |
| PSIP1 | 2 | 60.1 | 9.13 | 0.8 | 1.01 |
| MAST4 | 2 | 284.2 | 8.62 | 0.8 | 1.01 |
| CD59 | 4 | 14.2 | 6.48 | 0.77 | 0.98 |
| AT131 | 31 | 132.9 | 8.13 | 0.77 | 0.98 |
| MBOA2 | 12 | 59.5 | 8.03 | 0.76 | 0.97 |
| TPA | 20 | 62.9 | 7.8 | 0.74 | 0.95 |
| TOR4A | 10 | 46.9 | 9.94 | 0.73 | 0.94 |
| GCP6 | 5 | 200.4 | 6.32 | 0.73 | 0.94 |
| STING | 7 | 42.2 | 7.05 | 0.72 | 0.93 |
| FA84B | 11 | 34.5 | 5.54 | 0.68 | 0.89 |
| BI1 | 2 | 26.5 | 8.54 | 0.68 | 0.89 |
| ETUD1 | 13 | 125.4 | 5.91 | 0.68 | 0.89 |
| KCD14 | 3 | 29.6 | 8.59 | 0.66 | 0.87 |
| NRK | 11 | 178.4 | 6.33 | 0.66 | 0.87 |
| SMTN | 23 | 99 | 9.07 | 0.65 | 0.86 |
| STXB6 | 5 | 23.5 | 9.04 | 0.64 | 0.85 |
| ENG* | 3 | 70.5 | 6.61 | 0.64 | 0.85 |
| SEM4B | 13 | 92.1 | 6.95 | 0.63 | 0.84 |
| MYH9 | 113 | 226.4 | 5.6 | 0.63 | 0.84 |
| RPA43 | 2 | 37.4 | 6.98 | 0.63 | 0.84 |
| SDF2 | 5 | 23 | 7.33 | 0.62 | 0.83 |
| SYNE1 | 59 | 1010.5 | 5.53 | 0.61 | 0.82 |
| BCAM | 10 | 67.4 | 5.81 | 0.59 | 0.8 |
| EPCAM | 3 | 34.9 | 7.46 | 0.59 | 0.8 |
| FIGN | 3 | 82.1 | 6.71 | 0.58 | 0.79 |
| IF1AX | 5 | 16.5 | 5.24 | 0.58 | 0.79 |
| PNMA2 | 4 | 41.5 | 4.86 | 0.57 | 0.78 |
| CC134 | 5 | 26.5 | 8.85 | 0.57 | 0.78 |
| IBP6 | 2 | 25.3 | 7.81 | 0.56 | 0.77 |
| SMUF2 | 16 | 86.1 | 7.96 | 0.56 | 0.77 |
| DOK1 | 3 | 52.4 | 6.47 | 0.56 | 0.77 |
| RL22L | 2 | 14.6 | 9.38 | 0.55 | 0.76 |
| AP4E1 | 3 | 127.2 | 5.99 | 0.55 | 0.76 |
| AHNK | 250 | 628.7 | 6.15 | 0.55 | 0.76 |
| EREG* | 2 | 19 | 7.53 | 0.55 | 0.76 |
| SREBF2* | 4 | 123.6 | 8.4 | 0.55 | 0.76 |
| T132A | 2 | 110 | 5.62 | 0.54 | 0.75 |
| CTGF | 14 | 38.1 | 8 | 0.54 | 0.75 |
| GALK2 | 2 | 50.3 | 6.61 | 0.54 | 0.75 |
| DOC10 | 32 | 249.4 | 7.14 | 0.53 | 0.74 |
| F168B | 2 | 20.3 | 9.23 | 0.52 | 0.73 |
| SETD3 | 9 | 67.2 | 5.96 | 0.52 | 0.73 |
| MARC1 | 3 | 37.5 | 8.88 | 0.51 | 0.72 |
| STEA2 | 3 | 56 | 9.2 | 0.5 | 0.71 |
| AGRF5 | 23 | 149.4 | 6.65 | 0.49 | 0.7 |
| ODC | 4 | 33.3 | 9.51 | 0.49 | 0.7 |
| PLAK | 15 | 81.7 | 6.14 | -0.91 | -0.7 |
| K2C8 | 30 | 53.7 | 5.59 | -0.91 | -0.7 |
| CGL | 8 | 44.5 | 6.7 | -0.91 | -0.7 |
| ISG15 | 8 | 17.9 | 7.44 | -0.91 | -0.7 |
| DNPH1 | 2 | 19.1 | 5.05 | -0.92 | -0.71 |
| WBP2 | 6 | 28.1 | 5.91 | -0.92 | -0.71 |
| MBNL2 | 3 | 40.5 | 8.38 | -0.92 | -0.71 |
| ATD3A | 11 | 71.3 | 8.98 | -0.92 | -0.71 |
| PYRD2 | 8 | 63 | 6.95 | -0.92 | -0.71 |
| RFFL | 4 | 40.5 | 5.48 | -0.93 | -0.72 |
| ZN428 | 4 | 20.5 | 4.17 | -0.93 | -0.72 |
| TAP1 | 16 | 87.2 | 8.02 | -0.93 | -0.72 |
| CYTC | 5 | 15.8 | 8.75 | -0.93 | -0.72 |
| 4F2 | 27 | 68 | 5.01 | -0.93 | -0.72 |
| HCDH | 14 | 34.3 | 8.85 | -0.94 | -0.73 |
| NDUA5 | 6 | 13.5 | 5.99 | -0.94 | -0.73 |
| IRGQ | 10 | 62.7 | 4.88 | -0.94 | -0.73 |
| PPME1 | 12 | 42.3 | 5.97 | -0.94 | -0.73 |
| ARH | 2 | 33.9 | 6.7 | -0.94 | -0.73 |
| ATD3B | 8 | 72.5 | 9.2 | -0.94 | -0.73 |
| CPPED | 3 | 35.5 | 6.2 | -0.95 | -0.74 |
| RND3 | 10 | 27.4 | 8.48 | -0.95 | -0.74 |
| QCR6 | 8 | 10.7 | 4.44 | -0.95 | -0.74 |
| PLPP1 | 3 | 32.1 | 7.97 | -0.95 | -0.74 |
| PLS1 | 4 | 35 | 4.94 | -0.96 | -0.75 |
| EPHB2 | 20 | 117.4 | 6.55 | -0.96 | -0.75 |
| PAR1 | 3 | 47.4 | 8.27 | -0.96 | -0.75 |
| DPH5 | 2 | 31.6 | 5.31 | -0.96 | -0.75 |
| CPT2 | 27 | 73.7 | 8.18 | -0.96 | -0.75 |
| PLS3 | 3 | 31.6 | 6.65 | -0.97 | -0.76 |
| T161B | 4 | 55.4 | 8.37 | -0.97 | -0.76 |
| VIME | 42 | 53.6 | 5.12 | -0.97 | -0.76 |
| S17A5 | 7 | 54.6 | 8.27 | -0.97 | -0.76 |
| RAB3A | 2 | 25 | 5.03 | -0.97 | -0.76 |
| TPSN | 7 | 47.6 | 7.15 | -0.97 | -0.76 |
| XCT | 4 | 55.4 | 9.19 | -0.97 | -0.76 |
| TNR16 | 4 | 45.2 | 4.7 | -0.97 | -0.76 |
| ECH1 | 16 | 35.8 | 8 | -0.98 | -0.77 |
| S39AB | 4 | 35.4 | 5.6 | -0.98 | -0.77 |
| HMGB3 | 6 | 23 | 8.37 | -0.98 | -0.77 |
| SODC | 2 | 15.9 | 6.13 | -0.98 | -0.77 |
| PLS4 | 2 | 37 | 5.82 | -0.98 | -0.77 |
| CPTP | 2 | 24.4 | 7.21 | -0.99 | -0.78 |
| HG2A | 9 | 33.5 | 8.44 | -1 | -0.79 |
| STEA3 | 10 | 54.6 | 8.6 | -1.01 | -0.8 |
| MCFD2 | 5 | 16.4 | 4.63 | -1.01 | -0.8 |
| FRIL | 3 | 20 | 5.78 | -1.01 | -0.8 |
| PTTG | 4 | 20.3 | 8.79 | -1.02 | -0.81 |
| NFIP1 | 5 | 24.9 | 4.68 | -1.02 | -0.81 |
| SC11C | 2 | 21.5 | 9.2 | -1.02 | -0.81 |
| LMBD1 | 2 | 61.3 | 7.77 | -1.03 | -0.82 |
| MOCOS | 2 | 98.1 | 6.7 | -1.03 | -0.82 |
| L1CAM | 19 | 139.9 | 6.24 | -1.03 | -0.82 |
| PODXL | 11 | 58.6 | 5.49 | -1.04 | -0.83 |
| PEN2 | 2 | 12 | 9.19 | -1.04 | -0.83 |
| LICH | 4 | 45.4 | 6.92 | -1.04 | -0.83 |
| EFC4B | 7 | 45.6 | 5.01 | -1.04 | -0.83 |
| TLR2 | 4 | 89.8 | 6.61 | -1.04 | -0.83 |
| THIM | 20 | 41.9 | 8.09 | -1.05 | -0.84 |
| AGR2 | 4 | 20 | 9 | -1.05 | -0.84 |
| GTR1 | 8 | 54 | 8.72 | -1.06 | -0.85 |
| HRSL3 | 5 | 17.9 | 7.99 | -1.06 | -0.85 |
| CATD | 16 | 44.5 | 6.54 | -1.06 | -0.85 |
| AT1B1 | 13 | 35 | 8.53 | -1.06 | -0.85 |
| SOAT1 | 13 | 64.7 | 8.94 | -1.07 | -0.86 |
| TM192 | 8 | 30.9 | 7.99 | -1.07 | -0.86 |
| EDIL3 | 18 | 53.7 | 7.28 | -1.07 | -0.86 |
| RALA | 7 | 23.6 | 7.11 | -1.1 | -0.89 |
| CF132 | 2 | 124 | 9.45 | -1.1 | -0.89 |
| BID | 4 | 22 | 5.44 | -1.1 | -0.89 |
| SDCB1 | 8 | 32.4 | 7.53 | -1.11 | -0.9 |
| CPNS1 | 9 | 28.3 | 5.2 | -1.11 | -0.9 |
| CFAH | 2 | 139 | 6.61 | -1.11 | -0.9 |
| CHRC1 | 2 | 14.7 | 5.1 | -1.12 | -0.91 |
| CR032 | 4 | 8.7 | 9.13 | -1.13 | -0.92 |
| PSG9 | 3 | 48.2 | 8.07 | -1.14 | -0.93 |
| PKHA7 | 5 | 127.1 | 9.35 | -1.14 | -0.93 |
| NCKX6 | 2 | 64.2 | 8.02 | -1.14 | -0.93 |
| ELOV7 | 3 | 33.3 | 9.26 | -1.14 | -0.93 |
| RM10 | 7 | 29.3 | 9.58 | -1.15 | -0.94 |
| PRDBP | 11 | 27.7 | 6.43 | -1.15 | -0.94 |
| CSF1 | 14 | 60.1 | 5.29 | -1.15 | -0.94 |
| FOLR1 | 4 | 29.8 | 7.97 | -1.16 | -0.95 |
| DPY30 | 4 | 11.2 | 4.88 | -1.17 | -0.96 |
| ANT3 | 2 | 52.6 | 6.71 | -1.17 | -0.96 |
| ENDD1 | 13 | 55 | 5.71 | -1.18 | -0.97 |
| SCMC1 | 23 | 53.3 | 6.33 | -1.18 | -0.97 |
| LAMP2 | 9 | 44.9 | 5.63 | -1.18 | -0.97 |
| CALB2 | 3 | 31.5 | 5.15 | -1.18 | -0.97 |
| CYB5 | 6 | 15.3 | 4.96 | -1.18 | -0.97 |
| SLAF7 | 9 | 37.4 | 6.43 | -1.19 | -0.98 |
| SCRB1 | 7 | 60.8 | 8.24 | -1.21 | -1 |
| LHPL2 | 2 | 24.5 | 6.49 | -1.22 | -1.01 |
| PPB1 | 18 | 57.9 | 6.29 | -1.24 | -1.03 |
| SYSM | 21 | 58.2 | 8.13 | -1.24 | -1.03 |
| TACD2 | 10 | 35.7 | 8.87 | -1.25 | -1.04 |
| GNAS1 | 15 | 111 | 5.03 | -1.26 | -1.05 |
| SPX2 | 4 | 54.4 | 6.81 | -1.27 | -1.06 |
| PLD3 | 9 | 54.7 | 6.47 | -1.27 | -1.06 |
| AN32E | 8 | 30.7 | 3.85 | -1.27 | -1.06 |
| K2C7 | 18 | 51.4 | 5.48 | -1.28 | -1.07 |
| TXN4A | 2 | 16.8 | 5.85 | -1.28 | -1.07 |
| HKDC1 | 7 | 102.5 | 7.12 | -1.29 | -1.08 |
| CD82 | 5 | 29.6 | 5.24 | -1.29 | -1.08 |
| DAF | 15 | 41.4 | 7.59 | -1.3 | -1.09 |
| TM177 | 3 | 33.7 | 9.61 | -1.3 | -1.09 |
| KCRB | 11 | 42.6 | 5.59 | -1.31 | -1.1 |
| GRPE2 | 6 | 25.4 | 7.72 | -1.31 | -1.1 |
| CYAC3 | 2 | 27.2 | 9.6 | -1.31 | -1.1 |
| S15A3 | 3 | 63.5 | 9.06 | -1.32 | -1.11 |
| OAS2 | 2 | 82.4 | 8.25 | -1.33 | -1.12 |
| K1C18 | 24 | 48 | 5.45 | -1.33 | -1.12 |
| NLTP | 24 | 59 | 6.89 | -1.33 | -1.12 |
| KRT81 | 2 | 54.9 | 5.47 | -1.34 | -1.13 |
| GBB1 | 7 | 37.4 | 6 | -1.34 | -1.13 |
| SQSTM | 13 | 47.7 | 5.22 | -1.37 | -1.16 |
| CLMP | 5 | 41.3 | 7.99 | -1.39 | -1.18 |
| CYTSB | 17 | 118.5 | 6.7 | -1.42 | -1.21 |
| PSB10 | 3 | 28.9 | 7.81 | -1.42 | -1.21 |
| GLMP | 2 | 43.8 | 6.58 | -1.45 | -1.24 |
| TIMP3 | 8 | 24.1 | 8.72 | -1.45 | -1.24 |
| SMD1 | 5 | 13.3 | 11.56 | -1.55 | -1.34 |
| S38A2 | 7 | 56 | 8 | -1.57 | -1.36 |
| RB27B | 7 | 24.6 | 5.52 | -1.59 | -1.38 |
| ICAM1 | 16 | 57.8 | 7.99 | -1.6 | -1.39 |
| PIGU | 6 | 50 | 7.72 | -1.61 | -1.4 |
| NMES1 | 7 | 9.6 | 9.47 | -1.63 | -1.42 |
| IL6 | 4 | 23.7 | 6.57 | -1.68 | -1.47 |
| P2RX4 | 3 | 43.3 | 7.99 | -1.7 | -1.49 |
| A2MG | 5 | 163.2 | 6.46 | -1.72 | -1.51 |
| CP1B1 | 8 | 60.8 | 8.98 | -1.75 | -1.54 |
| NPC1 | 22 | 142.1 | 5.36 | -1.76 | -1.55 |
| GDF15 | 4 | 34.1 | 9.66 | -1.86 | -1.65 |
| PTX3 | 12 | 41.9 | 5.01 | -1.95 | -1.74 |
| S10A4 | 5 | 11.7 | 6.11 | -2.07 | -1.86 |
| DRA | 2 | 28.6 | 5 | -2.09 | -1.88 |
| TF | 9 | 33 | 7.03 | -2.18 | -1.97 |
| S10A2 | 2 | 11.1 | 4.78 | -2.27 | -2.06 |
| CYTN | 3 | 16.4 | 7.21 | -2.37 | -2.16 |
| SDC2 | 8 | 22.1 | 4.86 | -2.4 | -2.19 |
| DHB2 | 2 | 42.8 | 8.5 | -2.68 | -2.47 |

* Common genes in IV2 and LC list.

**Additional Table S4. Full list of the iTRAQ-generated IV2 membrane list**

| **Gene symbol** | **# Unique Peptides** | **MW [kDa]** | **calc. pI** | **Abundance Ratio (log2):IV2/231** | **NorLog2 IV2/231** |
| --- | --- | --- | --- | --- | --- |
| ENG* | 3 | 70.5 | 6.61 | 1.46 | 1.61 |
| SERA | 7 | 56.6 | 6.71 | 1.39 | 1.54 |
| LAPM5 | 3 | 29.9 | 8.68 | 1.38 | 1.53 |
| APCDL | 2 | 55.6 | 8.85 | 1.3 | 1.45 |
| CLC2B | 2 | 17.3 | 8.75 | 1.19 | 1.34 |
| TNR16 | 4 | 45.2 | 4.7 | 1.18 | 1.33 |
| PAI2 | 2 | 46.6 | 5.63 | 1.15 | 1.3 |
| ABCG2 | 2 | 72.3 | 8.69 | 1.07 | 1.22 |
| S35A2 | 3 | 41.3 | 9.96 | 0.97 | 1.12 |
| ACS2L | 12 | 74.8 | 7.11 | 0.96 | 1.11 |
| S12A7 | 9 | 119 | 6.71 | 0.95 | 1.1 |
| PHLA2 | 4 | 17.1 | 9.17 | 0.94 | 1.09 |
| SREBF2* | 4 | 123.6 | 8.4 | 0.92 | 1.07 |
| DC8L2 | 2 | 71.1 | 4.73 | 0.91 | 1.06 |
| TCEA3 | 2 | 38.9 | 9.19 | 0.88 | 1.03 |
| GFPT2 | 13 | 76.9 | 7.37 | 0.88 | 1.03 |
| TM163 | 2 | 31.4 | 7.53 | 0.87 | 1.02 |
| LPCAT2* | 8 | 60.2 | 6.55 | 0.86 | 1.01 |
| NAT14 | 2 | 21.6 | 10.74 | 0.86 | 1.01 |
| SVIP | 3 | 8.4 | 8.91 | 0.78 | 0.93 |
| ANT3 | 2 | 52.6 | 6.71 | 0.78 | 0.93 |
| RTN2* | 5 | 59.2 | 5.31 | 0.74 | 0.89 |
| SPNXB | 3 | 11.8 | 6.15 | 0.74 | 0.89 |
| MRC2 | 14 | 166.6 | 5.83 | 0.73 | 0.88 |
| HMHA1 | 2 | 124.5 | 6.1 | 0.73 | 0.88 |
| PDE1C | 3 | 80.7 | 8.82 | 0.72 | 0.87 |
| GDIR2 | 10 | 23 | 5.21 | 0.72 | 0.87 |
| KKLC1 | 4 | 12.8 | 10.2 | 0.71 | 0.86 |
| SLIK3 | 3 | 108.9 | 7.37 | 0.71 | 0.86 |
| CNTP3 | 5 | 140.6 | 7.78 | 0.7 | 0.85 |
| FUBP1 | 23 | 67.5 | 7.61 | 0.7 | 0.85 |
| RPL26L1* | 3 | 17.2 | 10.55 | 0.69 | 0.84 |
| GSH0 | 6 | 30.7 | 6.02 | 0.69 | 0.84 |
| AFAP1 | 8 | 80.7 | 8.68 | 0.67 | 0.82 |
| CEP41 | 3 | 41.3 | 8.32 | 0.67 | 0.82 |
| UBP16 | 4 | 93.5 | 6.93 | 0.67 | 0.82 |
| ERCC2 | 6 | 86.9 | 7.15 | 0.66 | 0.81 |
| H1X | 3 | 22.5 | 10.76 | 0.66 | 0.81 |
| TALDO | 17 | 37.5 | 6.81 | 0.66 | 0.81 |
| EREG* | 2 | 19 | 7.53 | 0.65 | 0.8 |
| TSYL2 | 2 | 79.4 | 4.58 | 0.65 | 0.8 |
| LENG8 | 6 | 86.1 | 9.35 | 0.65 | 0.8 |
| NUCG | 6 | 32.6 | 9.5 | 0.65 | 0.8 |
| P53 | 11 | 43.6 | 6.79 | 0.65 | 0.8 |
| DPH1 | 3 | 48.8 | 8.18 | 0.65 | 0.8 |
| CHD8 | 4 | 290.3 | 6.47 | 0.64 | 0.79 |
| TRAF1 | 3 | 46.1 | 6.11 | 0.64 | 0.79 |
| NHEJ1 | 2 | 33.3 | 5.97 | 0.64 | 0.79 |
| HMGN1* | 2 | 10.7 | 9.6 | 0.63 | 0.78 |
| EDF1 | 7 | 16.4 | 9.95 | 0.63 | 0.78 |
| RRFM | 7 | 29.3 | 9.79 | 0.63 | 0.78 |
| ASB6 | 2 | 47.1 | 6.01 | 0.63 | 0.78 |
| RMI1 | 2 | 70.1 | 4.96 | 0.63 | 0.78 |
| MTNA | 3 | 39.1 | 6.3 | 0.63 | 0.78 |
| KCAB2 | 5 | 41 | 9 | 0.63 | 0.78 |
| IDHC | 13 | 46.6 | 7.01 | 0.62 | 0.77 |
| SIPA1 | 4 | 112.1 | 6.6 | 0.61 | 0.76 |
| WLS | 8 | 62.2 | 7.36 | 0.61 | 0.76 |
| TNFL9 | 2 | 26.6 | 7.02 | 0.61 | 0.76 |
| SIA4C | 3 | 38 | 9.41 | 0.6 | 0.75 |
| RS30 | 2 | 6.6 | 12.15 | 0.6 | 0.75 |
| KAT1 | 4 | 47.8 | 6.47 | 0.6 | 0.75 |
| FHL1 | 4 | 36.2 | 8.97 | 0.6 | 0.75 |
| PLXD1 | 2 | 211.9 | 7.15 | 0.6 | 0.75 |
| CHPT1 | 2 | 45.1 | 6.92 | 0.59 | 0.74 |
| ANXA1 | 21 | 38.7 | 7.02 | 0.59 | 0.74 |
| PTMA | 7 | 12.2 | 3.78 | 0.58 | 0.73 |
| SEM3A | 4 | 88.8 | 7.42 | 0.57 | 0.72 |
| CISD1 | 5 | 12.2 | 9.09 | 0.57 | 0.72 |
| BMI1 | 2 | 36.9 | 8.63 | 0.56 | 0.71 |
| GSE1 | 3 | 136.1 | 7.74 | 0.56 | 0.71 |
| NC2A | 4 | 22.3 | 5.17 | 0.56 | 0.71 |
| ITB3 | 4 | 87 | 5.24 | 0.56 | 0.71 |
| NUDT4 | 3 | 20.3 | 6.35 | 0.56 | 0.71 |
| SPF30 | 5 | 26.7 | 7.24 | 0.56 | 0.71 |
| CO6A2 | 3 | 108.5 | 6.21 | 0.56 | 0.71 |
| CSF1 | 14 | 60.1 | 5.29 | 0.56 | 0.71 |
| SMRD2 | 2 | 58.9 | 9.64 | 0.55 | 0.7 |
| RUSC2 | 3 | 161.1 | 6.62 | 0.55 | 0.7 |
| SACS | 6 | 520.8 | 7.05 | 0.55 | 0.7 |
| RL35 | 4 | 14.5 | 11.05 | 0.55 | 0.7 |
| RNF4 | 3 | 21.3 | 7.03 | 0.55 | 0.7 |
| RBFA | 3 | 38.3 | 7.85 | 0.55 | 0.7 |
| DHB2 | 2 | 42.8 | 8.5 | -2.44 | -2.29 |
| S10A2 | 2 | 11.1 | 4.78 | -2.31 | -2.16 |
| A2MG | 5 | 163.2 | 6.46 | -2.1 | -1.95 |
| DRA | 2 | 28.6 | 5 | -1.96 | -1.81 |
| SPX2 | 4 | 54.4 | 6.81 | -1.83 | -1.68 |
| CLN8 | 2 | 32.8 | 8.21 | -1.83 | -1.68 |
| RFFL | 4 | 40.5 | 5.48 | -1.8 | -1.65 |
| KCRB | 11 | 42.6 | 5.59 | -1.79 | -1.64 |
| TF | 9 | 33 | 7.03 | -1.78 | -1.63 |
| IMUP | 2 | 10.9 | 9.73 | -1.75 | -1.6 |
| S10A6 | 3 | 10.2 | 5.48 | -1.67 | -1.52 |
| PPB1 | 18 | 57.9 | 6.29 | -1.59 | -1.44 |
| PLS3 | 3 | 31.6 | 6.65 | -1.54 | -1.39 |
| F133A | 2 | 28.9 | 10.1 | -1.52 | -1.37 |
| TACD2 | 10 | 35.7 | 8.87 | -1.47 | -1.32 |
| PSG9 | 3 | 48.2 | 8.07 | -1.43 | -1.28 |
| ETHE1 | 12 | 27.9 | 6.83 | -1.43 | -1.28 |
| PKHA7 | 5 | 127.1 | 9.35 | -1.4 | -1.25 |
| PLD3 | 9 | 54.7 | 6.47 | -1.38 | -1.23 |
| HKDC1 | 7 | 102.5 | 7.12 | -1.37 | -1.22 |
| FOLR1 | 4 | 29.8 | 7.97 | -1.37 | -1.22 |
| PERP | 2 | 21.4 | 7.03 | -1.34 | -1.19 |
| RALA | 7 | 23.6 | 7.11 | -1.33 | -1.18 |
| PODXL | 11 | 58.6 | 5.49 | -1.32 | -1.17 |
| TM256 | 4 | 11.7 | 8.94 | -1.31 | -1.16 |
| CP1B1 | 8 | 60.8 | 8.98 | -1.3 | -1.15 |
| GBG5 | 3 | 7.3 | 9.85 | -1.3 | -1.15 |
| ECH1 | 16 | 35.8 | 8 | -1.27 | -1.12 |
| AT1A3 | 6 | 111.7 | 5.38 | -1.27 | -1.12 |
| EXD2 | 14 | 70.3 | 8.32 | -1.27 | -1.12 |
| PTTG | 4 | 20.3 | 8.79 | -1.26 | -1.11 |
| GDF15 | 4 | 34.1 | 9.66 | -1.24 | -1.09 |
| D39U1 | 5 | 34.7 | 9.77 | -1.24 | -1.09 |
| IL6 | 4 | 23.7 | 6.57 | -1.22 | -1.07 |
| CSPG4 | 21 | 250.4 | 5.47 | -1.21 | -1.06 |
| UBP11 | 19 | 109.7 | 5.45 | -1.2 | -1.05 |
| KRT81 | 2 | 54.9 | 5.47 | -1.18 | -1.03 |
| RB27B | 7 | 24.6 | 5.52 | -1.17 | -1.02 |
| GNAS1 | 15 | 111 | 5.03 | -1.17 | -1.02 |
| AL4A1 | 18 | 61.7 | 8.07 | -1.17 | -1.02 |
| OAS2 | 2 | 82.4 | 8.25 | -1.16 | -1.01 |
| CSF2R | 7 | 46.2 | 7.75 | -1.16 | -1.01 |
| GLMP | 2 | 43.8 | 6.58 | -1.14 | -0.99 |
| T161B | 4 | 55.4 | 8.37 | -1.13 | -0.98 |
| ARF6 | 5 | 20.1 | 8.95 | -1.13 | -0.98 |
| AOFA | 21 | 59.6 | 7.85 | -1.13 | -0.98 |
| DTNB | 2 | 71.3 | 7.91 | -1.13 | -0.98 |
| CIRBP | 5 | 18.6 | 9.51 | -1.13 | -0.98 |
| PEN2 | 2 | 12 | 9.19 | -1.08 | -0.93 |
| ATIF1 | 5 | 12.2 | 9.35 | -1.08 | -0.93 |
| ISCA2 | 7 | 16.5 | 5.25 | -1.08 | -0.93 |
| CF132 | 2 | 124 | 9.45 | -1.07 | -0.92 |
| BCAT2 | 13 | 44.3 | 8.65 | -1.07 | -0.92 |
| SOAT1 | 13 | 64.7 | 8.94 | -1.06 | -0.91 |
| DNPH1 | 2 | 19.1 | 5.05 | -1.06 | -0.91 |
| RAB23 | 14 | 26.6 | 6.6 | -1.06 | -0.91 |
| SYNEM | 2 | 172.7 | 5.16 | -1.06 | -0.91 |
| RAB13 | 8 | 22.8 | 9.19 | -1.05 | -0.9 |
| TM38A | 2 | 33.2 | 8.29 | -1.04 | -0.89 |
| SGPP1 | 12 | 49.1 | 8.82 | -1.04 | -0.89 |
| MYCBP | 6 | 12 | 5.91 | -1.03 | -0.88 |
| RB11B | 2 | 24.5 | 5.94 | -1.03 | -0.88 |
| MOCS1 | 9 | 70.1 | 9.26 | -1.02 | -0.87 |
| ARFG1 | 12 | 44.6 | 5.66 | -1.02 | -0.87 |
| PPAL | 11 | 48.3 | 6.74 | -1.02 | -0.87 |
| F210B | 4 | 20.4 | 10.43 | -1.02 | -0.87 |
| GLRX5 | 6 | 16.6 | 6.79 | -1.02 | -0.87 |
| GTR1 | 8 | 54 | 8.72 | -1.01 | -0.86 |
| GP108 | 7 | 60.6 | 8.69 | -1.01 | -0.86 |
| PRAF1 | 3 | 20.6 | 7.34 | -1.01 | -0.86 |
| RHOA | 3 | 21.8 | 6.1 | -1 | -0.85 |
| E41L5 | 7 | 81.8 | 6.58 | -1 | -0.85 |
| SEM7A | 19 | 74.8 | 7.64 | -1 | -0.85 |
| ATP5J | 5 | 12.6 | 9.52 | -0.99 | -0.84 |
| COX5A | 8 | 16.8 | 6.79 | -0.98 | -0.83 |
| F195B | 2 | 10.9 | 9.41 | -0.98 | -0.83 |
| RHOB | 3 | 22.1 | 5.24 | -0.98 | -0.83 |
| ATP9B | 5 | 129.2 | 7.61 | -0.98 | -0.83 |
| COA4 | 5 | 10.1 | 6.04 | -0.98 | -0.83 |
| DYSF | 64 | 237.1 | 5.64 | -0.97 | -0.82 |
| BDH | 13 | 38.1 | 8.95 | -0.97 | -0.82 |
| NAAA | 5 | 40 | 8.75 | -0.97 | -0.82 |
| S39AB | 4 | 35.4 | 5.6 | -0.96 | -0.81 |
| ZN428 | 4 | 20.5 | 4.17 | -0.96 | -0.81 |
| NOC2L | 7 | 84.9 | 5.62 | -0.96 | -0.81 |
| TMED9 | 6 | 27.3 | 8.02 | -0.96 | -0.81 |
| LEG3 | 11 | 26.1 | 8.56 | -0.95 | -0.8 |
| MYH10 | 28 | 228.9 | 5.54 | -0.95 | -0.8 |
| SYSM | 21 | 58.2 | 8.13 | -0.94 | -0.79 |
| DPY30 | 4 | 11.2 | 4.88 | -0.94 | -0.79 |
| PLS1 | 4 | 35 | 4.94 | -0.94 | -0.79 |
| NDUF3 | 5 | 20.3 | 8.22 | -0.94 | -0.79 |
| TSN6 | 7 | 27.5 | 8.1 | -0.94 | -0.79 |
| SPTC2 | 29 | 62.9 | 7.78 | -0.94 | -0.79 |
| LSR | 9 | 71.4 | 7.97 | -0.94 | -0.79 |
| HXK2 | 39 | 102.3 | 6.05 | -0.94 | -0.79 |
| ACOT2 | 2 | 53.2 | 8.47 | -0.94 | -0.79 |
| HMGB3 | 6 | 23 | 8.37 | -0.93 | -0.78 |
| CCRL2 | 3 | 39.5 | 7.77 | -0.93 | -0.78 |
| SH3L3 | 4 | 10.4 | 4.93 | -0.93 | -0.78 |
| PPOX | 14 | 50.7 | 8.16 | -0.93 | -0.78 |
| PLPP3 | 5 | 35.1 | 9.14 | -0.93 | -0.78 |
| TSN13 | 3 | 22.1 | 7.93 | -0.93 | -0.78 |
| MSPD1 | 3 | 24.1 | 7.78 | -0.93 | -0.78 |
| TIM10 | 6 | 10.3 | 6.29 | -0.93 | -0.78 |
| RON | 2 | 152.2 | 6.55 | -0.93 | -0.78 |
| BOLA3 | 5 | 12.1 | 9.64 | -0.93 | -0.78 |
| PXMP4 | 3 | 24.2 | 10.07 | -0.93 | -0.78 |
| SNX21 | 3 | 41.3 | 5.24 | -0.93 | -0.78 |
| DAP1 | 2 | 11.2 | 9.32 | -0.93 | -0.78 |
| SMD1 | 5 | 13.3 | 11.56 | -0.92 | -0.77 |
| KAP2 | 18 | 45.5 | 5.07 | -0.92 | -0.77 |
| DNSL1 | 5 | 33.9 | 5.74 | -0.92 | -0.77 |
| LTOR1 | 6 | 17.7 | 5.15 | -0.92 | -0.77 |
| YIPF2 | 3 | 35.1 | 5.73 | -0.92 | -0.77 |
| ACYP1 | 2 | 11.3 | 9.31 | -0.91 | -0.76 |
| KPCD | 24 | 77.5 | 7.75 | -0.91 | -0.76 |
| UBA1 | 42 | 117.8 | 5.76 | -0.91 | -0.76 |
| MFGM | 21 | 43.1 | 8.15 | -0.91 | -0.76 |
| ES1 | 9 | 28.2 | 8.27 | -0.91 | -0.76 |
| PGH2 | 16 | 69 | 7.39 | -0.91 | -0.76 |
| ENDD1 | 13 | 55 | 5.71 | -0.9 | -0.75 |
| MGST3 | 5 | 16.5 | 9.38 | -0.9 | -0.75 |
| RAB9B | 3 | 22.7 | 4.87 | -0.9 | -0.75 |
| GNAI1 | 3 | 40.3 | 5.97 | -0.9 | -0.75 |
| FKBP8 | 13 | 44.5 | 4.84 | -0.9 | -0.75 |
| THIOM | 5 | 18.4 | 8.29 | -0.9 | -0.75 |
| PORCN | 3 | 52.3 | 8.84 | -0.9 | -0.75 |
| FUND1 | 2 | 17.2 | 8.62 | -0.9 | -0.75 |
| LTOR2 | 5 | 13.5 | 5.4 | -0.9 | -0.75 |
| SIDT2 | 3 | 94.4 | 6.96 | -0.9 | -0.75 |
| COF2 | 10 | 18.7 | 7.88 | -0.9 | -0.75 |
| SODM | 13 | 24.7 | 8.25 | -0.89 | -0.74 |
| TMEDA | 8 | 25 | 7.44 | -0.89 | -0.74 |
| LRP1 | 50 | 504.3 | 5.39 | -0.89 | -0.74 |
| TGM2 | 25 | 77.3 | 5.22 | -0.89 | -0.74 |
| GALT6 | 8 | 71.1 | 8.18 | -0.89 | -0.74 |
| RCN3 | 5 | 37.5 | 4.89 | -0.89 | -0.74 |
| K1C18 | 24 | 48 | 5.45 | -0.88 | -0.73 |
| SCMC1 | 23 | 53.3 | 6.33 | -0.88 | -0.73 |
| GAS6 | 13 | 79.6 | 6.21 | -0.88 | -0.73 |
| AIG1 | 4 | 28.2 | 8.25 | -0.88 | -0.73 |
| PYGL | 26 | 97.1 | 7.17 | -0.88 | -0.73 |
| TMED3 | 4 | 24.8 | 5.6 | -0.88 | -0.73 |
| SDCB1 | 8 | 32.4 | 7.53 | -0.87 | -0.72 |
| PLAK | 15 | 81.7 | 6.14 | -0.87 | -0.72 |
| PLCD3 | 16 | 89.2 | 6.98 | -0.87 | -0.72 |
| SSBP | 9 | 17.2 | 9.6 | -0.87 | -0.72 |
| PLCA | 8 | 31.7 | 9.38 | -0.87 | -0.72 |
| CMC4 | 4 | 7.7 | 8.18 | -0.87 | -0.72 |
| RAI3 | 7 | 40.2 | 8.15 | -0.87 | -0.72 |
| ODO2 | 15 | 48.7 | 8.95 | -0.87 | -0.72 |
| VIME | 42 | 53.6 | 5.12 | -0.86 | -0.71 |
| PLD1 | 17 | 124.1 | 8.78 | -0.86 | -0.71 |
| CTNB1 | 25 | 85.4 | 5.86 | -0.86 | -0.71 |
| TMED1 | 4 | 25.2 | 4.48 | -0.86 | -0.71 |
| RAB30 | 6 | 23 | 4.97 | -0.86 | -0.71 |
| CREL1 | 6 | 45.4 | 4.87 | -0.86 | -0.71 |
| DNJC5 | 8 | 22.1 | 5.07 | -0.86 | -0.71 |
| PCKGM | 27 | 70.7 | 7.62 | -0.86 | -0.71 |
| K2C7 | 18 | 51.4 | 5.48 | -0.85 | -0.7 |
| CPPED | 3 | 35.5 | 6.2 | -0.85 | -0.7 |
| K2C8 | 30 | 53.7 | 5.59 | -0.85 | -0.7 |
| TMED7 | 9 | 25.2 | 6.89 | -0.85 | -0.7 |
| CCD94 | 8 | 37.1 | 5.92 | -0.85 | -0.7 |
| EMC9 | 4 | 23 | 5.91 | -0.85 | -0.7 |
| L2HDH | 17 | 50.3 | 8.15 | -0.85 | -0.7 |
| CHM4A | 6 | 25.1 | 4.7 | -0.85 | -0.7 |
| CQ062 | 7 | 20.8 | 6.8 | -0.85 | -0.7 |
| KEAP1 | 9 | 69.6 | 6.44 | -0.85 | -0.7 |
| MMSA | 24 | 57.8 | 8.5 | -0.85 | -0.7 |
| THIK | 16 | 44.3 | 8.44 | -0.85 | -0.7 |

* Common genes in IV2 and LC list.

**Additional Table S5. Reagents and antibodies**

| **Product** | **catalog no.** | **Species** | **Dilution** | **Company** |
| --- | --- | --- | --- | --- |
| **Antibodies** |  |  |  |  |
| anti-Na K ATPase α1 | #23565 | Rabbit | 1:1000, WB | Cell Signaling Technology |
| anti-Lamin B2 | #12255 | Rabbit | 1:5000, WB | Cell Signaling Technology |
| anti-SCEL | HPA040154 | Rabbit | 1:1000, WB | Sigma Aldrich |
| anti-Actin | sc-47778 | Rabbit | 1:5000, WB | Santa Cruz Biotechnology |
| Anti- α-Tubulin | #77763 | Rabbit | 1:5000, WB | Cell Signaling Technology |
| anti-p-p65 | #3033 | Rabbit | 1:1000, WB | Cell Signaling Technology |
| anti-p-65 | #8242 | Rabbit | 1:1000, WB | Cell Signaling Technology |
| anti-GAPDH | sc-32233 | Rabbit | 1:5000, WB | Santa Cruz Biotechnology |
| anti-FLIP | #56343 | Rabbit | 1:1000, WB | Cell Signaling Technology |
| anti-p-AKt | #4060 | Rabbit | 1:1000, WB | Cell Signaling Technology |
| anti-AKt | #9272 | Rabbit | 1:1000, WB | Cell Signaling Technology |
| anti-p-Erk 1/2 | #4370 | Rabbit | 1:1000, WB | Cell Signaling Technology |
| anti-Erk 1/2 | #9102 | Rabbit | 1:1000, WB | Cell Signaling Technology |
| anti-p-Bcl-2 | #2827 | Rabbit | 1:1000, WB | Cell Signaling Technology |
| anti-Bcl-2 | #3498 | Rabbit | 1:1000, WB | Cell Signaling Technology |
| anti-Cleaved Caspase 3 | #9661 | Rabbit | 1:1000, WB | Cell Signaling Technology |
| anti-TNFR1 | #3736 | Rabbit | 1:1000, WB | Cell Signaling Technology |
| anti-p-IGF-1R | #3024 | Rabbit | 1:1000, WB | Cell Signaling Technology |
| anti-IGF-1R | #3027 | Rabbit | 1:1000, WB | Cell Signaling Technology |
| anti-p-Met | #3077 | Rabbit | 1:1000, WB | Cell Signaling Technology |
| anti-Met | #8198 | Rabbit | 1:1000, WB | Cell Signaling Technology |
| anti-p-EGFR | #3777 | Rabbit | 1:1000, WB | Cell Signaling Technology |
| anti-EGFR | #2085 | Rabbit | 1:1000, WB | Cell Signaling Technology |
| anti-p-Stat 3 | #9145 | Rabbit | 1:1000, WB | Cell Signaling Technology |
| anti-CD68 | #26042 | Rabbit | 1:300, IHC | Cell Signaling Technology |
| anti-Ki67 | #9027 | Rabbit | 1:300, IHC | Cell Signaling Technology |
| anti-Stat 3 | #9139 | Rabbit | 1:1000, WB | Cell Signaling Technology |
| anti-CD206 | ab64693 | Rabbit | 1:300, IHC | Abcam |
| **Recombinant proteins** **and chemicals** | **catalog no.** | **Species** |  | **Company** |
| TNF-α | 210-TA | Human |  | R ＆ D |
| IGF-1 | 291-G1 | Human |  | R ＆ D |
| HGF | DHG00B | Human |  | R ＆ D |
| EGF | 236-EG | Human |  | R ＆ D |
| IL-6 | D6050 | Human |  | R ＆ D |
| Cyclohexamide | 1810 |  |  | Sigma Aldrich |
|  |  |  |  |  |
| **Tissue microarray** | **catalog no.** | **Species** |  | **Company** |
| TNBC TMA | BR489 | Human |  |  |
| TNBC TMA | BR1509 | Human |  |  |
| TNBC TMA | BR1902 | Human |  |  |

**Additional Table S6. ShRNAs and oligo primers**

| **Product** | **ID.** | **Sequences** |
| --- | --- | --- |
| SCEL shRNA -1 | TRCN0000158951 | CCGGGCTACTGAAGTAAATCCCAAACTCGAGTTTGGGATTTACTTCAGTAGCTTTTTTG |
| SCEL shRNA -2 | TRCN0000159937 | CCGGGATCTTGCTAACCTCATCAAACTCGAGTTTGATGAGGTTAGCAAGATCTTTTTTG |
| SCEL shRNA -3 | TRCN0000160772 | CCGGCCACCTAAACCAGGTGTATATCTCGAGATATACACCTGGTTTAGGTGGTTTTTTG |
| IER3 | NM_003897 | F-CTCAGCACTTTCCTCCAGCA  R-CGCCTGGTGTTTCTTTGTGG |
| TGM2 | NM_004613 | F- ATTCCCTCTCCTGCCCAGAT  R-AGGGAGCTGGATTCCCTGAT |
| CFLAR | NM_003879 | F-GAGTGCCGGCTATTGGACTT  R-GCGCTTCTCTCCTACACCTC |
| CCND1 | NM_053056 | F-TGTCCTACTACCGCCTCACA  R-CTTGGGGTCCATGTTCTGCT |
| MYC | NM_002467 | F-CCCTCCACTCGGAAGGACTA R-GCTGGTGCATTTTCGGTTGT |
| ACTN | NM_001101 | F-CCTCGCCTTTGCCGATCC  R-CGCGGCGATATCATCATCC |

**Additional methods**

**Tandem mass spectrometry-based protein identification.**

Tandem mass spectrometry analysis was performed on Q Exactive^TM^ HF mass spectrometer (Thermo Fisher, San Jose) coupled with a Thermo Scientific™ UltiMate™ 3000 RSLCnano HPLC System. The peptide mixtures are directly loaded onto a 50-cm analytic column (EASY-Spray™ C18 Column), and separated by a gradient with gradually increased of buffer B (80 % acetonitrile in 0.1 % formic acid) at a flow rate of 250 nL/min over about 165 min. The peptide spectra were acquired in positive ion mode with a data-dependent acquisition. The top abundant fifteen precursor ions within 375-1400 m/z scan range were dynamically selected for further fragmented in high collision dissociation (HCD) mode with normalized collision energy set to 33±1. In Full MS scan, the resolution was set to 60000 at m/z 200, AGC target to 3e6, maximum inject time to 50 ms. In MS/MS scan, the resolution was set to 15000, AGC target to 5e4, maximum inject time to 100 ms. The release of dynamic exclusion of the selected precursor ions was set to 20 sec.

**Analysis of MS-generated proteomic data by Proteome Discoverer software and Mascot search engine.**

The MS raw files are uploaded into Proteome Discoverer (version 2.1, Thermo Fisher Scientific, MA, USA) to generate peak list for the following protein identification analysis by using MASCOT search engine (version 2.5, Matrix Science, MA, USA) against non-redundant human protein sequences of SWISS-PROT database (released in Jan, 2016). For protein identification, carbamidomethylation at Cys as the fixed modification, oxidation at Met, Gln to pyro-Glu at peptide N-terminus, acetylation at protein *N*-terminus iTRAQ 4-plex-labeled at peptide N-terminus and K residue as dynamic modifications, maximum missing cleavage sites with 2, 10 ppm for MS tolerance and 0.02 Da for MS/MS tolerance are allowed for database searching. The peptide and protein identifications with false discovery rate less than 1% are accepted. To improve the confidence, the protein identification with at least two unique peptides and quantification with two spectrum ratio counts contributed from the unique peptides are considered. And then, the median value of the spectrum ratios is calculated as protein abundance. Finally, the global median normalization is applied to recalculate the protein abundance to generate the normalized protein ratios to reduce the system error from sample preparation in each experiment.
