# Supplementary material for: SCEL regulates switches between pro-survival and apoptosis of the TNF-α/TNFR1/NF-κB/c-FLIP axis to control lung colonization of triple negative breast cancer
Source: J Biomed Sci. 2023 Nov 30;30:93. doi: 10.1186/s12929-023-00986-4 (PMC10688137; doi:10.1186/s12929-023-00986-4)

**Additional Fig. S6.** Cell line authentication: Short tandem repeat (STR) analysis of 231-PT.


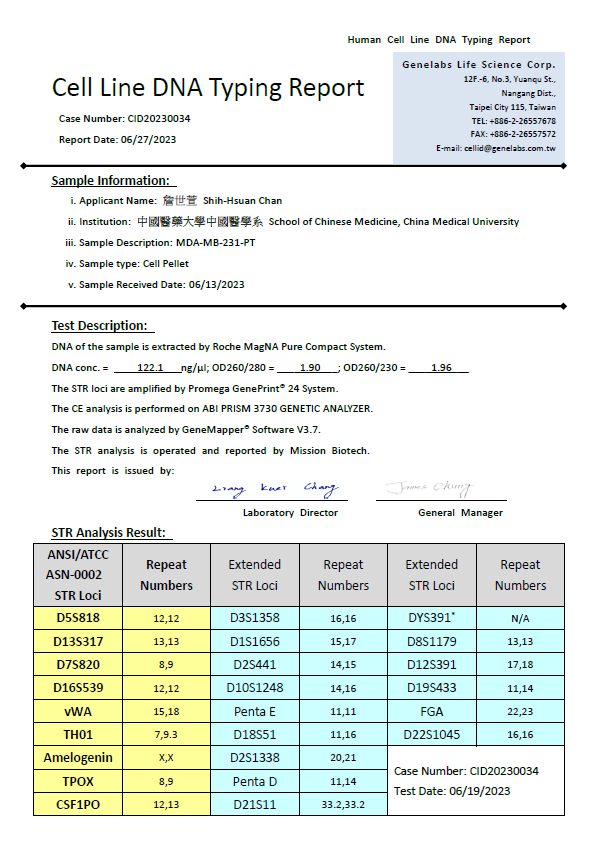


**Additional Fig. S6.** Cont.


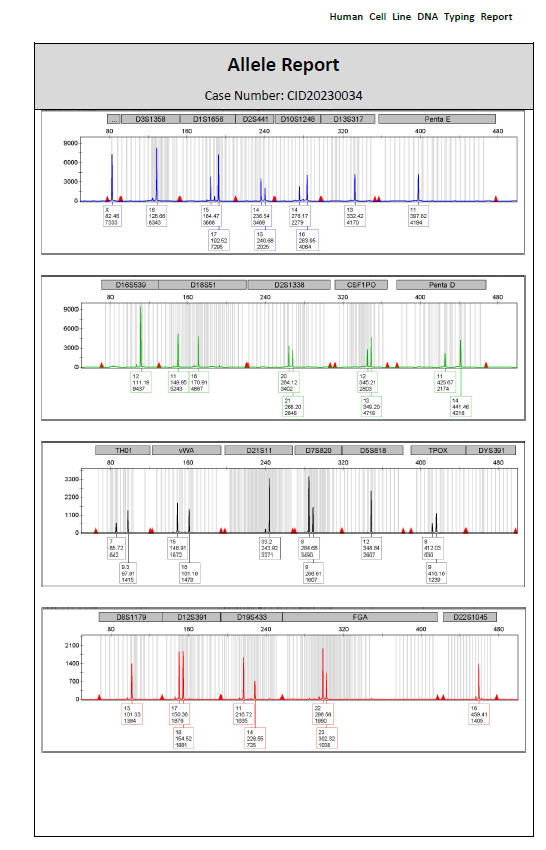


**Additional Fig. S6.** Cont.


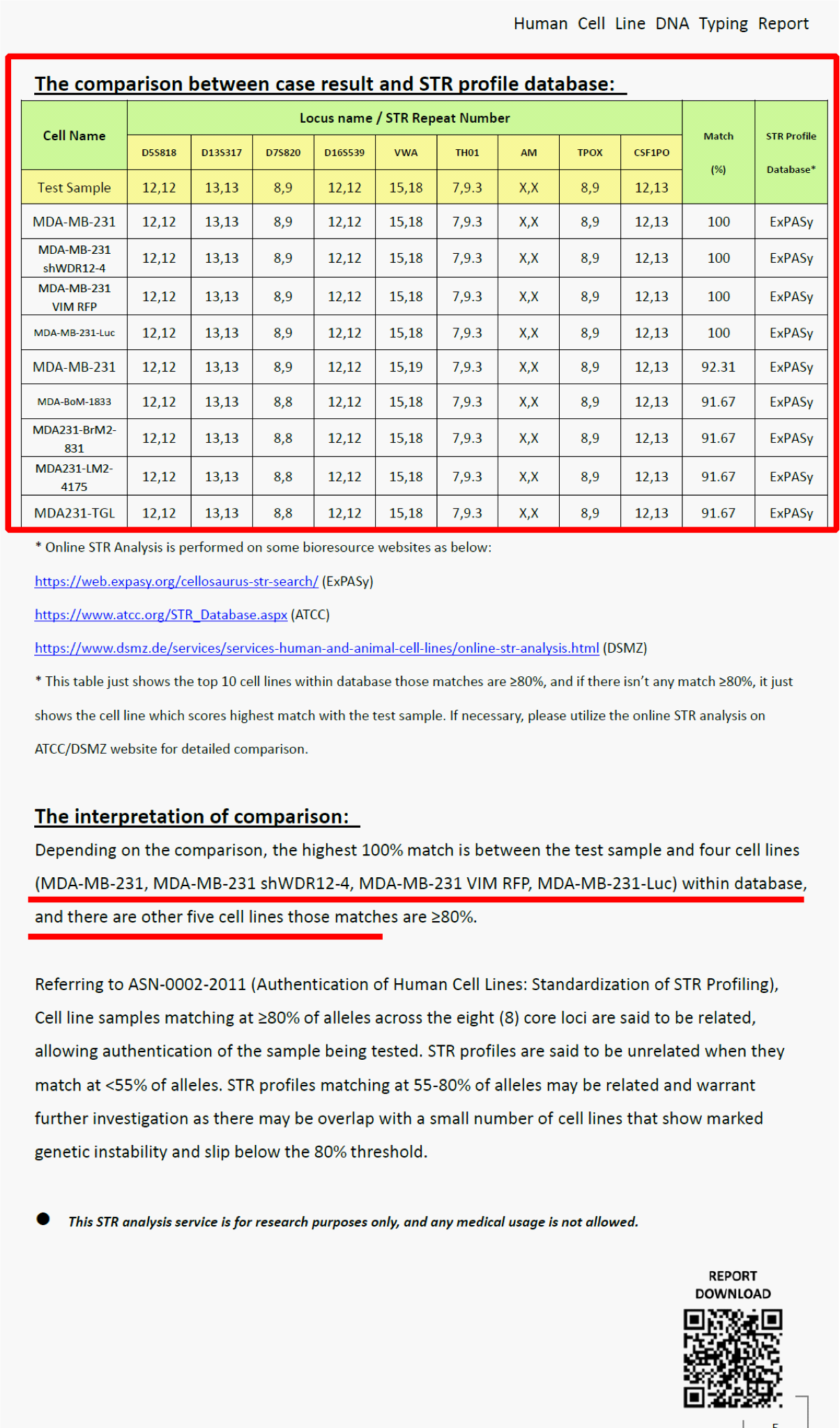


**Additional Fig. S6.** Cont.


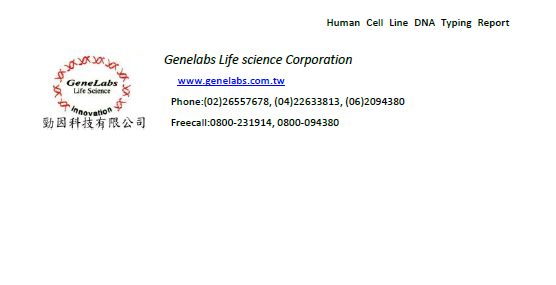


**Additional Fig. S7.** Cell line authentication: Short tandem repeat (STR) analysis of 231-LC.


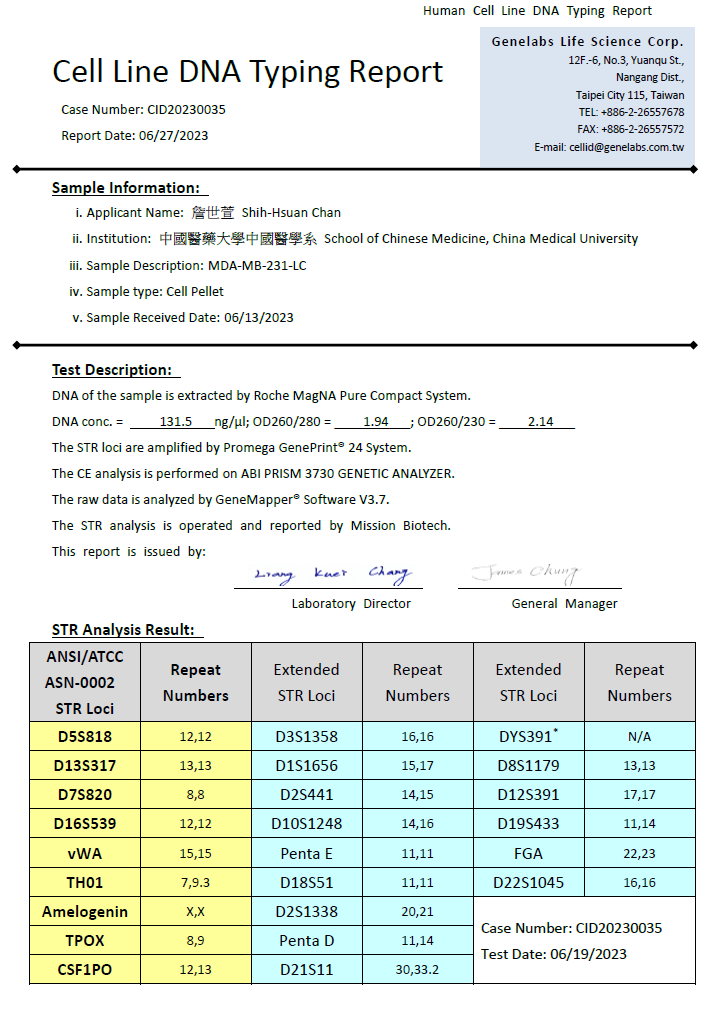


**Additional Fig. S7.** Cont.


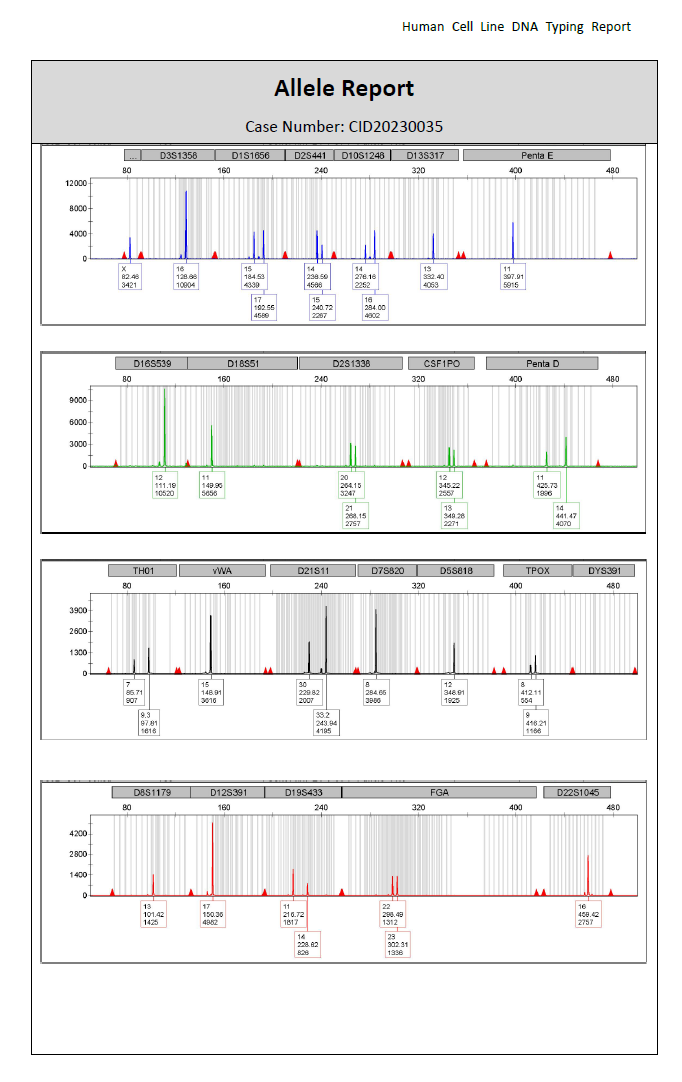


**Additional Fig. S7.** Cont.

©
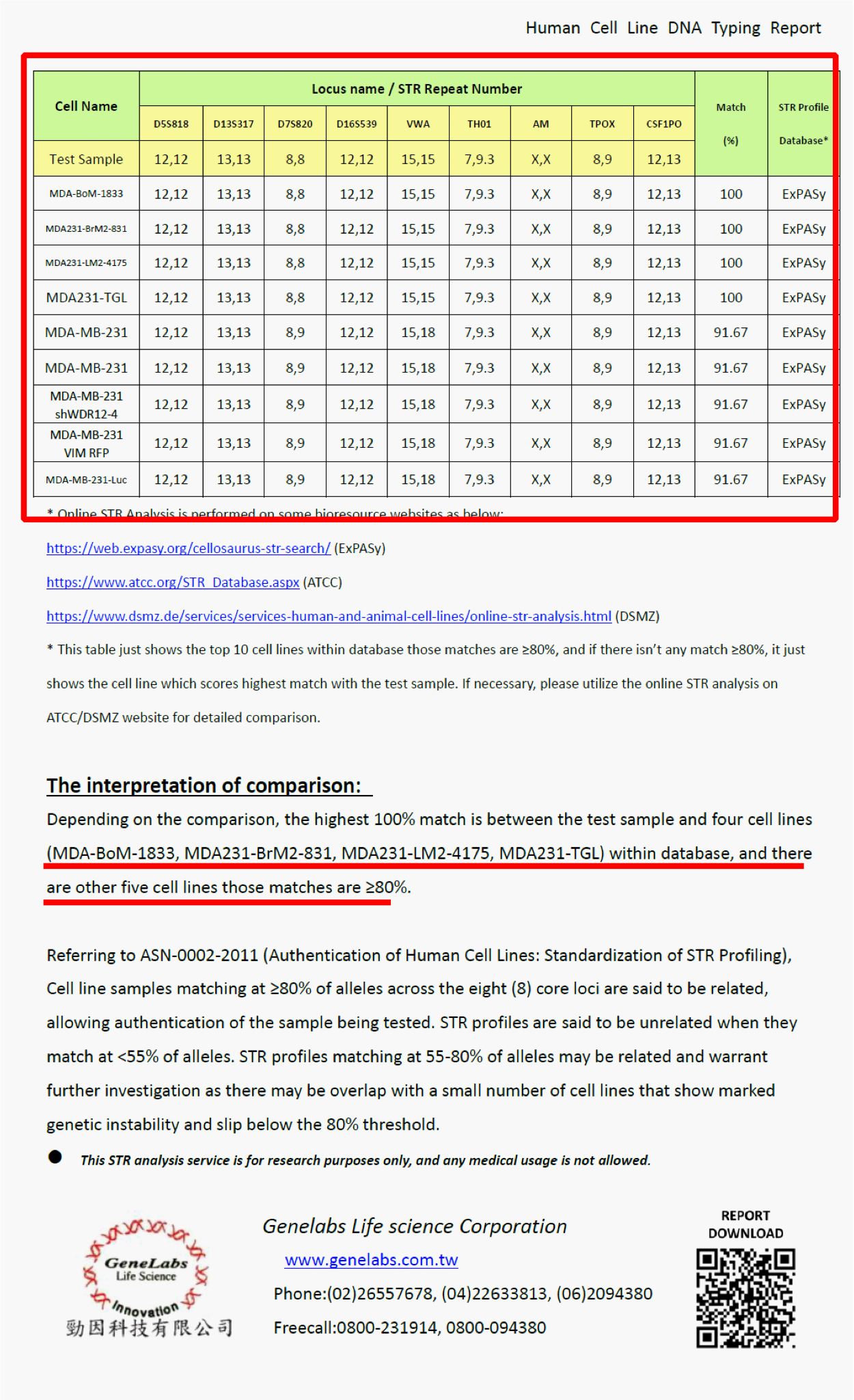


**Additional Fig. S8.** Cell line authentication: Short tandem repeat (STR) analysis of 231-IV2.


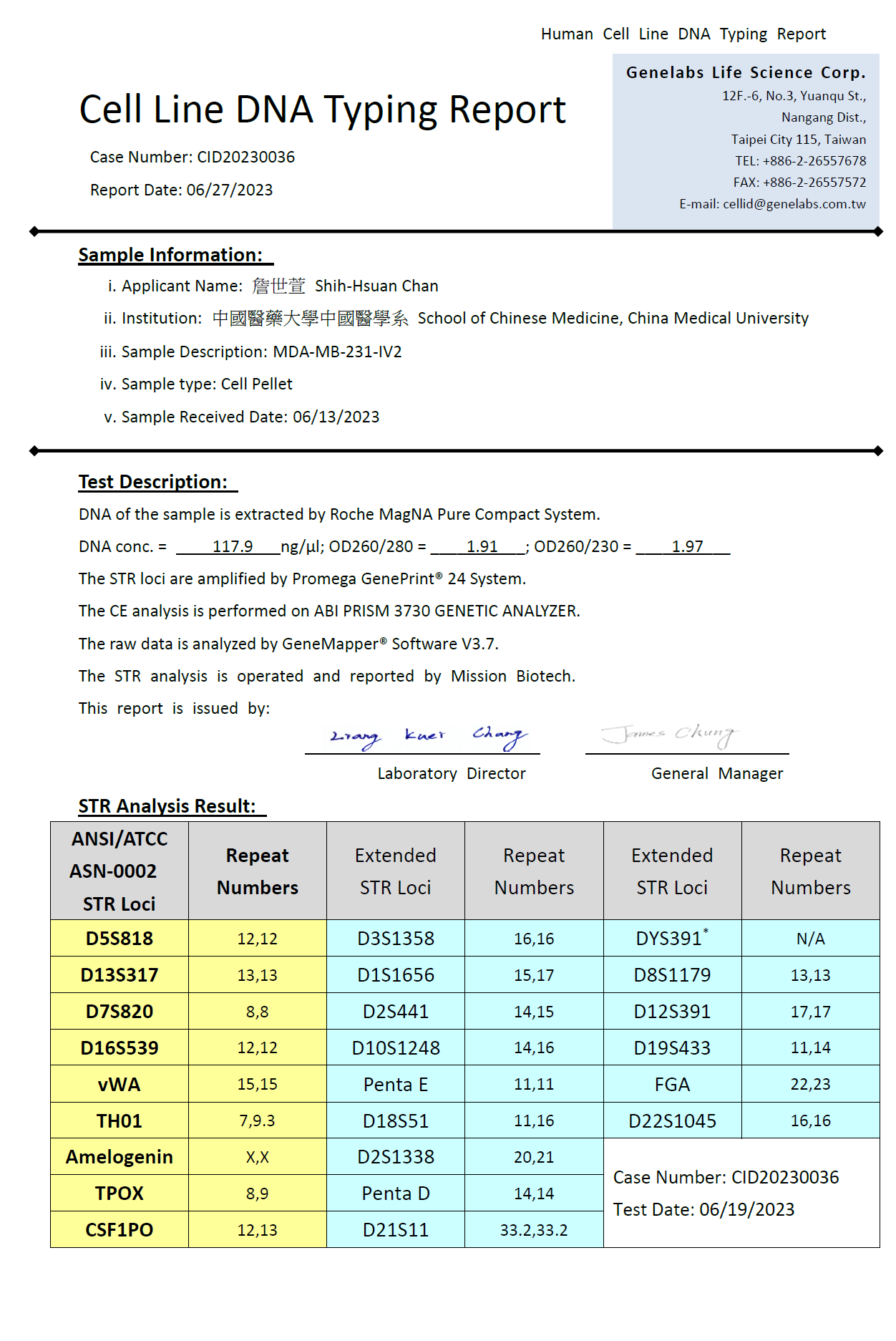


**Additional Fig. S8.** Cont.


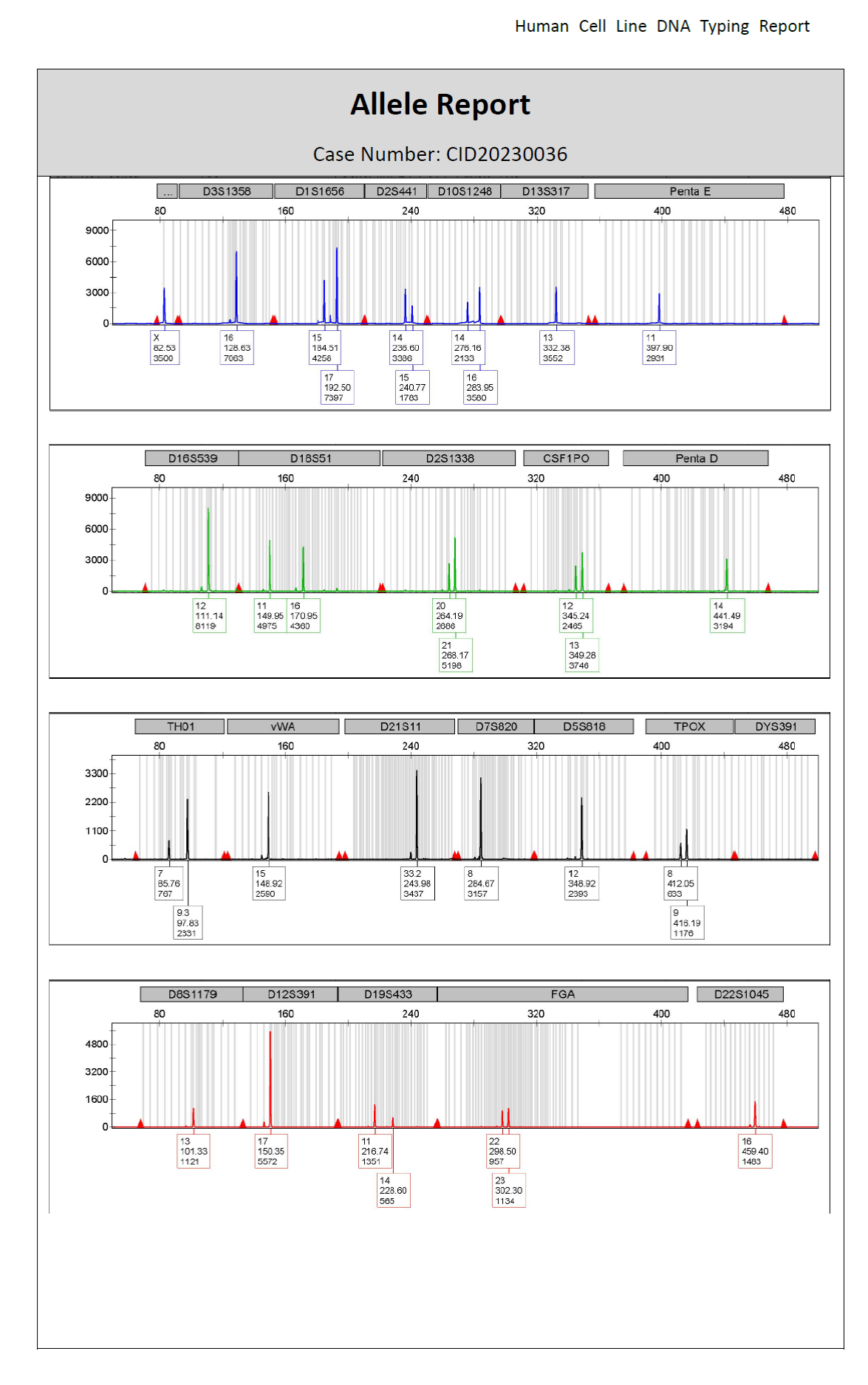


**Additional Fig. S8.** Cont.


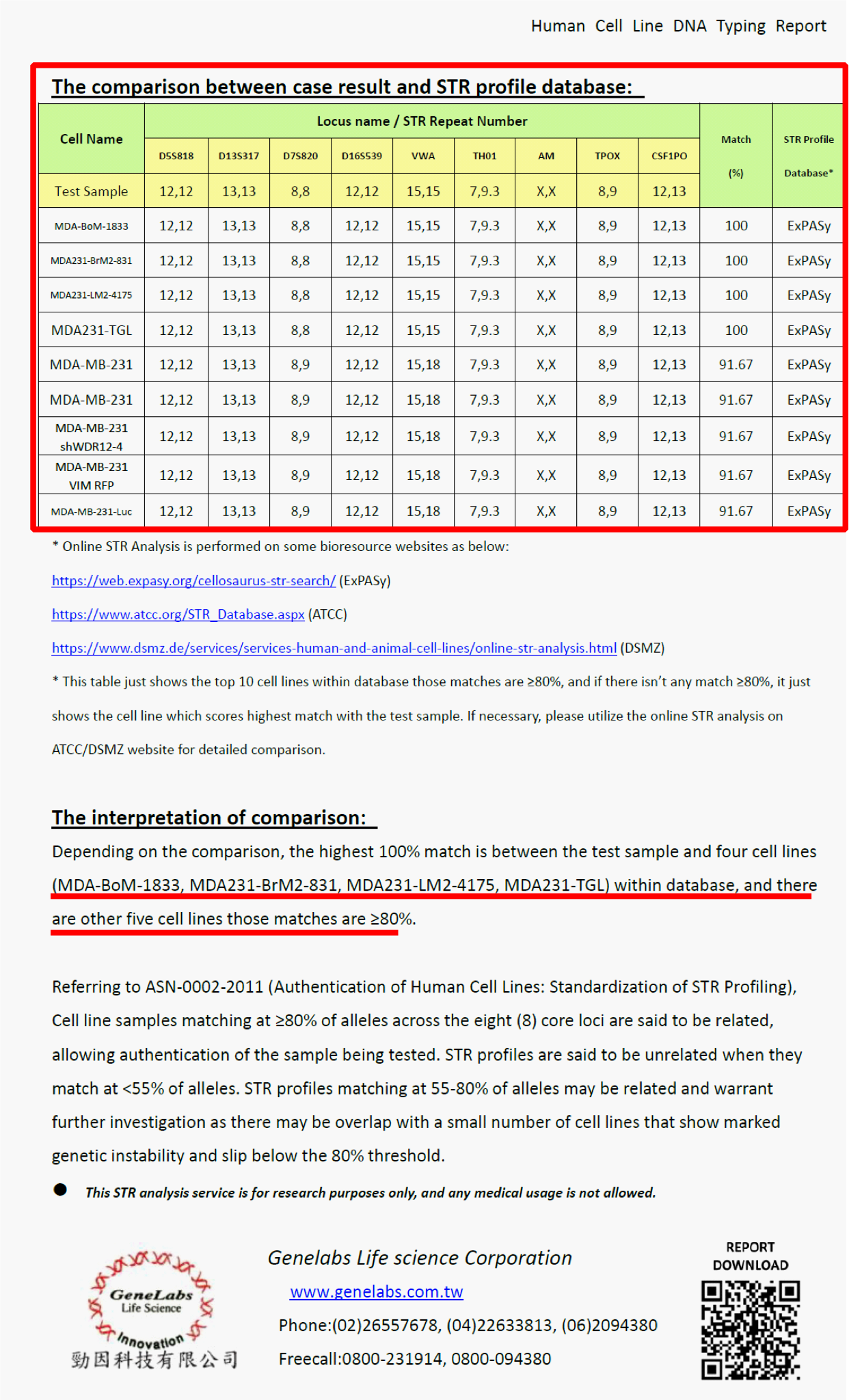


**Additional Fig. S9**. Cell line deposition at Bioresource Collection and Research Center (BCRC) at Food Industry Research and Development Institute, Hsinchu, Taiwan.


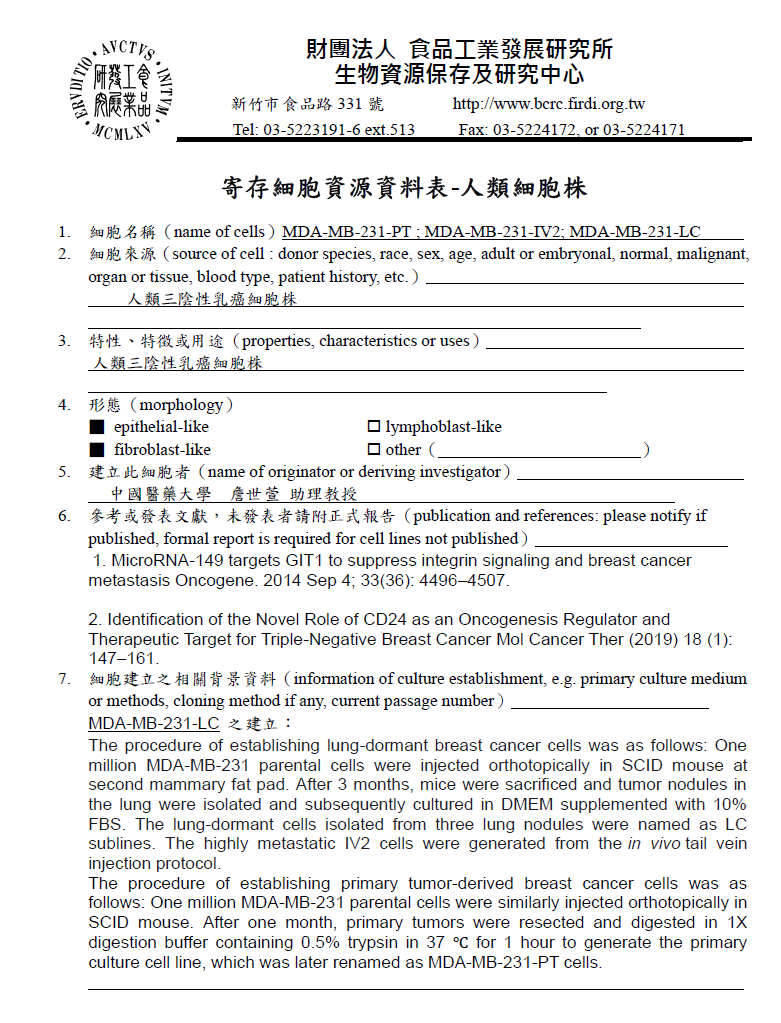


**Additional Fig. S9**. Cont.


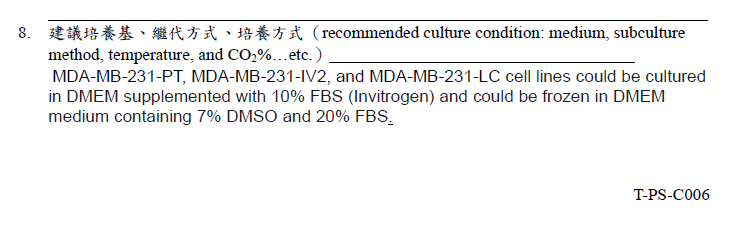


**Additional Fig. S9**. Cont.


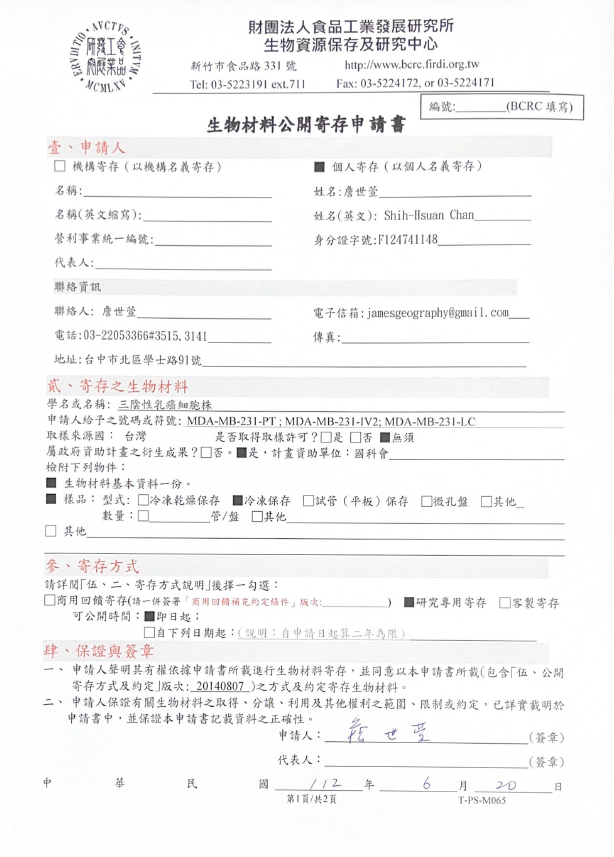


**Additional Fig. S9.** Cont.


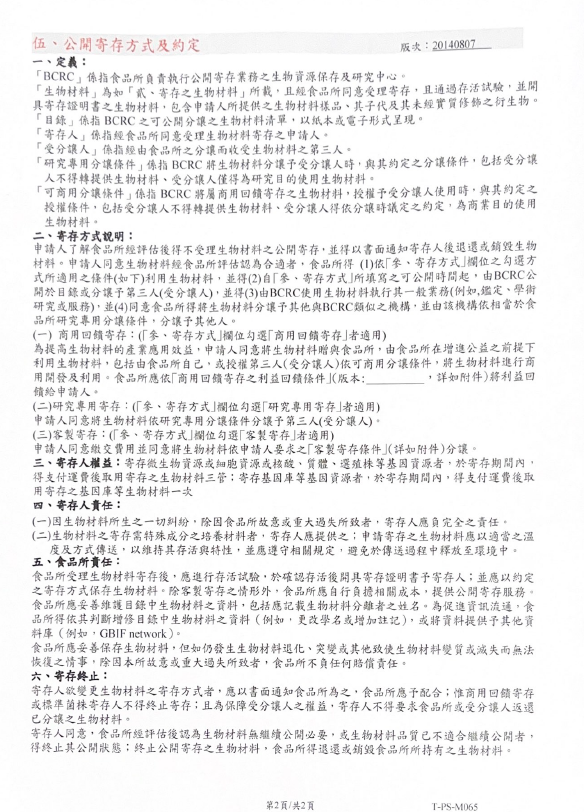


**Additional Fig. S9**. Cont.


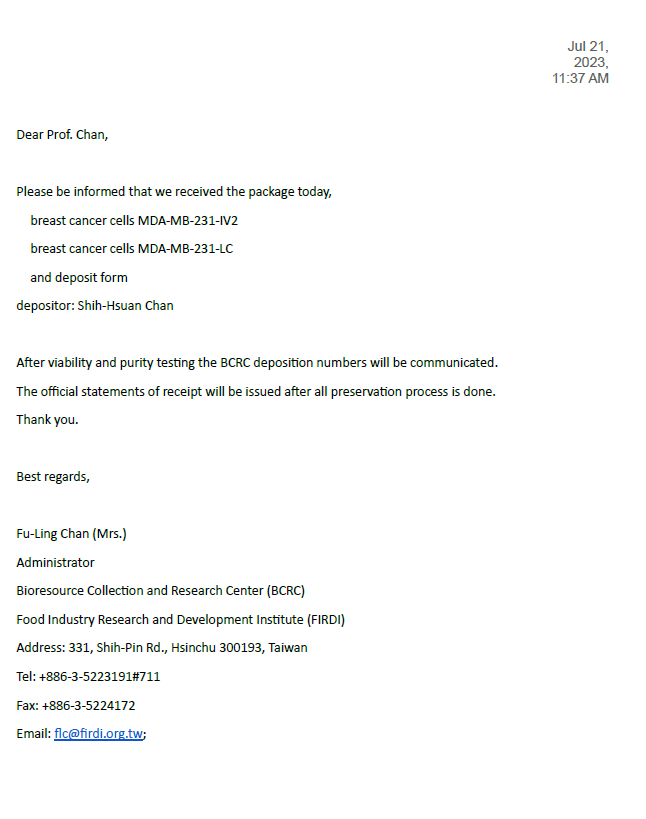

Supplement: Supplementary file 2 — Additional file 2: Fig. S6. Cell line authentication: Short tandem repeat (STR) analysis of 231-PT. Fig. S7. Cell line authentication: Short tandem repeat (STR) analysis of 231-LC. Fig. S8. Cell line authentication: Short tandem repeat (STR) analysis of 231-IV2. Fig. S9. Cell line deposition at Bioresource Collection and Research Center (BCRC) at Food Industry Research and Development Institute, Hsinchu, Taiwan. [file 12929_2023_986_MOESM2_ESM.docx]
