# Supplementary material for: SCEL regulates switches between pro-survival and apoptosis of the TNF-α/TNFR1/NF-κB/c-FLIP axis to control lung colonization of triple negative breast cancer
Source: J Biomed Sci. 2023 Nov 30;30:93. doi: 10.1186/s12929-023-00986-4 (PMC10688137; doi:10.1186/s12929-023-00986-4)

**Fig. S1. Uncropped Western blots.**


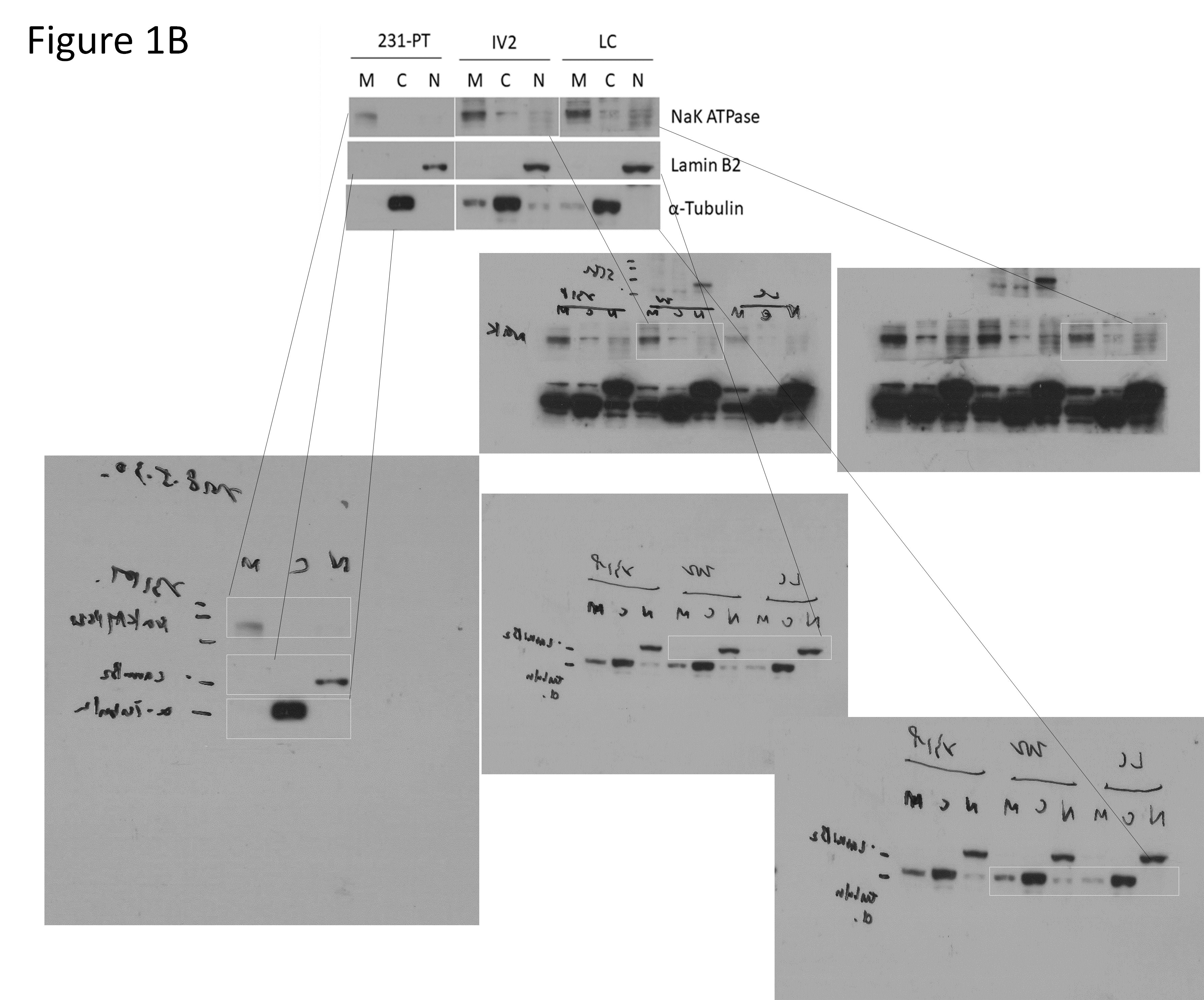


**Fig. S1. Uncropped western blot images. Cont.**


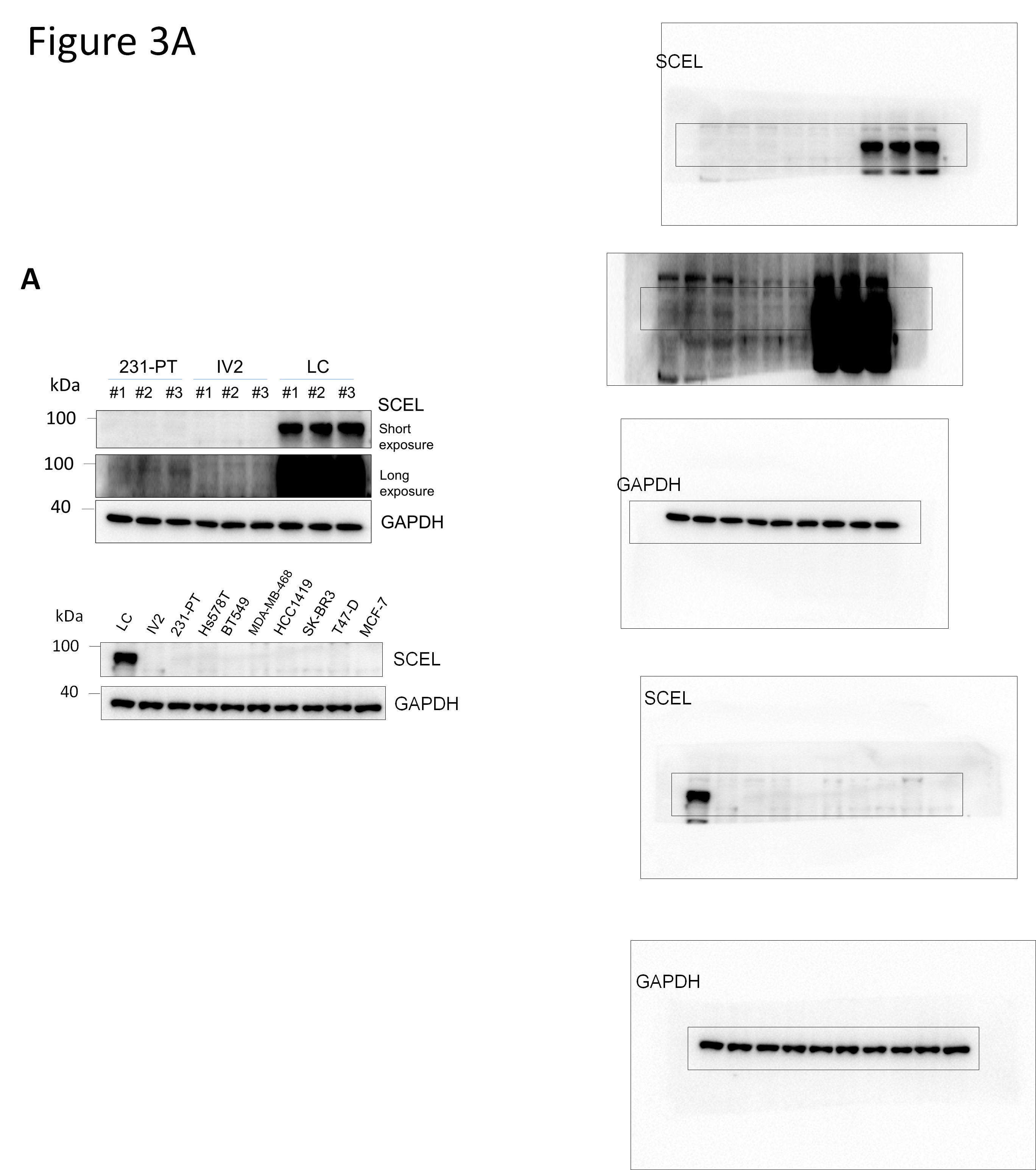


**Fig. S1. Uncropped western blot images. Cont.**


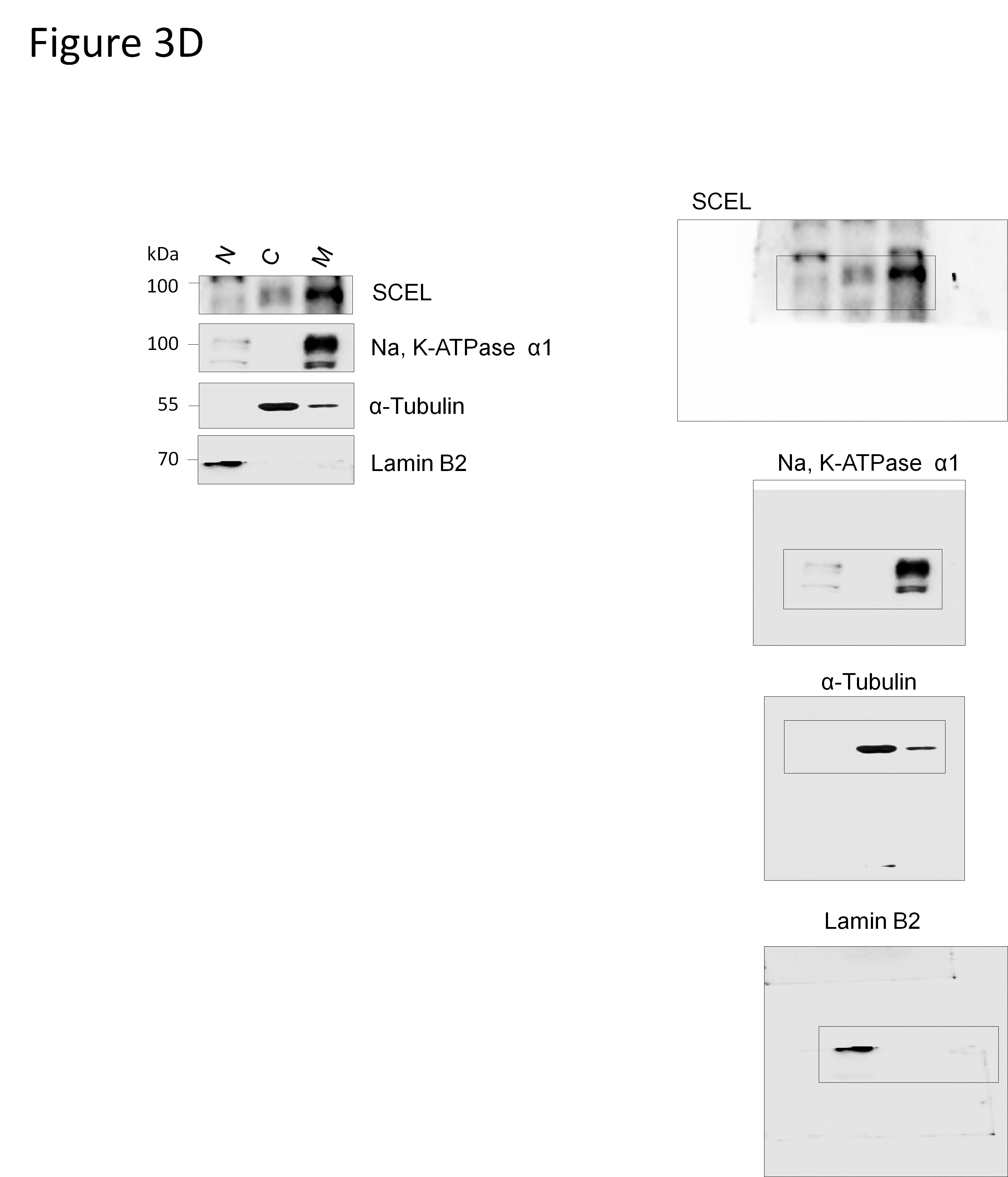


**Fig. S1. Uncropped western blot images. Cont.**


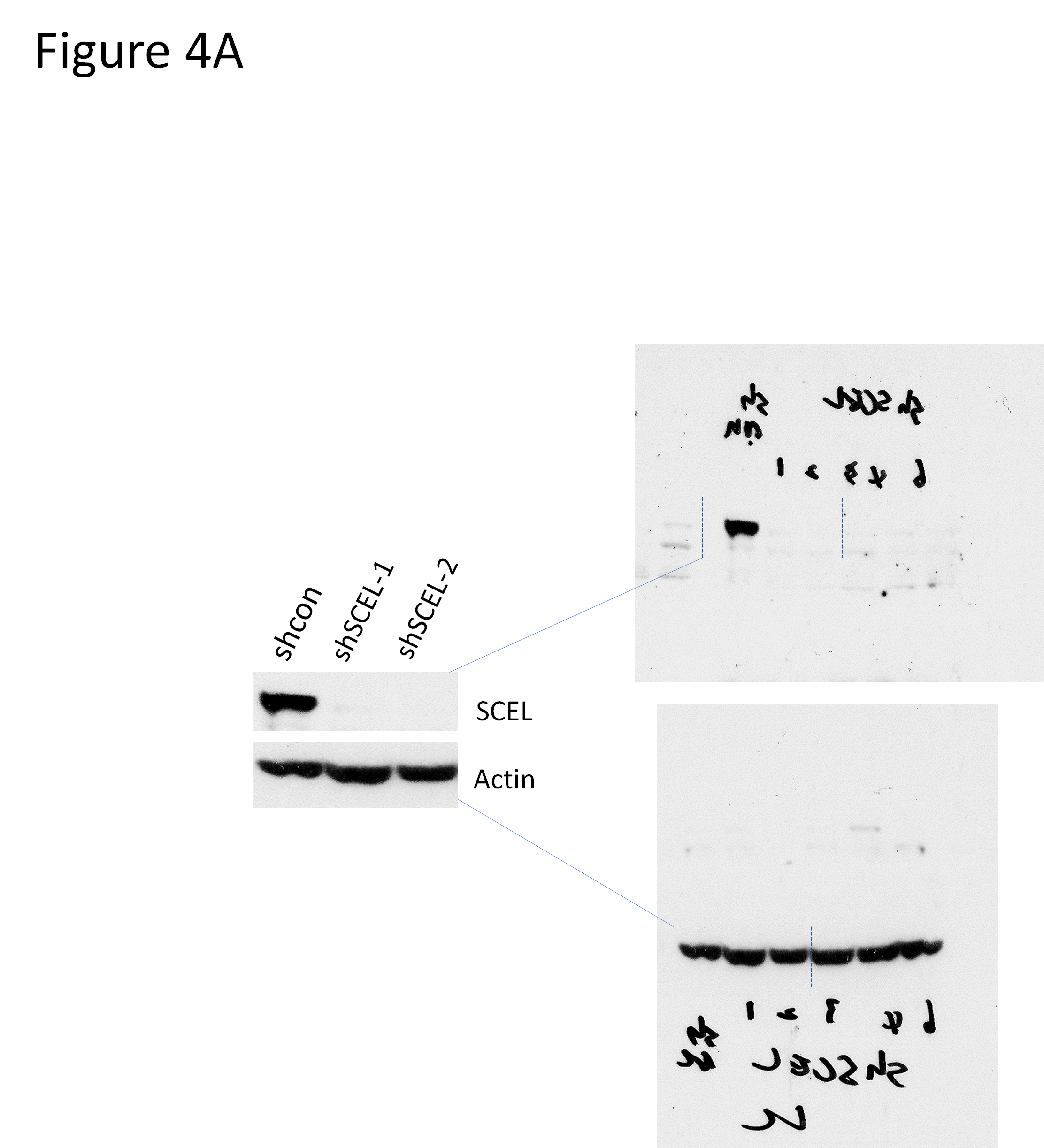


**Fig. S1. Uncropped western blot images. Cont.**


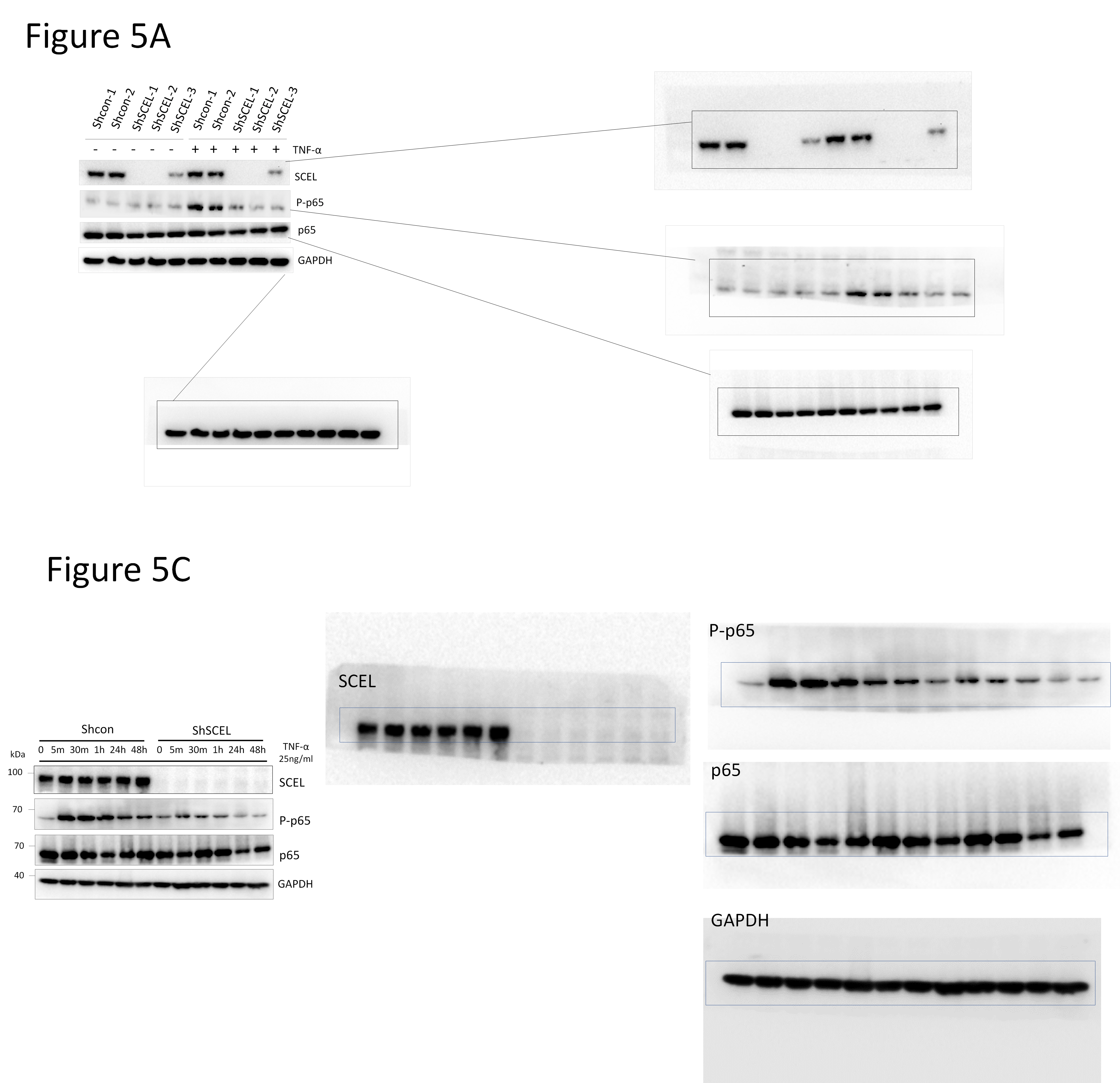


**Fig. S1. Uncropped western blot images. Cont.**


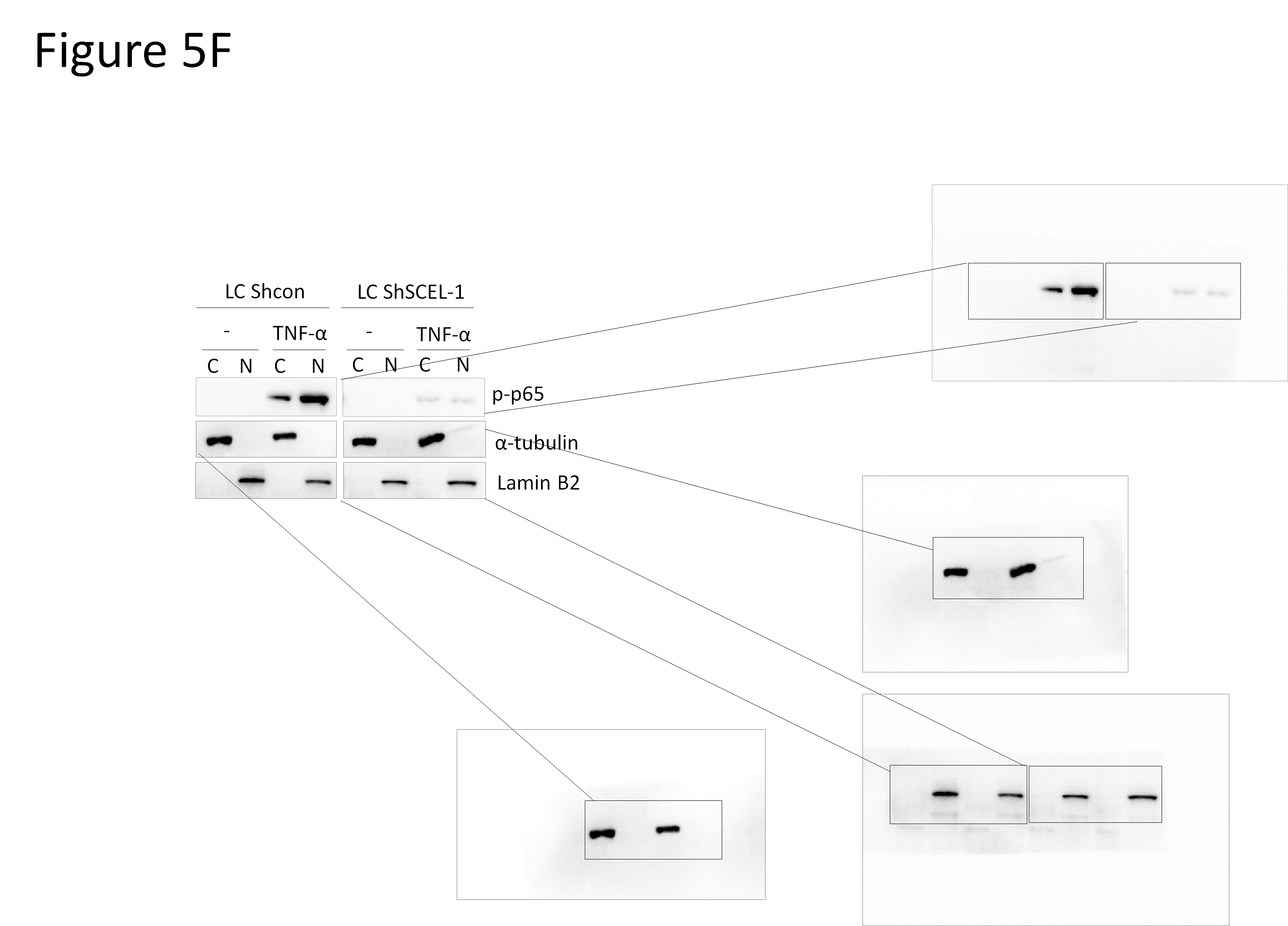


**Fig. S1. Uncropped western blot images. Cont.**


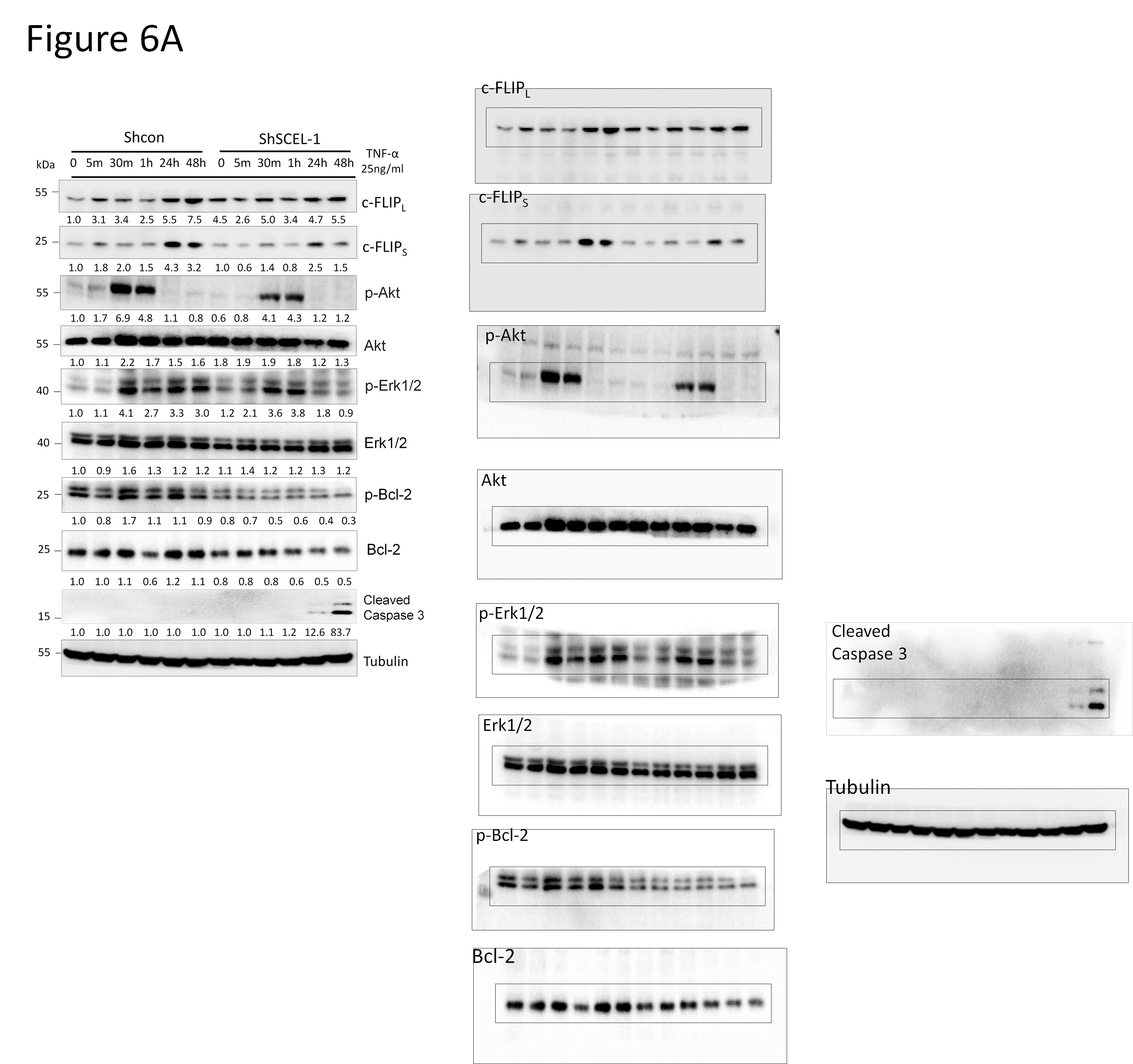


**Fig. S1. Uncropped western blot images. Cont.**


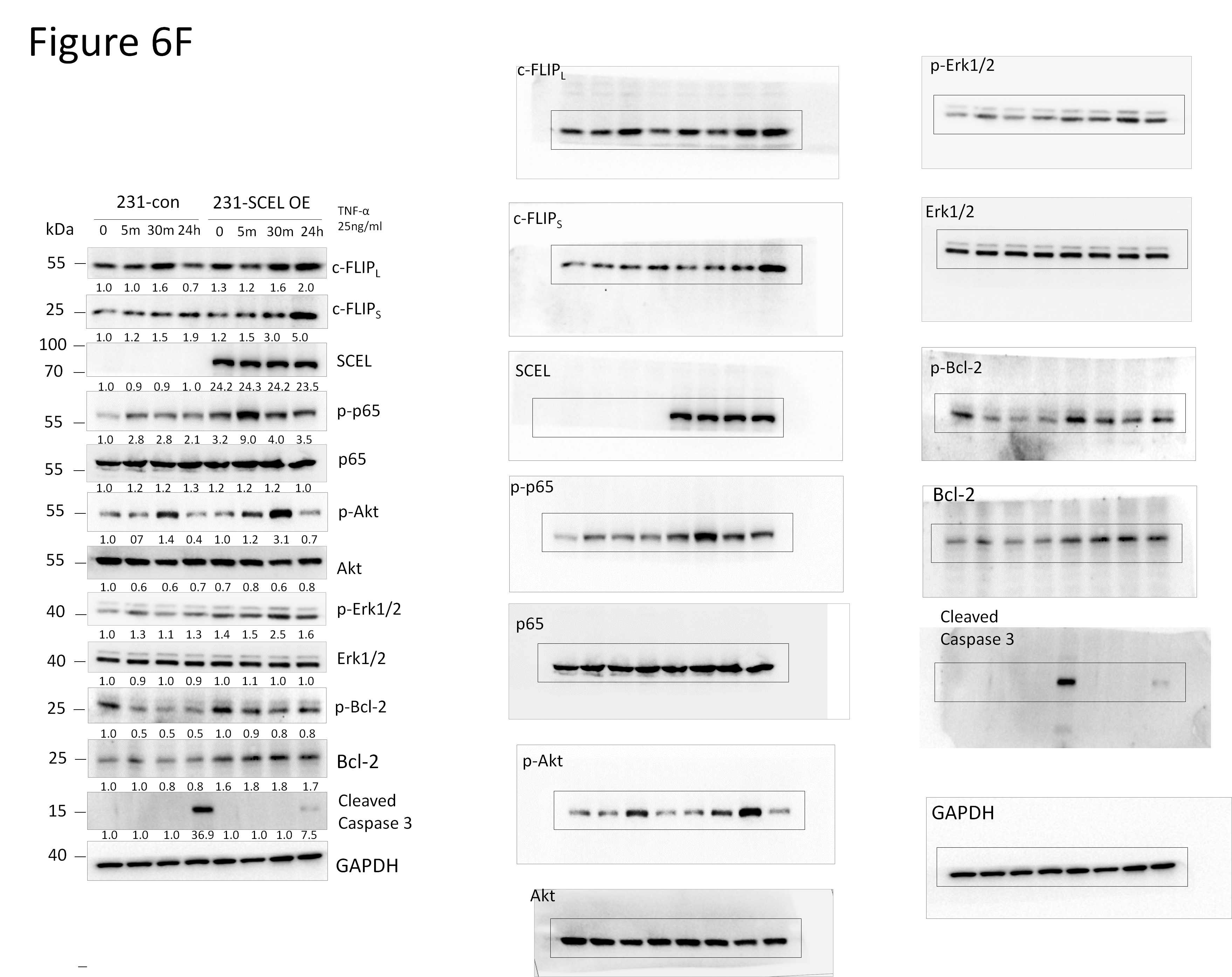


**Fig. S1. Uncropped western blot images. Cont.**


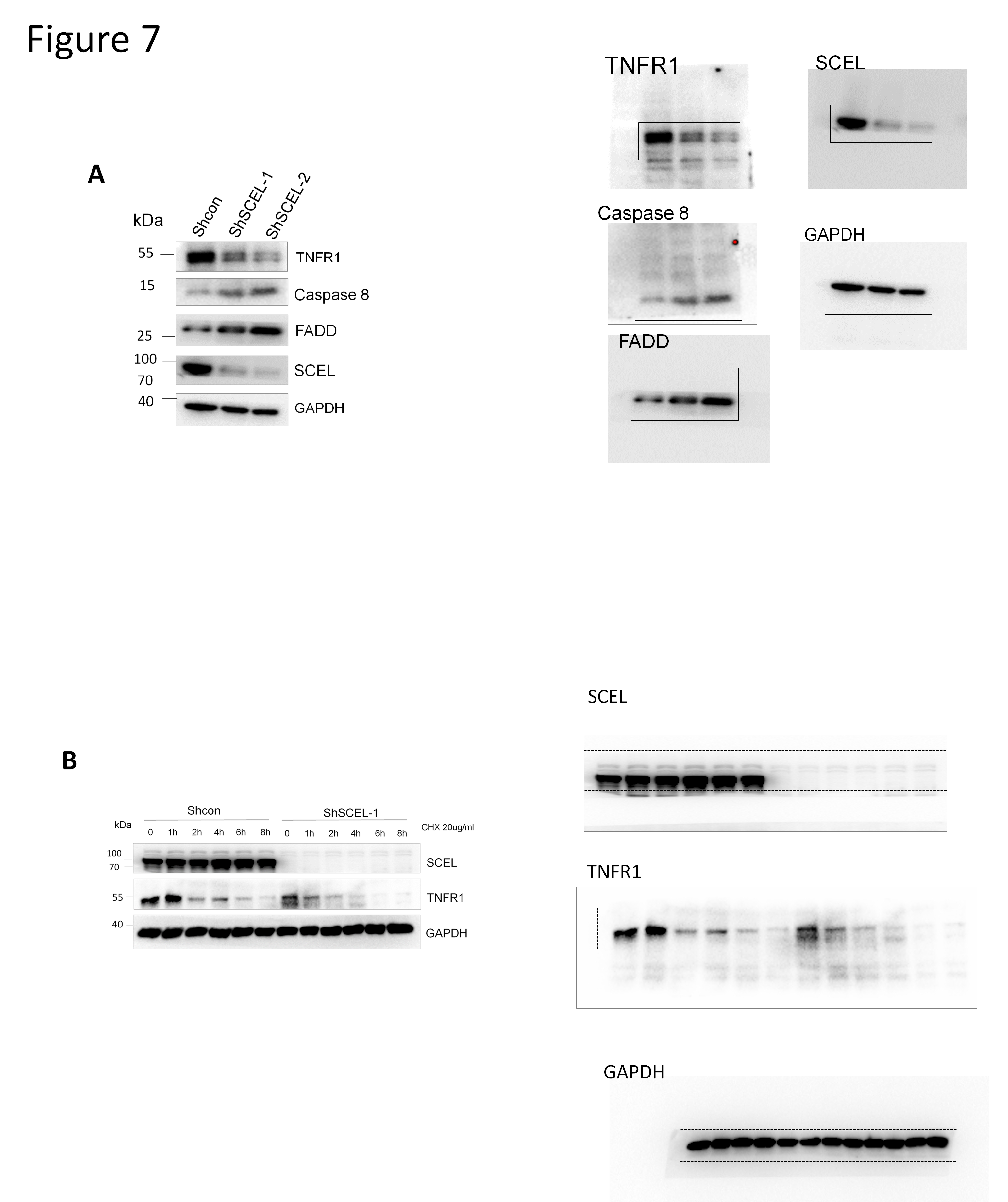


**Fig. S1. Uncropped western blot images. Cont.**


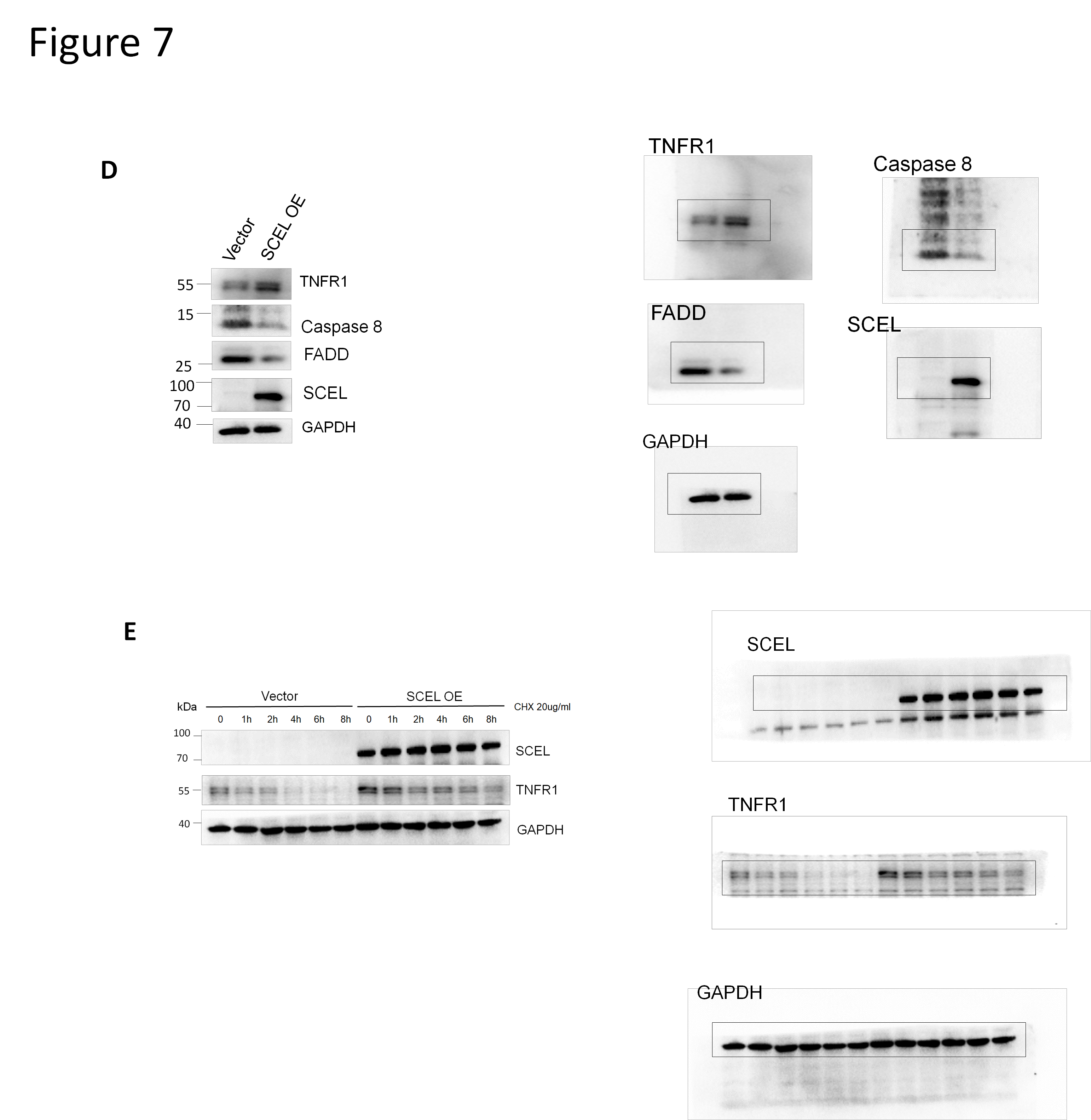


**Fig. S1. Uncropped western blot images. Cont.**


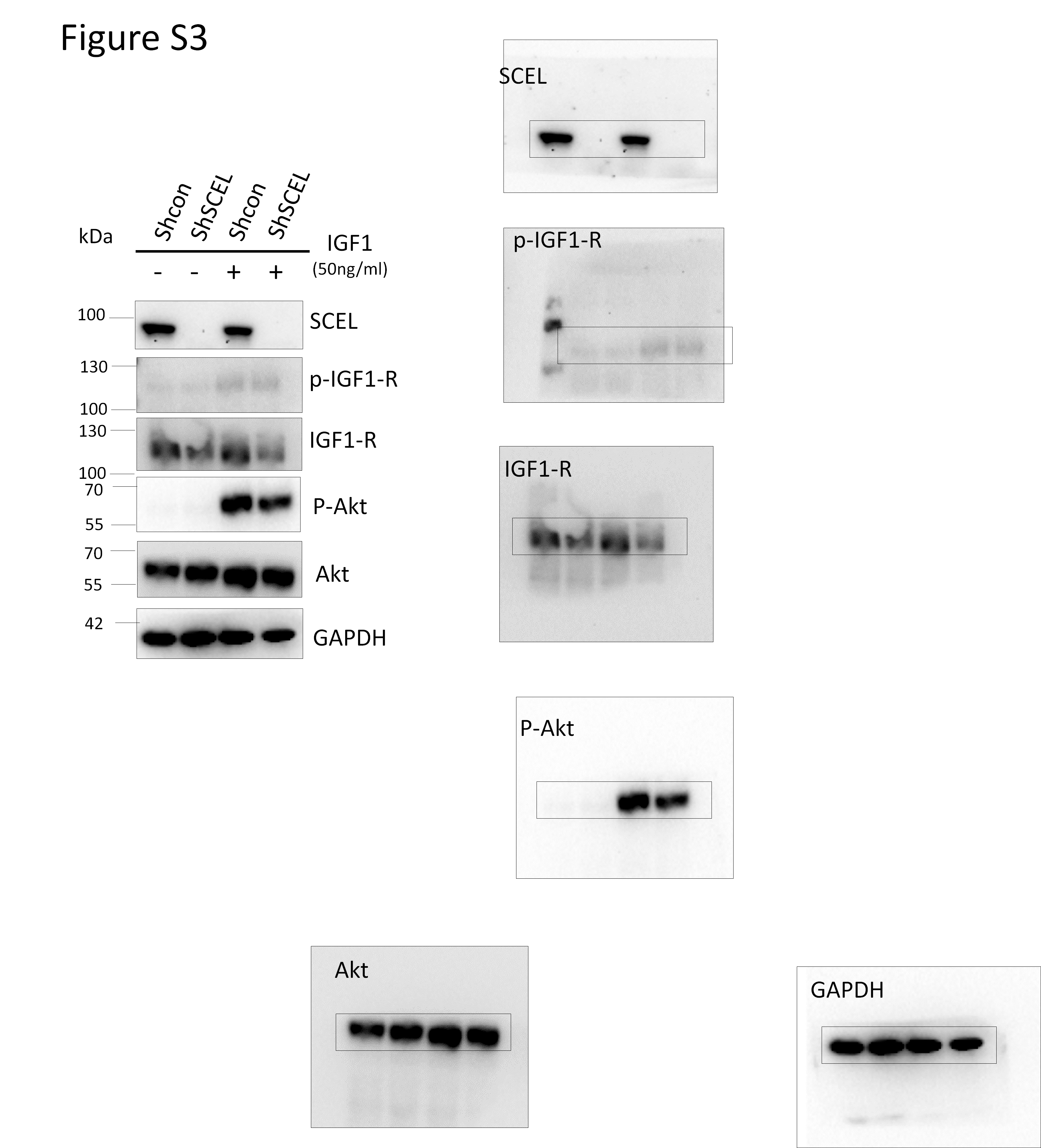


**Fig. S1. Uncropped western blot images. Cont.**


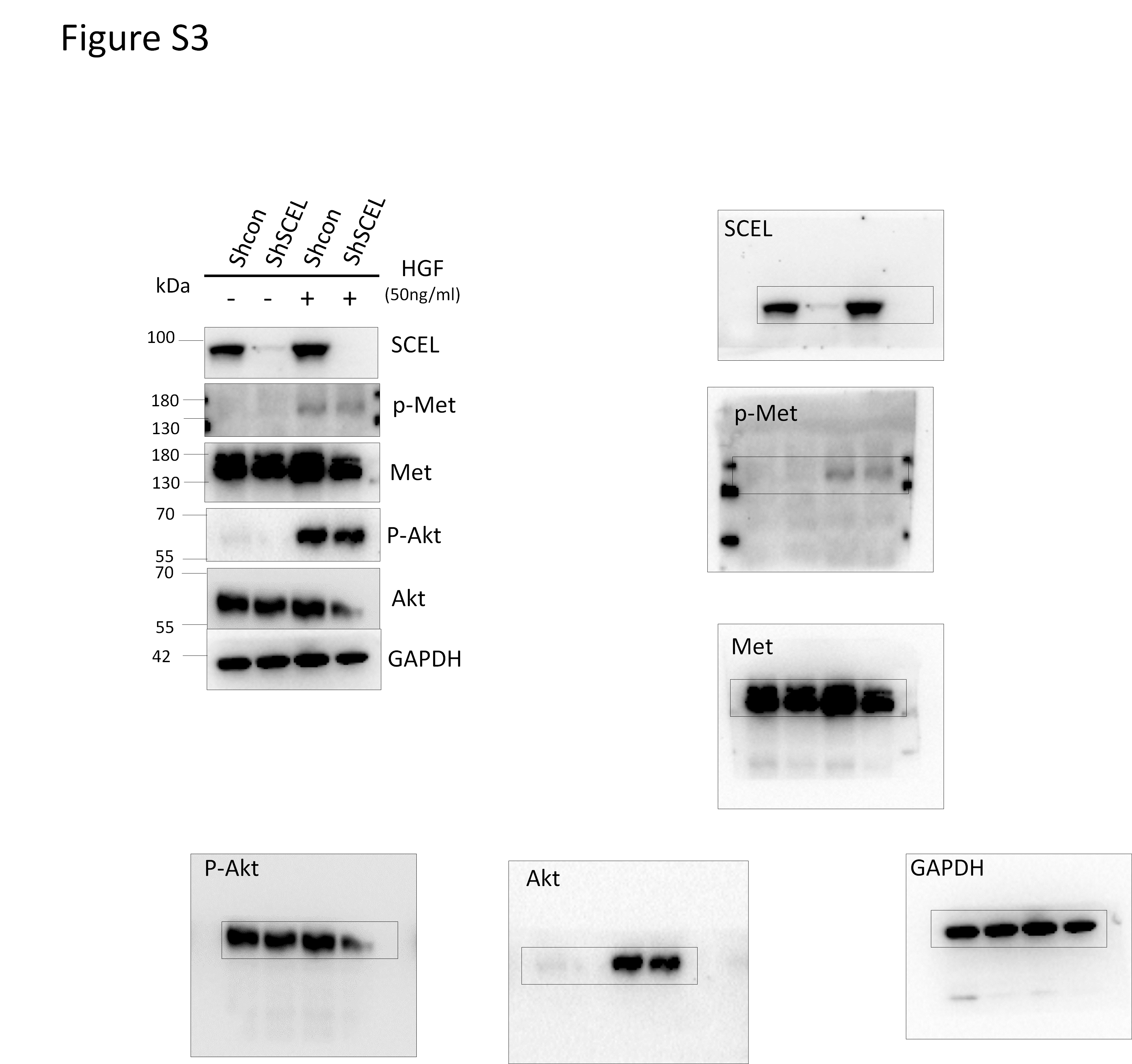


**Fig. S1. Uncropped western blot images. Cont.**


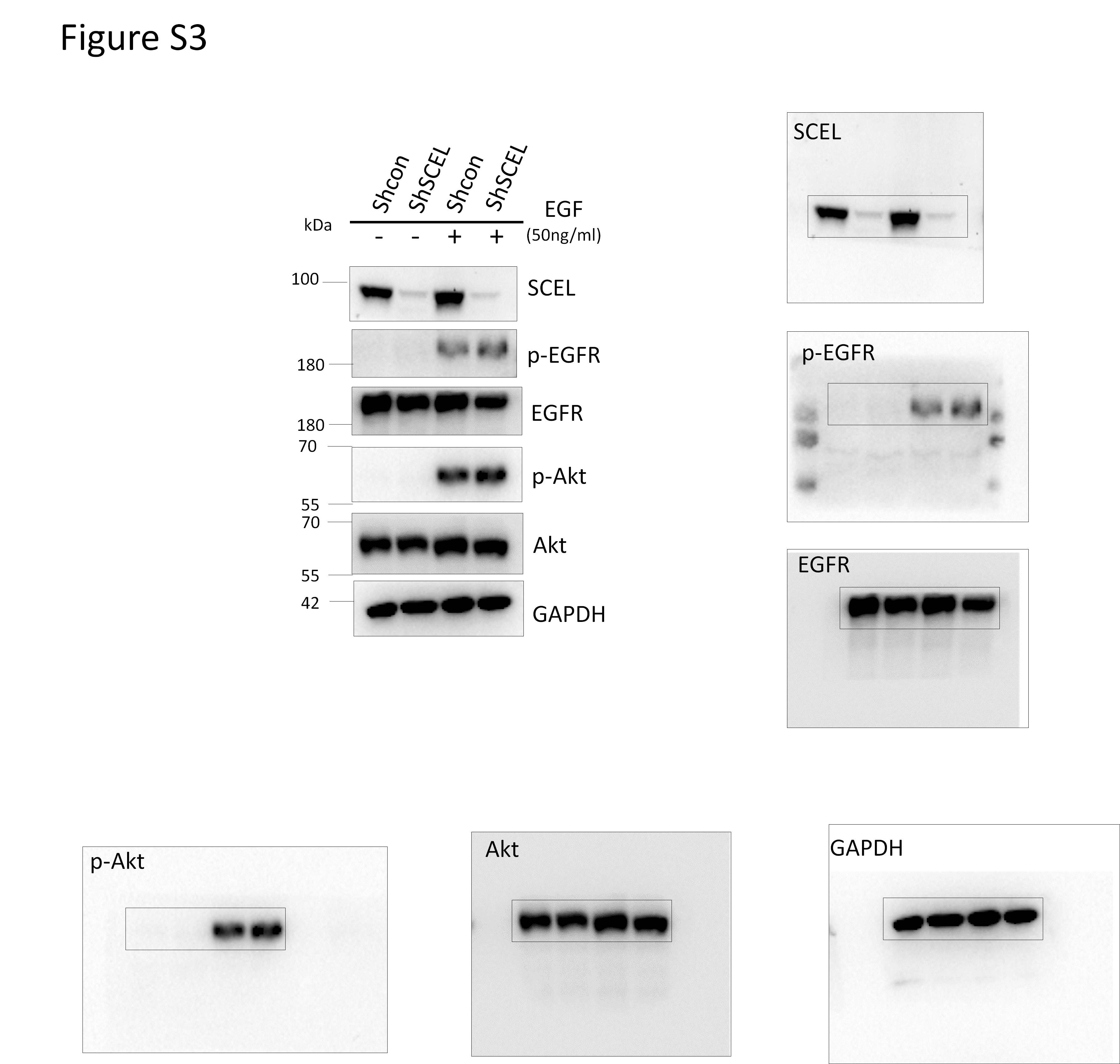


**Fig. S1. Uncropped western blot images. Cont.**


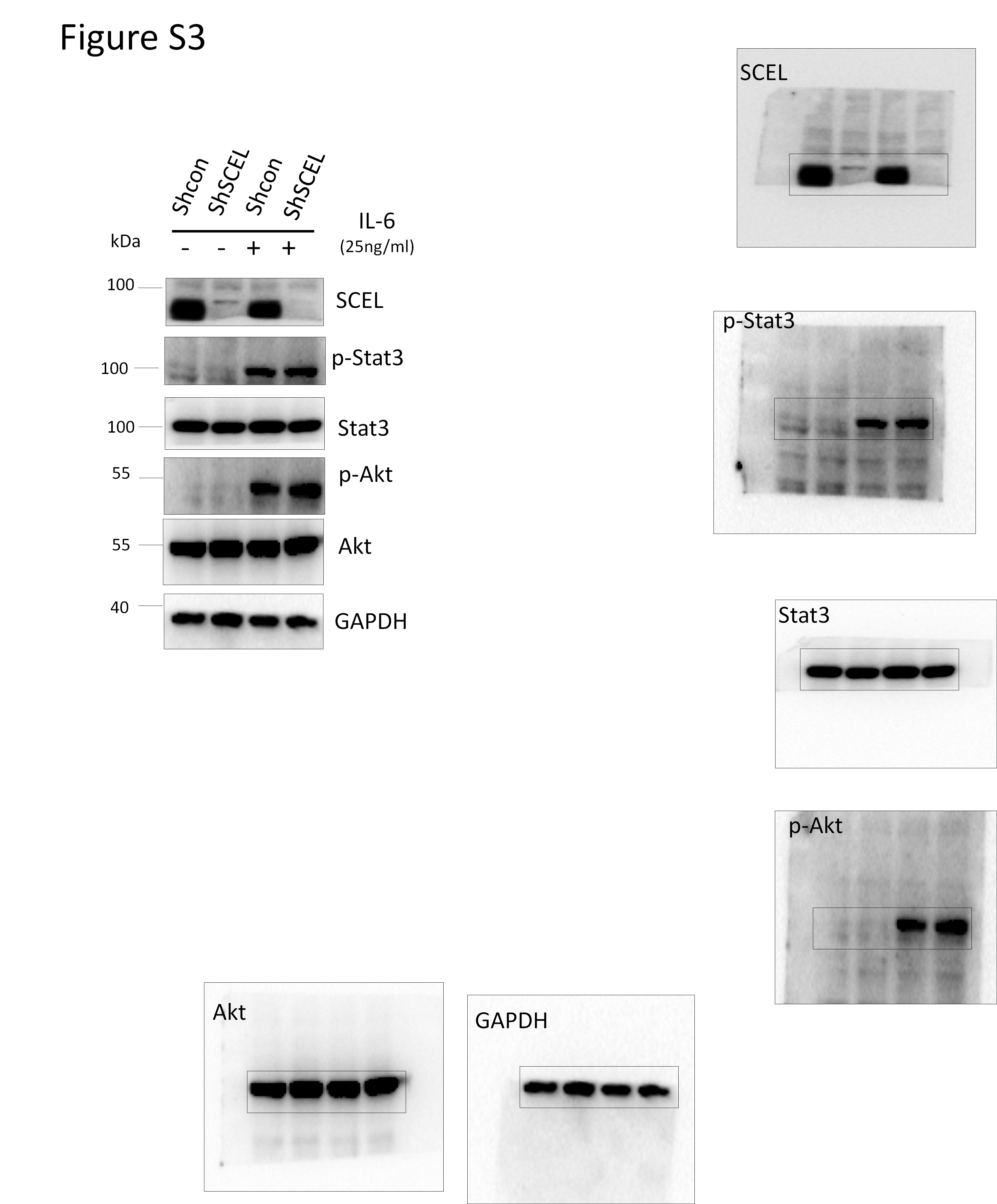


**Fig. S1. Uncropped western blot images. Cont.**


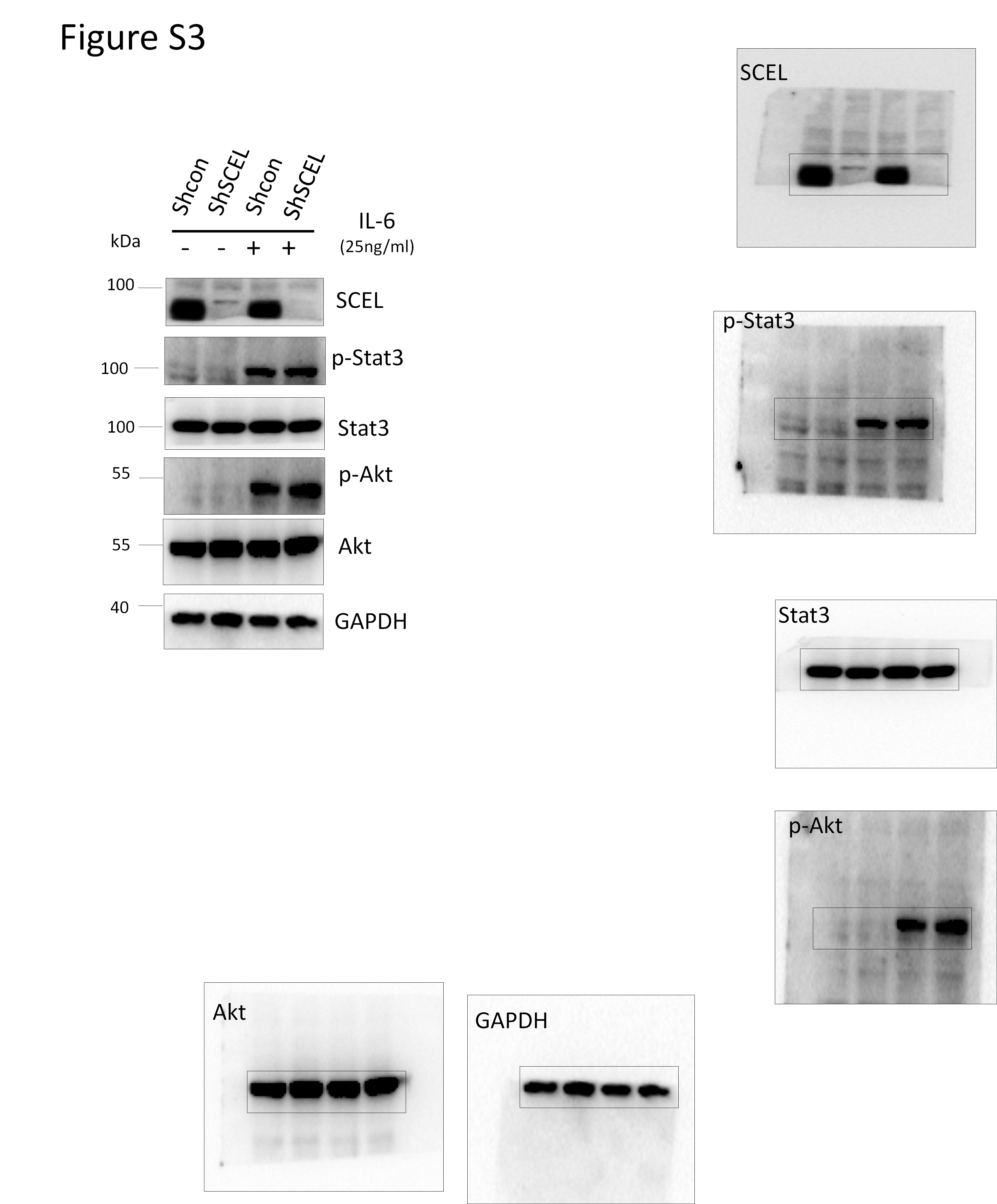

Supplement: Supplementary file 3 — Additional file 3: Fig. S1. Uncropped Western blots. [file 12929_2023_986_MOESM3_ESM.docx]
